# Organometallic Intermediates in the Synthesis of Photoluminescent Zirconium and Hafnium Complexes with Pyridine Dipyrrolide Ligands

**DOI:** 10.1021/acs.organomet.3c00058

**Published:** 2023-03-10

**Authors:** Dylan C. Leary, Yu Zhang, Jose G. Rodriguez, Novruz G. Akhmedov, Jeffrey L. Petersen, Brian S. Dolinar, Carsten Milsmann

**Affiliations:** C. Eugene Bennett Department of Chemistry, West Virginia University, Morgantown, West Virginia 26506, United States

## Abstract

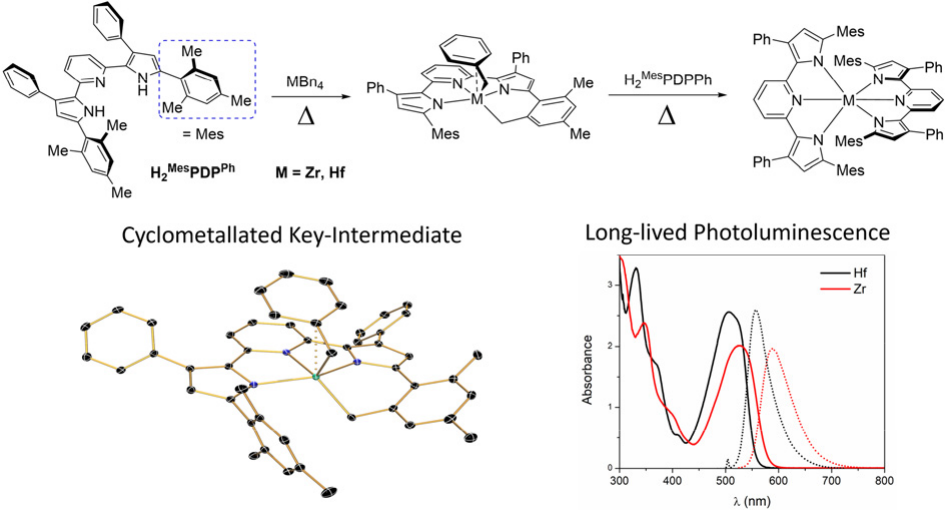

The two commercially available zirconium complexes tetrakis(dimethylamido)zirconium, Zr(NMe_2_)_4_, and tetrabenzylzirconium, ZrBn_4_, were investigated for their utility as starting materials in the synthesis of bis(pyridine dipyrrolide)zirconium photosensitizers, Zr(PDP)_2_. Reaction with one equivalent of the ligand precursor 2,6-bis(5-methyl-3-phenyl-1*H*-pyrrol-2-yl)pyridine, H_2_^Me^PDP^Ph^, resulted in the isolation and structural characterization of the complexes (^Me^PDP^Ph^)Zr(NMe_2_)_2_thf and (^Me^PDP^Ph^)ZrBn_2_, which could be converted to the desired photosensitizer Zr(^Me^PDP^Ph^)_2_ upon addition of a second equivalent of H_2_^Me^PDP^Ph^. Using the more sterically encumbered ligand precursor 2,6-bis(5-(2,4,6-trimethylphenyl)-3-phenyl-1*H*-pyrrol-2-yl)pyridine, H_2_^Mes^PDP^Ph^, only ZrBn_4_ yielded the desired bis-ligand complex Zr(^Mes^PDP^Ph^)_2_. Careful monitoring of the reaction at different temperatures revealed the importance of the organometallic intermediate (cyclo-^Mes^PDP^Ph^)ZrBn, which was characterized by X-ray diffraction analysis and ^1^H NMR spectroscopy and shown to contain a cyclometalated ^Mes^PDP^Ph^ unit. Taking inspiration from the results for zirconium, syntheses for two hafnium photosensitizers, Hf(^Me^PDP^Ph^)_2_ and Hf(^Mes^PDP^Ph^)_2_, were established and shown to proceed through similar intermediates starting from tetrabenzylhafnium, HfBn_4_. Initial studies of the photophysical properties of the photoluminescent hafnium complexes indicate similar optical properties compared to their zirconium analogues.

## Introduction

Photoluminescent transition metal complexes with long excited state lifetimes have been intensely studied due to their important role in solar energy conversion,^1−4^ photocatalysis,^5−14^ organic light-emitting diodes (OLEDs),^15−17^ and biomedical applications.^18^ Traditionally, many of the most prominent representatives of this class of compounds have relied on precious metals that impart favorable photophysical and chemical properties to the resulting complexes.^14,19−23^ Among the advantageous photophysical properties of precious metal chromophores compared to their first-row metal congeners are rapid intersystem crossing rates^24,25^ to long-lived excited states due to the strong spin–orbit coupling in second- and third-row metal complexes and reduced nonradiative relaxation through destabilization of metal-centered excited states.^26,27^ A major benefit in terms of their chemical stability is the lower propensity of precious metal ions compared to first-row and early transition metals to form oxides or hydroxides,^28^ which allows for more straightforward synthesis, handling, and processing under regular benchtop conditions in the presence of molecular oxygen and/or water. Obvious downsides of precious metal chromophores include the high cost and relatively limited overall supply, both driven by the low abundance of precious metals in the Earth’s crust,^29^ as well as their relatively high toxicity compared to base metals.^30,31^ Driven by an increasing interest in and demand for large scale photophysical and photochemical applications such as cost-efficient solar energy conversion and energy-efficient lighting and display technologies, the development of photoactive complexes containing Earth-abundant metals has become an area of intense research.^32−34^

Despite their relatively high abundance, early transition metals have been underappreciated in the design of new photoactive coordination compounds. This can largely be attributed to the electron-poor nature of these metals, which favor high oxidation states and electronic structures with low d^n^ electron configurations. These specific properties demand fundamentally different design principles for the development of early transition metal chromophores compared to those established for photoactive complexes based on electron-rich precious metal ions. For complexes with a d^0^ ground state electron configuration, commonly encountered for group 3–5 elements, excited states with ligand-to-metal charge transfer (LMCT) character are particularly attractive, as metal-to-ligand charge transfer^35−37^ or metal-centered d-d excited states,^38−43^ which have been intensively studied for more electron-rich chromophores, are not available. As an added benefit, the absence of metal-centered excited states in d^0^ metal ions eliminates deleterious nonradiative decay pathways often encountered for higher d^n^ configurations,^44^ resulting in longer excited state lifetimes and increased photoluminescence quantum yields. Early examples of photoluminescent d^0^ systems with LMCT excited states include group 3 and 4 metallocene complexes^45−57^ and group 4 and 5 imido species.^58,59^ Owing to advances in the development of group 4 metallocene catalysts for polymerization reactions,^60,61^ the optical properties of emissive zirconocene and hafnocene species have been steadily improved by the introduction of more rigid cyclopentadienyl systems, for example in *ansa*-metallocenes,^54^ and continue to be studied for photochemical applications.^62,63^

**Scheme 1:**
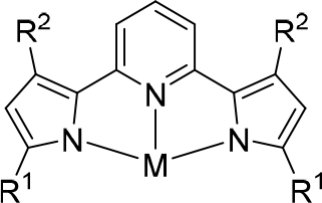
Structure of [^R1^PDP^R2^]^2–^ Ligands Indicating the Positions of the R^1^ and R^2^ Substituents on the Pyrrolide Rings

More recently, d^0^ chromophores with electron-rich pincer ligands have garnered increased attention.^64^ The availability of ligands with a wide variety of different donor atoms combined with the high structural rigidity imparted by the pincer framework makes this class of compounds a prime target for the development of new photoluminescent molecules. In this arena, we have demonstrated that zirconium complexes with two pyridine dipyrrolide (PDP) ligands exhibit photoluminescence with a long lifetime and high quantum yield by thermally activated delayed fluorescence (TADF) emanating from excited states with significant LMCT character.^65−67^ Additionally, we showed that the complexes Zr(^R1^PDP^R2^)_2_, where [^R1^PDP^R2^]^2–^ = 2,6-bis(5-R^1^-3-R^2^-pyrrol-2-ide)pyridine (Scheme 1), can replace or even outcompete precious metal photosensitizers in visible light photoredox catalysis^65−68^ and photon upconversion.^69^

**Figure 1:**
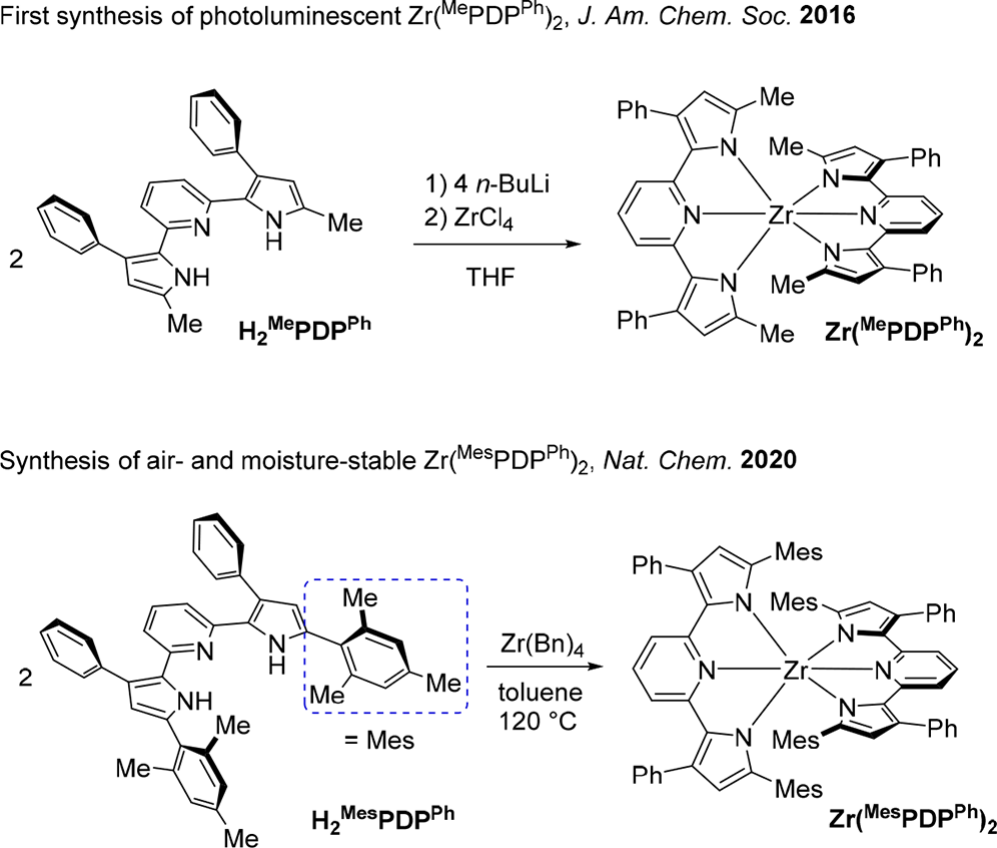
Previously reported syntheses of bis(pyridine dipyrrolide)zirconium photosensitizers according to refs (65) (top) and (67) (bottom).

Importantly, the substituents on the PDP framework have a significant influence on the optical and chemical properties of the Zr(^R1^PDP^R2^)_2_ complexes.^68^ The R^1^ substituents in particular play a critical role by limiting the number of available coordination sites at the zirconium center, increasing the overall rigidity of the molecular structure, and improving the chemical stability with respect to decomposition by hydrolysis of the Zr–N bonds.^67^ Notably, [^R1^PDP^R2^]^2–^ ligands with different R^1^ substituents require different synthetic approaches to obtain the corresponding Zr(^R1^PDP^R2^)_2_ complexes (Figure 1). Our initial reports for Zr(^Me^PDP^R2^)_2_ (R^2^ = H, Me, Ph, C_6_F_5_) utilized a straightforward salt metathesis route using Li_2_^Me^PDP^R2^, obtained in situ by deprotonation of H_2_^Me^PDP^R2^, and commercially available ZrCl_4_.^65^ In contrast, preparation of the air- and moisture-stable derivative Zr(^Mes^PDP^Ph^)_2_ (Mes = 2,4,6-trimethylphenyl), incorporating significantly bulkier mesityl substituents, required the use of the light- and temperature-sensitive organometallic precursor tetrabenzylzirconium, ZrBn_4_, as no product could be obtained using the salt metathesis approach.^67^ In this study, we explore different synthetic approaches toward the synthesis of Zr(^R1^PDP^R2^)_2_ complexes and characterize important intermediates observed during the reactions. We furthermore show that the synthetic methods derived for the zirconium systems can be utilized to generate the photoluminescent hafnium complexes Hf(^Me^PDP^Ph^)_2_ and Hf(^Mes^PDP^Ph^)_2_.

## Results and Discussion

### Reactivity of H_2_^R1^PDP^Ph^ (R^1^ = Me or Mes) with Zr(NMe_2_)_4_

**Scheme 2:**
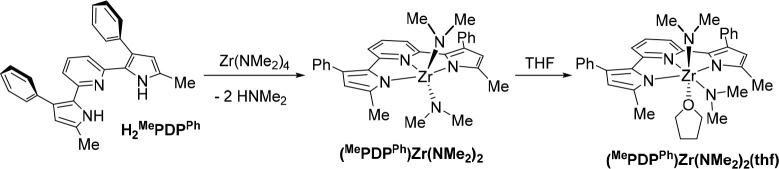
Synthesis of (^Me^PDP^Ph^)Zr(NMe_2_)_2_(thf)

Taking inspiration from the successful synthesis of Zr(^Mes^PDP^Ph^)_2_ using tetrabenzylzirconium, during which the benzyl ligands of the zirconium starting material act as internal bases to deprotonate the ligand precursor H_2_^Mes^PDP^Ph^, we were curious whether commercially available Zr(NMe_2_)_4_ could fulfill a similar role in the synthesis of Zr(^R1^PDP^Ph^)_2_ photosensitizers. We envisioned that this new approach would provide benefits for complexes with both methyl and mesityl substituents in the 5-position of the pyrrolide rings by avoiding the need for removal of solid byproducts (LiCl) and circumventing the laborious synthesis of light- and temperature-sensitive ZrBn_4_, respectively. The combination of two equivalents of H_2_^Me^PDP^Ph^ and Zr(NMe_2_)_4_ in benzene at room temperature provided a mixture of H_2_^Me^PDP^Ph^ and the new zirconium species (^Me^PDP^Ph^)Zr(NMe_2_)_2_ in a 1:1 ratio, as determined by ^1^H NMR spectroscopy. Dimethylamine was observed as the only byproduct of the reaction. Heating the mixture to 80 °C resulted in conversion to Zr(^Me^PDP^Ph^)_2_, which crystallized from the reaction mixture. Changing the H_2_^Me^PDP^Ph^/Zr(NMe_2_)_4_ stoichiometry from 2:1 to 1:1 allowed the isolation of the mono-PDP complex (^Me^PDP^Ph^)Zr(NMe_2_)_2_(thf) in 47% yield following recrystallization by slow diffusion of pentane into a concentrated THF solution of the complex (Scheme 2). All attempts to obtain the solvent-free complex (^Me^PDP^Ph^)Zr(NMe_2_)_2_ as a pure solid remained unsuccessful.

**Figure 2:**
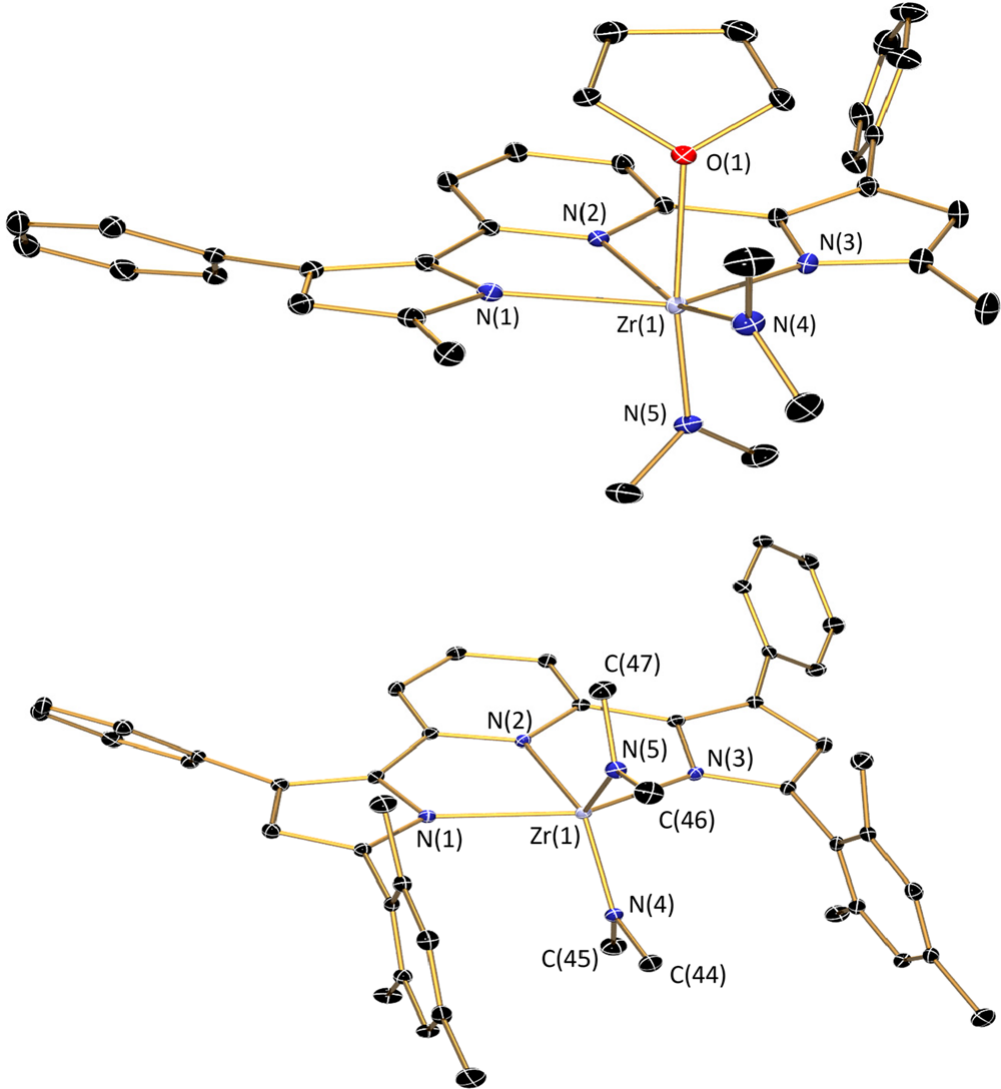
Molecular structures of (^Me^PDP^Ph^)Zr(NMe_2_)_2_(thf), top, and (^Mes^PDP^Ph^)Zr(NMe_2_)_2_, bottom, shown with 30% probability ellipsoids. Hydrogen atoms are omitted for clarity.

The molecular structure of (^Me^PDP^Ph^)Zr(NMe_2_)_2_(thf) is shown in Figure 2 and contains a distorted octahedral coordination environment around the Zr^IV^ center. The Zr–N_pyridine_ and Zr–N_pyrrolide_ bonds are slightly longer than the corresponding bonds in Zr(^Me^PDP^Ph^)_2_ (Table 1), which is most likely due to the influence of the strongly π-donating dimethylamido ligands that attenuate π-donation from the pincer ligand. Consistent with this interpretation, the Zr–N_amido_ bonds are significantly shorter compared to those of the pincer ligand, indicating increased double-bond character. The distorted octahedral geometry is completed by a THF ligand that is only weakly bound, reflected in a relatively long Zr–O bond. The weak nature of the Zr–O bond was also confirmed by ^1^H NMR spectroscopy, which revealed only a single resonance for the methyl protons of the two dimethylamido ligands and effective *C*_2v_ symmetry of (^Me^PDP^Ph^)Zr(NMe_2_)_2_(thf), which is inconsistent with the solid state structure and could imply loss of the THF ligand in solution (Figure S1). However, the two resonances for the THF ligand at 3.32 and 1.10 ppm are clearly shifted from those expected for free THF in benzene-*d*_6_ solution, and a 1D-NOESY experiment confirmed through space interactions between the THF protons in the 2,5-positions and the dimethylamido and pyrrolide methyl protons (Figure S1). Taken together, the NMR spectroscopic data suggest a dynamic equilibrium between five- and six-coordinate zirconium complexes that interconvert rapidly on the NMR time scale by dissociation and reassociation of the THF ligand. An alternative explanation is the formation of a *C*_2v_-symmetric isomer in solution, in which the THF molecule is bound *trans* to the pyridine of the PDP ligand. While this possibility cannot be ruled out based on the NMR data, this *C*_2v_-symmetric geometry should be thermodynamically unfavorable in our opinion, because it forces the two π-donating dimethylamido ligands into *trans*-coordination, which would weaken their π-interations with the zirconium center.

**Table 1:** Comparison of the Metal–Ligand Bond Lengths (Å) in Zirconium Pyridine Dipyrrolide Complexes^a^

	(^Me^PDP^Ph^)Zr(NMe_2_)_2_(thf)	(^Mes^PDP^Ph^)Zr(NMe_2_)_2_	(^Me^PDP^Ph^)ZrBn_2_	Zr(^Me^PDP^Ph^)_2_	Zr(^Mes^PDP^Ph^)_2_
Zr(1)–N(1)	2.2246(19)	2.1852(12)	2.157(4)	2.151(3)	2.170(2)
				2.143(3)	2.170(2)
Zr(1)–N(2)	2.3270(19)	2.2748(13)	2.265(3)	2.288(3)	2.262(2)
				2.300(3)	2.262(2)
Zr(1)–N(3)	2.219(2)	2.1822(13)	2.161(4)	2.183(3)	2.166(2)
				2.171(3)	2.165(2)
Zr(1)–N(4)	2.090(2)	2.0017(15)	–	–	–
Zr(1)–N(5)	2.028(2)	2.0129(15)	–	–	–
Zr(1)–O(1)	2.4199(18)	–	–	–	–
Zr(1)–C(28)	–	–	2.235(5)	–	–
Zr(1)–C(35)	–	–	2.21(3)	–	–

a Values for Zr(^Me^PDP^Ph^)_2_ and Zr(^Mes^PDP^Ph^)_2_ are taken from references (65) and (67), respectively.

**Scheme 3:**
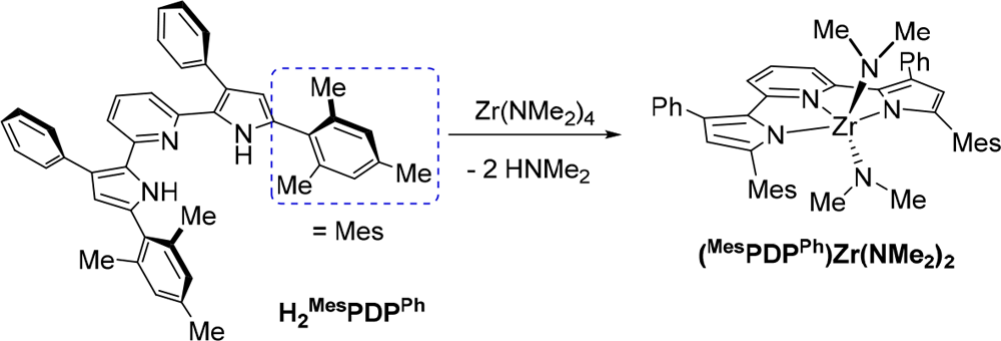
Synthesis of (^Mes^PDP^Ph^)Zr(NMe_2_)_2_

Using the more sterically demanding ligand precursor H_2_^Mes^PDP^Ph^, slightly different outcomes were observed. Addition of one equivalent of H_2_^Mes^PDP^Ph^ to Zr(NMe_2_)_4_ at room temperature resulted in clean conversion to the five-coordinate complex (^Mes^PDP^Ph^)Zr(NMe_2_)_2_ (74% isolated yield), even in the presence of coordinating solvents such as THF (Scheme 3). This result suggests that the increased steric protection of the metal center imparted by the mesityl substituents on the [^Mes^PDP^Ph^]^2–^ ligand does not allow coordination of a sixth ligand in addition to the two dimethylamido ligands. The ^1^H NMR spectrum is consistent with a *C*_2v_ symmetric structure in solution, and observation of a single resonance for the four methyl groups of the two dimethylamido ligands indicates free rotation of these ligands on the NMR time scale at room temperature (Figure S3). The molecular structure of (^Mes^PDP^Ph^)Zr(NMe_2_)_2_ obtained by single-crystal X-ray diffraction is shown in Figure 2 and is consistent with the geometry predicted by NMR spectroscopy. The Zr–N bond lengths of the pincer ligand (Table 1) are again elongated compared to the bis-ligand complex Zr(^Mes^PDP^Ph^)_2_, albeit less pronounced than for (^Me^PDP^Ph^)Zr(NMe_2_)_2_(thf). The short Zr–N_amido_ bonds are indicative of π-bonding between these ligands and the zirconium center. The two dimethylamido ligands and the pyridine unit of the pincer form a nearly perfect trigonal-planar arrangement as would be expected for a *C*_2v_-symmetric, distorted trigonal-bipyramidal structure. However, the N(2)–Zr(1)–N(5) angle between the pyridine of the pincer and one of the amido ligands is slightly smaller than the expected 120° at 117.72(6)°, while the N(2)–Zr(1)–N(4) angle with the second amido fragment is larger at 126.71(6)°. This is accompanied by a short Zr(1)–C(47) distance of only 2.854(2) Å compared to the corresponding Zr(1)–C(45) distance of 3.035(2) Å for the second dimethylamido ligand and indicates an agostic interaction between the Zr center and a C–H bond of one of the dimethylamido ligands in the solid state. This is further supported by a distortion of the corresponding NMe_2_ ligand, most easily detected by a small Zr(1)–N(5)–C(47) angle of only 109.28(11)°.

To our disappointment, (^Mes^PDP^Ph^)Zr(NMe_2_)_2_ proved to be unreactive toward a second equivalent of H_2_^Mes^PDP^Ph^. This observation establishes that Zr(NMe_2_)_4_ is not a suitable starting material for the synthesis of air- and moisture-stable Zr(^Mes^PDP^Ph^)_2_. We propose that the steric environment provided by the bulky [^Mes^PDP^Ph^]^2–^ ligand in combination with the two dimethylamido ligands is too crowded to allow the introduction of a second [^Mes^PDP^Ph^]^2–^ ligand. This is consistent with the inability of (^Mes^PDP^Ph^)Zr(NMe_2_)_2_ to bind additional ligands and the excellent steric protection of the basic dimethylamido functionalities by the flanking mesityl substituents apparent from the molecular structure.

### Reactivity of H_2_^R1^PDP^Ph^ (R^1^ = Me or Mes) with ZrBn_4_

Having established that neither salt metathesis using ZrCl_4_ and in situ generated Li_2_^Mes^PDP^Ph^ nor treatment of Zr(NMe_2_)_4_ with H_2_^Mes^PDP^Ph^ allowed the synthesis of Zr(^Mes^PDP^Ph^)_2_, we sought to explore the privileged role of ZrBn_4_ as a starting material for the synthesis of Zr(^R1^PDP^Ph^)_2_ complexes. We were particularly interested in the identity of any organometallic intermediates during the formation of the bis-PDP complexes. Due to the well-established light-sensitivity of zirconium benzyl species,^70−72^ all synthetic operations were conducted under reduced lighting. Additionally, all reaction vessels were fully covered in aluminum foil to minimize exposure to light and prevent decomposition by photolytic Zr–C bond homolysis.

**Scheme 4:**
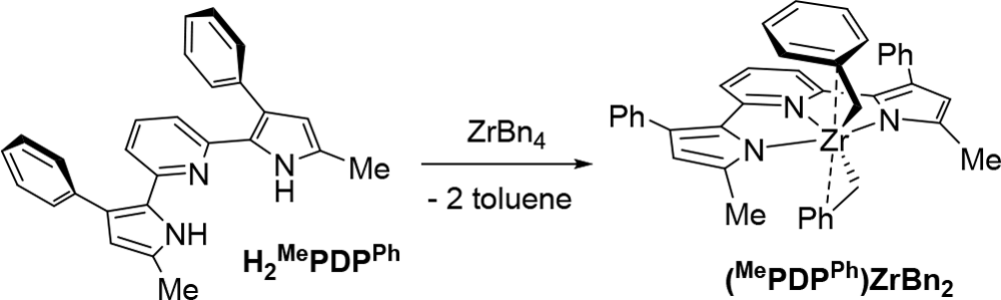
Synthesis of (^Me^PDP^Ph^)ZrBn_2_

**Figure 3:**
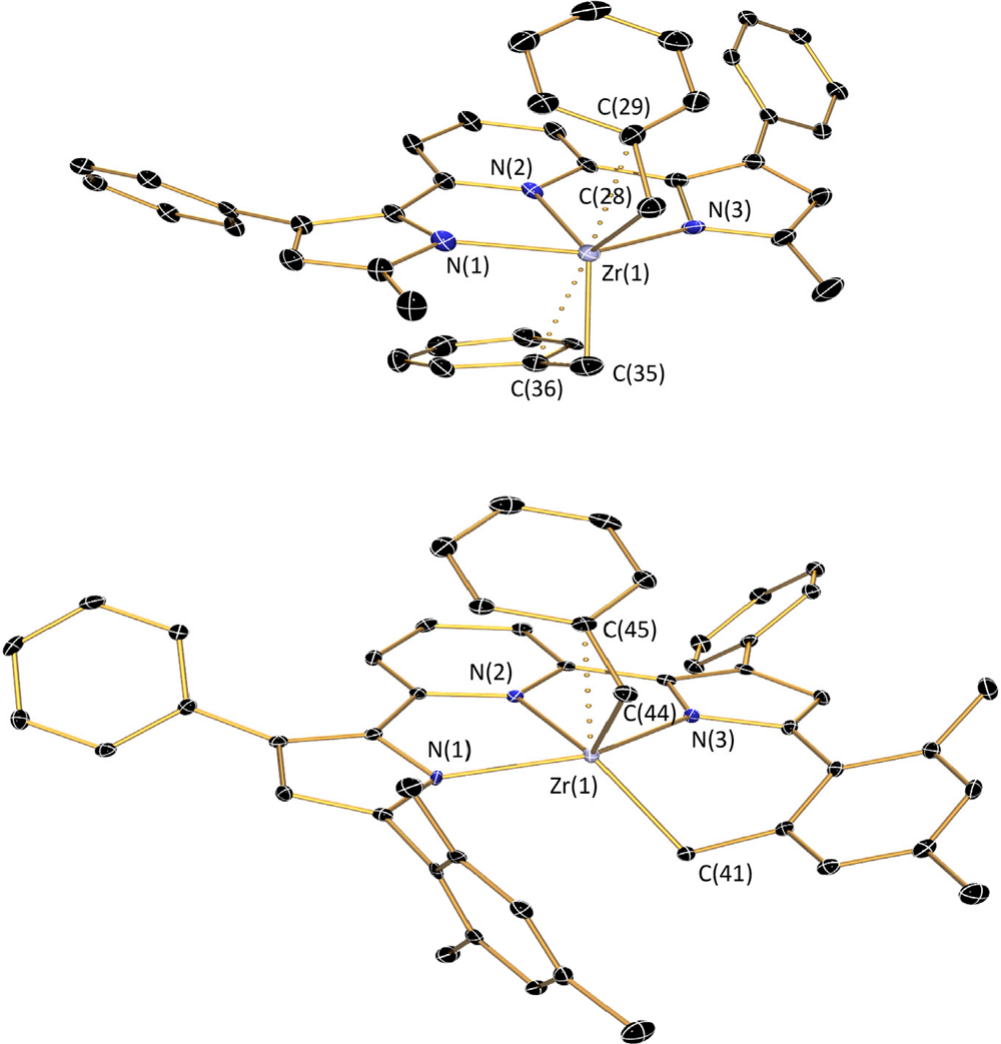
Molecular structures of (^Me^PDP^Ph^)ZrBn_2_, top, and (cyclo-^Mes^PDP^Ph^)ZrBn, bottom, shown with 30% probability ellipsoids. Hydrogen atoms are omitted for clarity.

Starting with the smaller ligand system, addition of one equivalent of H_2_^Me^PDP^Ph^ to ZrBn_4_ at room temperature resulted in the formation of a new zirconium species identified as (^Me^PDP^Ph^)ZrBn_2_ in 59% yield (Scheme 4). The molecular structure of (^Me^PDP^Ph^)ZrBn_2_ is shown in Figure 3 and reveals a five-coordinate zirconium center with two η^2^-bound benzyl ligands. The presence of η^2^-benzyl fragments is apparent from the small Zr(1)–C(28)–C(29) and Zr(1)–C(35)–C(36) angles of 90.5(3)° and 93.0(15)°, which are characteristic for this particular coordination mode in zirconium benzyl complexes.^73^ Further support for η^2^-benzyl interactions are the short Zr(1)–C(29) and Zr(1)–C(36) distances of 2.699(4) Å and 2.71(2) Å, respectively. The lower accuracy in the Zr(1)–C(36) bond length is due to disorder of this ligand. Compared to (^Me^PDP^Ph^)Zr(NMe_2_)_2_(thf), the Zr–N bonds for the pincer ligand in (^Me^PDP^Ph^)ZrBn_2_ are shortened by 5–6 pm and more closely resemble those in Zr(^Me^PDP^Ph^)_2_ (Table 1). The ^1^H NMR spectrum of (^Me^PDP^Ph^)ZrBn_2_ is consistent with the solid-state structure and a *C*_2v_ symmetric geometry in benzene-*d*_6_ solution (Figure S5). Interestingly, the signal for the 4-pyrrolide protons appears as a narrow quartet with a small coupling constant of ^4^*J* = 0.8 Hz due to coupling to the protons of the 5-methyl substituents. The coupling is not resolved for the resonance of the 5-methyl protons but manifests in a broadened signal.

No further reaction was observed upon addition of a second equivalent of H_2_^Me^PDP^Ph^ to a benzene solution of (^Me^PDP^Ph^)ZrBn_2_ at room temperature. However, subsequent heating to 80 °C produced Zr(^Me^PDP^Ph^)_2_ as a red crystalline precipitate that could be isolated by filtration.

**Scheme 5:**
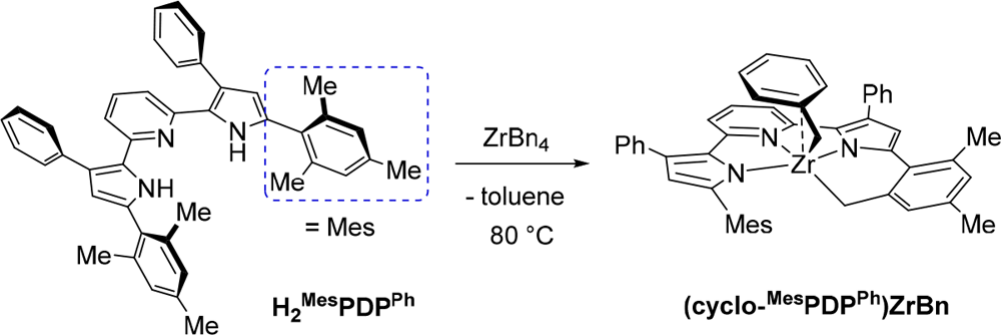
Synthesis of (Cyclo-^Mes^PDP^Ph^)ZrBn

Reactions between the bulkier ligand precursor H_2_^Mes^PDP^Ph^ and ZrBn_4_ proved to be more challenging. While no appreciable conversion of starting materials was observed at room temperature, careful monitoring of the reaction mixture by ^1^H NMR in benzene-*d*_6_ upon heating to 80 °C revealed complete consumption of ZrBn_4_ and formation of a new zirconium species as the major product after 24 h. More prolonged heating to 80 °C resulted in slow decomposition of this major product and increased formation of unidentified byproducts, while higher reaction temperatures provided Zr(^Mes^PDP^Ph^)_2_ as an additional impurity. Recrystallization from diethyl ether provided a small amount of a dark red, crystalline material identified as (cyclo-^Mes^PDP^Ph^)ZrBn by single crystal X-ray diffraction (Figure 3 and Scheme 5).

Like the bis-benzyl complex (^Me^PDP^Ph^)ZrBn_2_, (cyclo-^Mes^PDP^Ph^)ZrBn contains an η^2^-benzyl ligand as evidenced by the small Zr(1)–C(44)–C(45) angle of 90.35(18)° and a short Zr(1)–C(45) distance of 2.688(3) Å. More interestingly, C–H activation of a methyl group on one of the mesityl substituents of the [^Mes^PDP^Ph^]^2–^ pincer ligand results in a metallacyclic structure with a tetradentate [cyclo-^Mes^PDP^Ph^]^3–^ fragment. While a plausible mechanism for this cyclometalation reaction is deprotonation of a mesityl methyl group by a zirconium benzyl fragment with concomitant formation of toluene, a radical pathway via thermal Zr-benzyl bond homolysis followed by hydrogen atom abstraction and radical rebound cannot be excluded based on the available data. The Zr(1)–C(41) bond length of 2.239(3) Å in the newly formed metallacycle is identical within experimental error to the Zr(1)–C(44) bond of the η^2^-benzyl ligand (2.237(3) Å). Formation of the metallacycle results in a significant reduction in the torsion angle between the pyrrolide moiety and the cyclometalated mesityl substituent at 36.31°. In contrast, the orientation of the noncyclometalated mesityl substituent to the corresponding pyrrolide ring remains closer to perpendicular with a dihedral angle of 74.49°, which is similar to the average torsion angles in Zr(^Mes^PDP^Ph^)_2_ and (^Mes^PDP^Ph^)Zr(NMe_2_)_2_ at 84.24° and 83.16°.

The ^1^H NMR spectrum of (cyclo-^Mes^PDP^Ph^)ZrBn recorded in benzene-*d*_6_ is consistent with the *C*_1_-symmetric solid-state structure (Figure S7). The low symmetry of the complex is most clearly reflected in the presence of five singlets of equal intensity for the chemically distinct methyl groups in (cyclo-^Mes^PDP^Ph^)ZrBn. In addition, two pairs of doublets for the diastereotopic protons of the η^2^-benzyl ligand and the methylene group of the metallacycle can be observed in the aliphatic part of the spectrum. The aromatic region contains a complex array of overlapping resonances that could not be unambiguously assigned without more sophisticated NMR spectroscopic experiments. Unfortunately, the small amount of material obtained by recrystallization of (cyclo-^Mes^PDP^Ph^)ZrBn did not allow the preparation of samples with sufficient concentrations for analysis by ^13^C{^1^H} NMR or 2D NMR correlation experiments. All attempts to prepare analytically pure (cyclo-^Mes^PDP^Ph^)ZrBn on a larger scale remained unsuccessful.

The identification of (cyclo-^Mes^PDP^Ph^)ZrBn as an unexpected, isolated product of the reaction between ZrBn_4_ and H_2_^Mes^PDP^Ph^ prompted us to consider its role as a potential intermediate in the synthesis of Zr(^Mes^PDP^Ph^)_2_. Careful monitoring of a 2:1 mixture of H_2_^Mes^PDP^Ph^ and ZrBn_4_ in benzene-*d*_6_ at 120 °C, reproducing the reaction conditions described in our initial report of Zr(^Mes^PDP^Ph^)_2_, confirmed the presence of (cyclo-^Mes^PDP^Ph^)ZrBn during early stages of the reaction together with unreacted starting materials and minor amounts of the desired product. Upon completion of the synthetic procedure after 48 h of heating, the cyclometalated complex was consumed completely, leaving Zr(^Mes^PDP^Ph^)_2_ as the only product observable by ^1^H NMR spectroscopy. Based on these results, we hypothesize that (cyclo-^Mes^PDP^Ph^)ZrBn plays a key role the formation of Zr(^Mes^PDP^Ph^)_2_. The cyclometalation of one of the mesityl substituents and the associated rotation of this substituents into a more coplanar orientation with respect to the pincer backbone of the PDP ligand alleviates the steric congestion around the metal center and allows for introduction of a second equivalent of H_2_^Mes^PDP^Ph^. Protonolysis of the metallacycle by reaction with an N–H functionality of the incoming H_2_^Mes^PDP^Ph^ ligand precursor, recovers the original [^Mes^PDP^Ph^]^2–^ ligand and produces a new Zr–N bond for the second pincer. The critical role of cyclometalation, which is not observed in reactions with ZrCl_4_ or Zr(NMe_2_)_4_, would also explain the unique position of ZrBn_4_ as the only viable starting material for the synthesis of sterically encumbered Zr(^Mes^PDP^Ph^)_2_.

### Synthesis of Photoluminescent Hf(^R1^PDP^Ph^)_2_ from HfBn_4_ (R^1^ = Me or Mes)

**Scheme 6:**
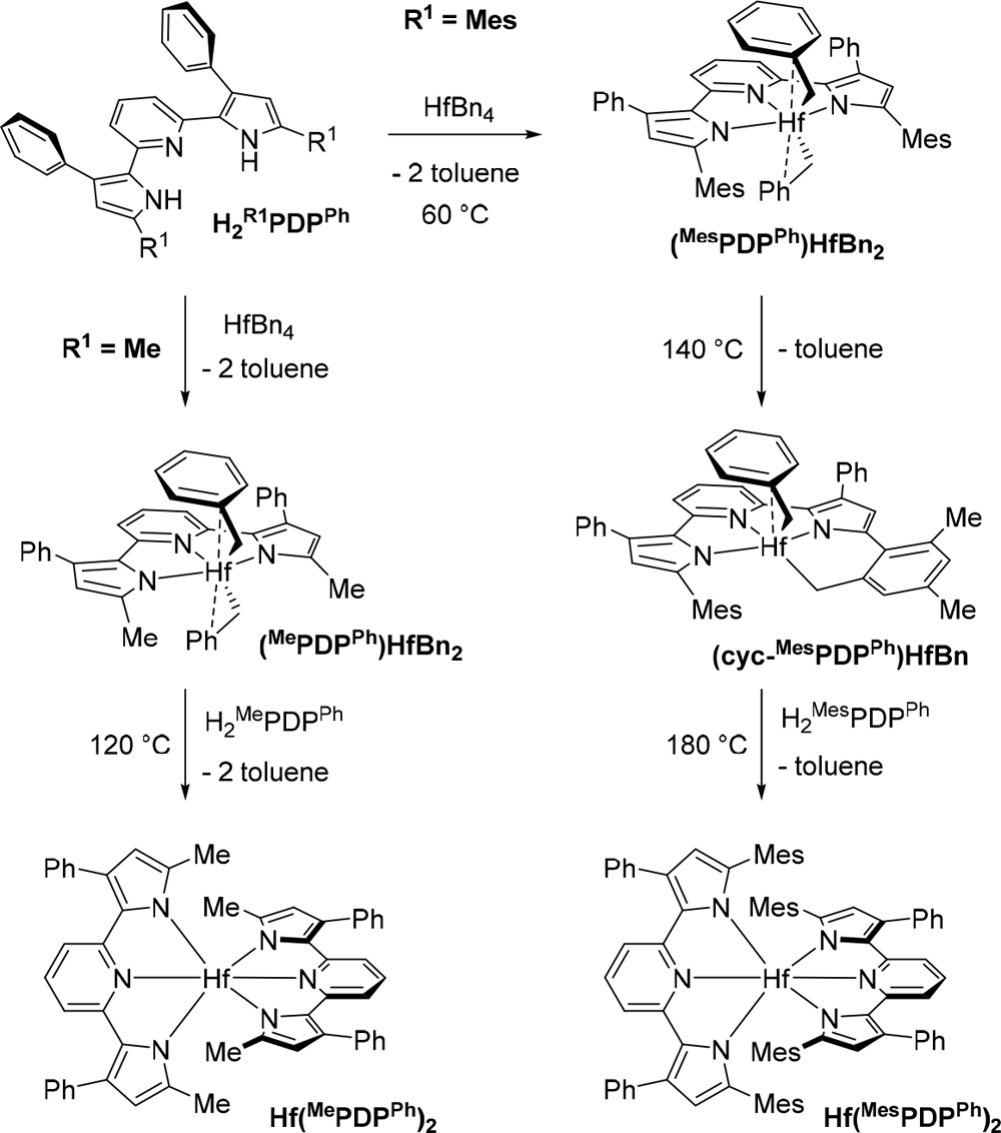
Synthesis of Hafnium Pyridine Dipyrrolide Complexes

**Figure 4:**
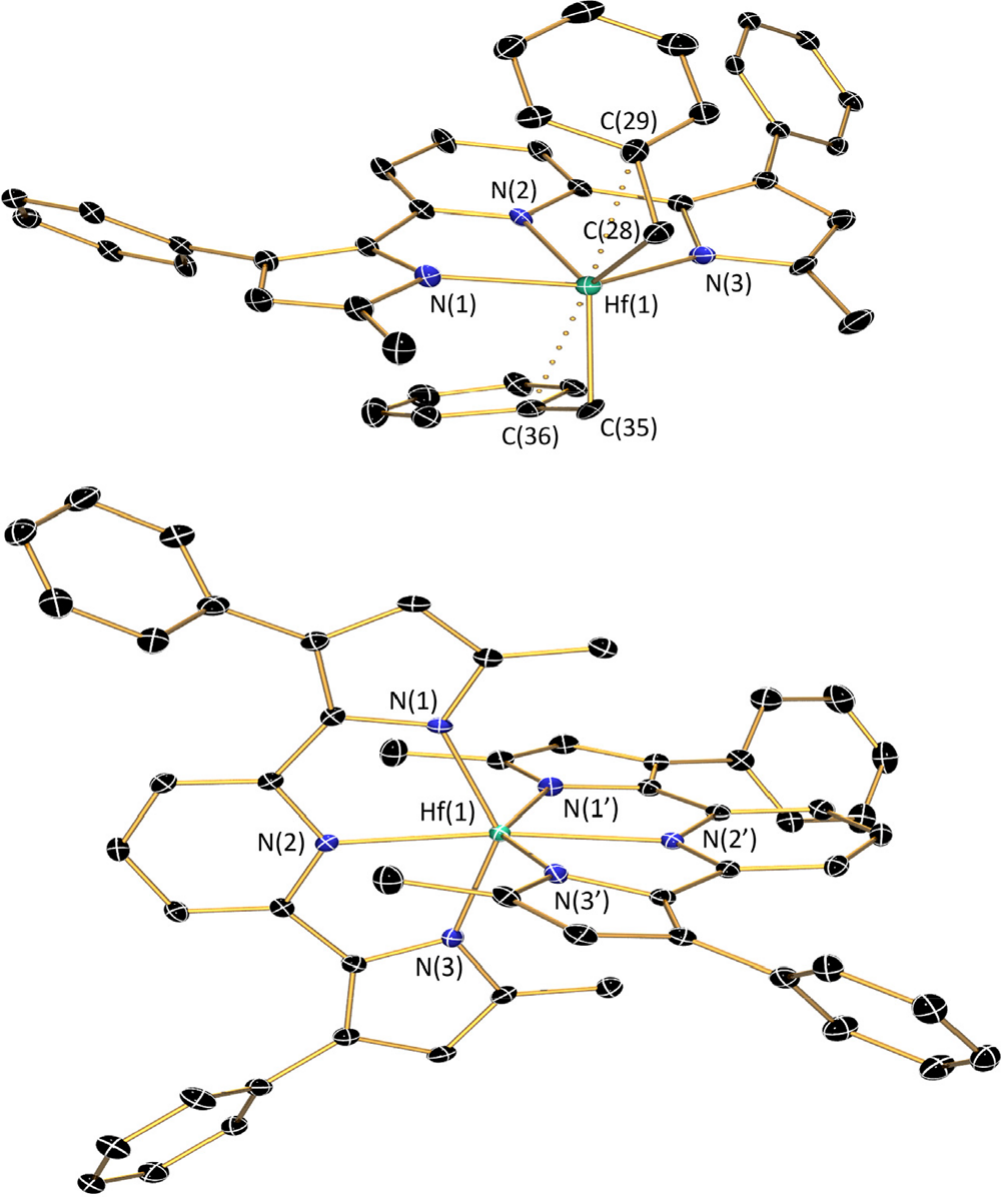
Molecular structures of (^Me^PDP^Ph^)HfBn_2_, top, and Hf(^Me^PDP^Ph^)_2_, bottom, shown with 30% probability ellipsoids. Hydrogen atoms are omitted for clarity.

With a clearer understanding of the formation of Zr(^Me^PDP^Ph^)_2_ and Zr(^Mes^PDP^Ph^)_2_ in mind, we anticipated that the heavier congeners Hf(^Me^PDP^Ph^)_2_ and Hf(^Mes^PDP^Ph^)_2_ might be synthetically accessible under similar reaction conditions starting from HfBn_4_. Reaction between one equivalent of H_2_^Me^PDP^Ph^ and HfBn_4_ at room temperature produced a new species identified by X-ray crystallography and NMR spectroscopy to be (^Me^PDP^Ph^)HfBn_2_ in 66% yield (Scheme 6). Its molecular structure bears a striking resemblance to that of the corresponding zirconium analogue (Figure 4). The five-coordinate hafnium center possesses two η^2^-bound benzyl ligands evidenced by (i) small Hf(1)–C(28)–C(29) and Hf(1)–C(35)–C(36) angles of 93.5(14)° and 91.2(4)° and (ii) short Hf(1)–C(29) and Hf(1)–C(36) distances of 2.693(18) Å and 2.709(5) Å, respectively. Similar to (^Me^PDP^Ph^)ZrBn_2_, disorder of one benzyl group lowers the accuracy in the Hf(1)–C(36) bond length. Further, the metal–nitrogen bonds for the [^Me^PDP^Ph^]^2–^ ligand in (^Me^PDP^Ph^)HfBn_2_ are practically identical with those observed in (^Me^PDP^Ph^)ZrBn_2_, consistent with the very similar effective ionic radii of Hf^IV^ (71 pm) and Zr^IV^ (72 pm).^74,75^ The ^1^H NMR spectrum of (^Me^PDP^Ph^)HfBn_2_ in benzene-*d*_6_ solution is in agreement with the *C*_2v_-symmetric geometry observed in the solid-state structure (Figure S8).

Further in line with the similarities to (^Me^PDP^Ph^)ZrBn_2_, the hafnium bis-benzyl complex is reactive toward a second equivalent of H_2_^Me^PDP^Ph^ only at elevated temperatures, where reaction at 120 °C produced Hf(^Me^PDP^Ph^)_2_ as a red crystalline precipitate in 67% yield (Scheme 6). This species is isostructural to Zr(^Me^PDP^Ph^)_2_, as determined by single-crystal X-ray diffraction (Figure 4) and evidenced by remarkably similar structural metrics (Table 2).

**Table 2:** Comparison of the Metal–Ligand Bond Lengths (Å) in Hafnium Pyridine Dipyrrolide Complexes

	(^Me^PDP^Ph^)HfBn_2_	(cyclo-^Mes^PDP^Ph^)HfBn	Hf(^Me^PDP^Ph^)_2_	Hf(^Mes^PDP^Ph^)_2_
Hf(1)–N(1)	2.143(5)	2.1316(16)	2.145(3)	2.149(2)
				2.158(2)
Hf(1)–N(2)	2.243(4)	2.2624(16)	2.272(3)	2.2376(18)
				2.2339(18)
Hf(1)–N(3)	2.150(5)	2.1141(16)	2.155(3)	2.157(2)
				2.1644(19)
Hf(1)–C(28)/C(41)	2.17(3)	2.219(2)		
Hf(1)–C(35)/C(44)	2.220(7)	2.214(2)		

Akin to the observations for ZrBn_4_, reaction of HfBn_4_ with one equivalent of H_2_^Mes^PDP^Ph^ provided no conversion of starting materials in room-temperature benzene-*d*_6_ solution. Upon heating to 60 °C, cautious monitoring of the reaction mixture by ^1^H NMR spectroscopy revealed consumption of the starting materials and the presence of a new PDP-containing species as the major product. The clear similarities of the ^1^H NMR spectrum (Figure S10) to that of (^Me^PDP^Ph^)HfBn_2_ (i.e., apparent *C*_2v_ symmetry) and the presence of two equivalents of toluene as a byproduct serve as strong evidence for the formation of (^Mes^PDP^Ph^)HfBn_2_ in this reaction (Scheme 6). Unfortunately, all attempts to crystallize this species for X-ray diffraction analysis were unsuccessful, but clean ^1^H and ^13^C NMR spectra were obtained which are fully consistent with the bis-benzyl species. Interestingly, the zirconium analogue to (^Mes^PDP^Ph^)HfBn_2_ has never been observed in our hands, which could be due to the increased reactivity of zirconium-benzyl relative to hafnium-benzyl bonds.

**Figure 5:**
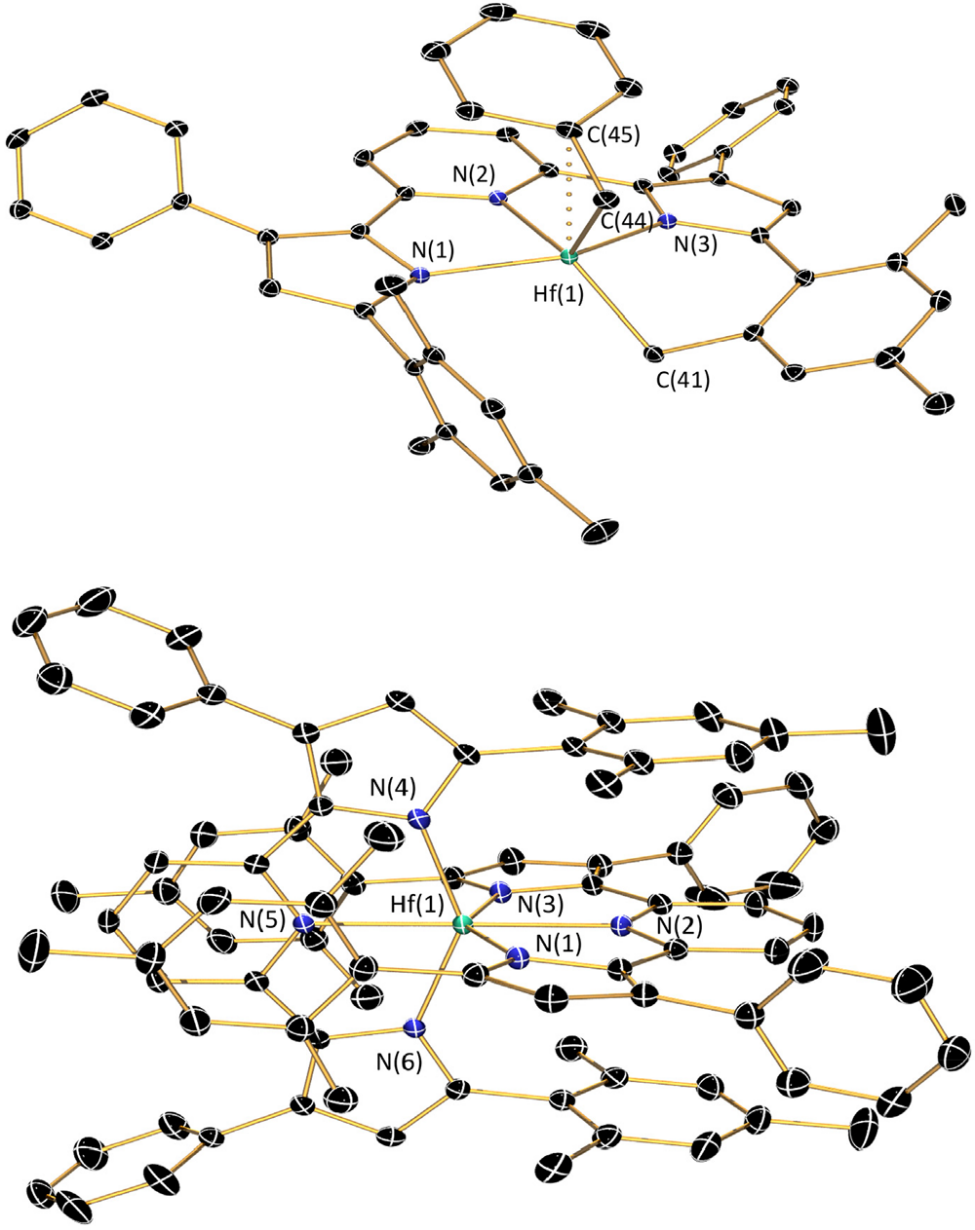
Molecular structures of (cyclo-^Mes^PDP^Ph^)HfBn, top, and Hf(^Mes^PDP^Ph^)_2_, bottom, shown with 30% probability ellipsoids. Hydrogen atoms are omitted for clarity.

Further heating of an equimolar mixture of HfBn_4_ and H_2_^Mes^PDP^Ph^ to 140 °C provided clean conversion to a compound with nearly identical ^1^H NMR signals as (cyclo-^Mes^PDP^Ph^)ZrBn and three equivalents of toluene. A comprehensive suite of correlated NMR spectroscopic techniques on this *in situ* generated sample are fully consistent with formation of *C*_1_-symmetric (cyclo-^Mes^PDP^Ph^)HfBn under these reaction conditions (see Figures S16–S50 for detailed NMR spectroscopic analysis). Crystalline material was isolated in 52% yield from a concentrated solution of the complex in diethyl ether at −35 °C. Single-crystal X-ray diffraction provided conclusive evidence, with a molecular structure nearly identical with that observed for (cyclo-^Mes^PDP^Ph^)ZrBn (Figure 5 and Table 2). Interestingly, reaction of two equivalents of H_2_^Mes^PDP^Ph^ with HfBn_4_ provided no further reactivity at this temperature. However, heating a 2:1 mixture of H_2_^Mes^PDP^Ph^ and HfBn_4_ in mesitylene at 180 °C provided the desired Hf(^Mes^PDP^Ph^)_2_ in 83% yield (Scheme 6), identified by X-ray diffraction and NMR spectroscopy. The molecular structure exhibits nearly perfect *D*_2d_ symmetry (Figure 5), with an N(5)–Hf(1)–N(2) angle of 179.16(8)° and a PDP(1)–PDP(2) dihedral angle of 89.4(3)°. This, combined with similar Hf–N bond lengths in the PDP ligand (Table 2) and close π–π interactions (3.6099(19)–3.7250(19) Å) between the pyridine and mesityl units makes Hf(^Mes^PDP^Ph^)_2_ isostructural to Zr(^Mes^PDP^Ph^)_2_. Like its Zr analogue, Hf(^Mes^PDP^Ph^)_2_ is air- and moisture-stable as a solid and in solution, which can be attributed to the steric protection of the Hf–N bonds by the bulky mesityl substituents.

**Figure 6:**
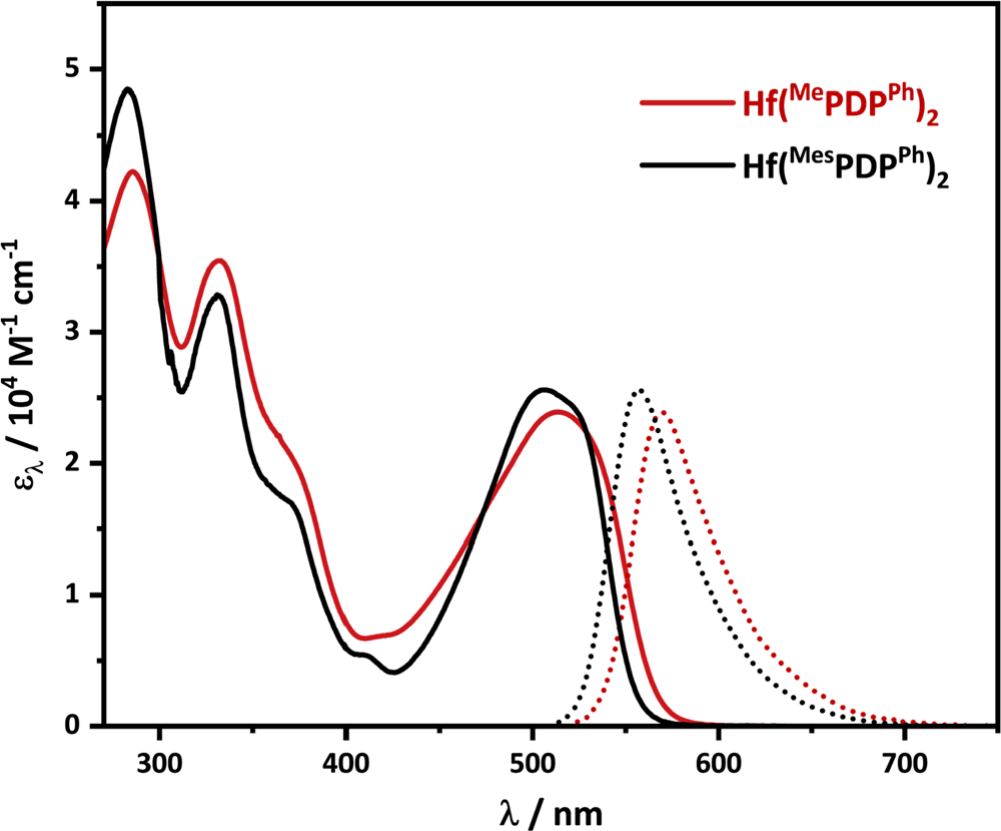
Electronic absorption (solid) and normalized emission (dotted) spectra of Hf(^R1^PDP^Ph^)_2_ (R^1^ = Me or Mes) recorded in room-temperature THF solution.

With the Hf(^R1^PDP^Ph^)_2_ (R^1^ = Me, Mes) complexes in hand, our attention turned toward investigating their photophysical properties. The electronic absorption and emission spectra recorded for both compounds in room-temperature THF solution are shown in Figure 6. Consistent with the red appearance of solid samples, both complexes exhibit strong visible-light absorption bands (ε_λ_ > 20,000 M^–1^ cm^–1^) with Hf(^Me^PDP^Ph^)_2_ (λ_max_ = 514 nm) showing a subtle red-shift compared to Hf(^Mes^PDP^Ph^)_2_ (λ_max_ = 507 nm). Excitation of these lowest-energy absorption bands results in emission maxima at 569 nm for Hf(^Me^PDP^Ph^)_2_ and 558 nm for Hf(^Mes^PDP^Ph^)_2_. Again, a small red-shift is observed for Hf(^Me^PDP^Ph^)_2_. Comparison of the absorption and emission energies in both compounds reveals nearly identical Stokes shifts of 1881 and 1803 cm^–1^ (Table 3). These properties are very similar to those reported for Zr(^Me^PDP^Ph^)_2_ and Zr(^Mes^PDP^Ph^)_2_. The slight blue-shift of the absorption and emission maxima are consistent with the higher-energy d orbitals of hafnium (5d) compared to zirconium (4d) and the resulting higher reduction potential for Hf^IV^. All told, these data suggest a similar LMCT excited-state manifold as reported for our zirconium photosensitizers in the heavier hafnium congeners.

**Table 3:** Comparison of the Photophysical Properties of M(^R1^PDP^Ph^)_2_ (M = Zr, Hf; R^1^ = Me, Mes)

	Hf(^Me^PDP^Ph^)_2_	Hf(^Mes^PDP^Ph^)_2_	Zr(^Me^PDP^Ph^)_2_^a^	Zr(^Mes^PDP^Ph^)_2_^a^
λ_abs_ (nm)	514	507	528	525
λ_em_ (nm)	569	558	595	581
Stokes Shift (cm^–1^)	1881	1803	2133	1836
τ_PL_ (μs)	488	450	325	350
Φ_PL_	0.18	0.41	0.08	0.45

a Values for Zr(^Me^PDP^Ph^)_2_ and Zr(^Mes^PDP^Ph^)2 are taken from references (65, 66), and (67), respectively.

Further photophysical measurements revealed additional subtle differences between the isostructural hafnium and zirconium photosensitizers. Photoluminescence decays measured for Hf(^Me^PDP^Ph^)_2_ and Hf(^Mes^PDP^Ph^)_2_ (Figure S52) revealed lifetimes of 488 and 450 μs at room temperature, respectively, ca. 100–150 μs longer than what is observed for the zirconium analogues (Table 3). Quantum yield determination for both hafnium complexes (Figure S51) revealed quantum efficiencies of 0.18 for Hf(^Me^PDP^Ph^)_2_ and 0.41 for Hf(^Mes^PDP^Ph^)_2_. Despite these minute differences, the parallels in the photoluminescent properties of the new hafnium chromophores compared to their Zr analogues strongly suggest similar excited-state dynamics and emission by thermally activated delayed fluorescence (TADF) rather than phosphorescence. However, more detailed studies that are beyond the scope of this initial report will be required to firmly establish the photophysical properties of Hf(PDP)_2_ complexes and study the effects of the heavy element Hf on intersystem crossing rates as well as radiative and nonradiative decay pathways.

## Conclusions

**Scheme 7:**
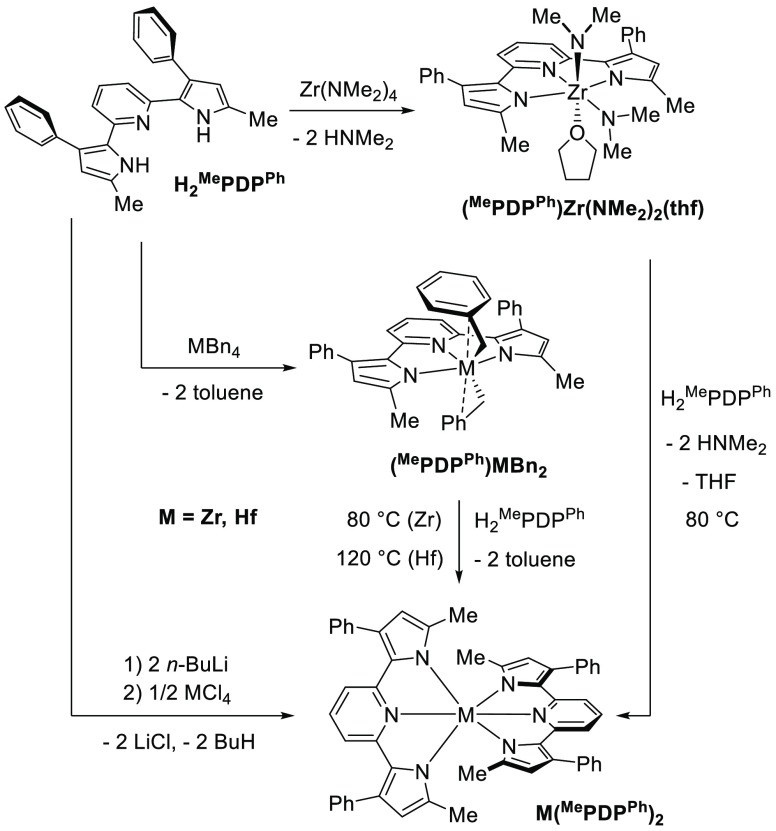
Summary of Reaction Pathways and Intermediates Using the Ligand Precursor H_2_^Me^PDP^Ph^

**Scheme 8:**
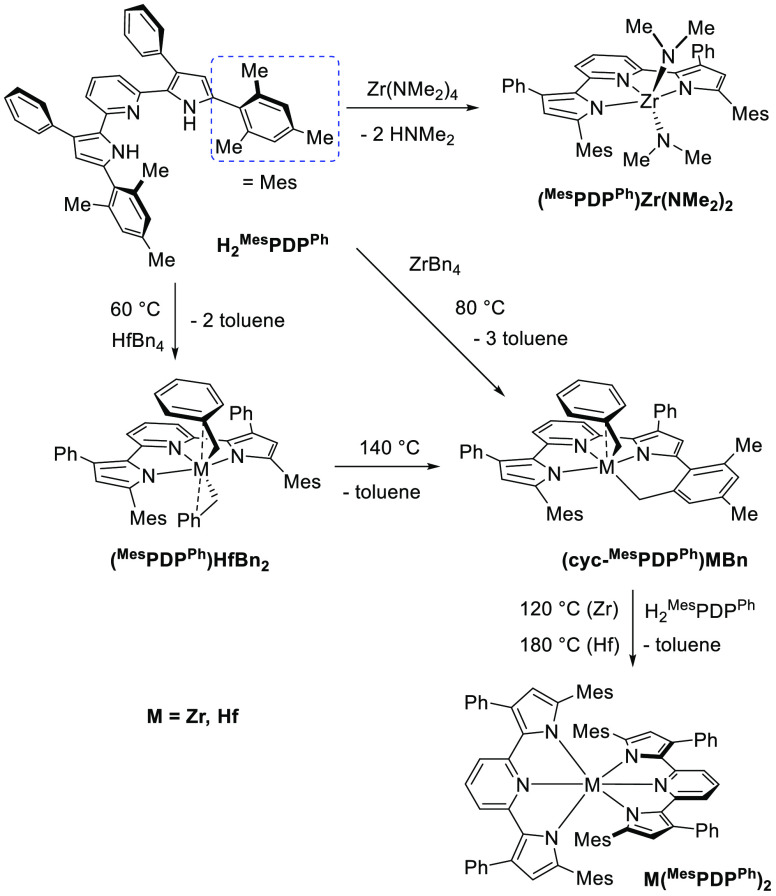
Summary of Reaction Pathways and Intermediates Using the Bulky Ligand Precursor H_2_^Mes^PDP^Ph^

In this study, we have explored different synthetic pathways to photoluminescent complexes of the general formula M(^R1^PDP^Ph^)_2_ (M = Zr, Hf; R^1^ = Me, Mes) and, for the first time, describe emissive hafnium analogues to our previously reported zirconium photosensitizers. During this process, we established a small library of new pyridine dipyrrolide-supported zirconium and hafnium complexes that can serve as precursors for the synthesis of the group 4 chromophores M(^R1^PDP^Ph^)_2_ (M = Zr, Hf; R^1^ = Me, Mes) or are observable as intermediates during previously established synthetic procedures (Scheme 7 and Scheme 8).

While the bis-amide complex (^Me^PDP^Ph^)Zr(NMe_2_)_2_(thf) is capable of reaction with a second H_2_(^Me^PDP^Ph^) ligand to form the bis-PDP complex Zr(^Me^PDP^Ph^)_2_, the sterically more encumbered (^Mes^PDP^Ph^)Zr(NMe_2_)_2_ is incapable of producing the homoleptic Zr(^Mes^PDP^Ph^)_2_. In this manner, tetrabenzylzirconium holds a privileged role as a starting material, as reaction between ZrBn_4_ and two equivalents of ligand produces the respective Zr(^R1^PDP^Ph^)_2_ (R^1^ = Me or Mes) complexes at elevated temperatures in both cases. Monitoring the synthesis of Zr(^Mes^PDP^Ph^)_2_ by ^1^H NMR spectroscopy revealed the presence of the *C*_1_-symmetric, cyclometalated (cyclo-^Mes^PDP^Ph^)ZrBn during the reaction. Structural determination by X-ray crystallography elucidated the importance of cyclometalation in opening the metal’s coordination sphere for the approach of a second bulky H_2_(^Mes^PDP^Ph^) ligand, explaining the lack of reactivity for (^Mes^PDP^Ph^)Zr(NMe_2_)_2_. Beyond providing a more detailed understanding of the formation of Zr(^R1^PDP^Ph^)_2_ complexes (R^1^ = Me, Mes), our studies also raise the interesting question of whether heteroleptic complexes of the general formula Zr(^R1^PDP^R2^)(^R3^PDP^R4^), containing sterically and electronically distinct PDP ligands, could be synthetically accessible. Efforts toward such species, which may exhibit unique photophysical and photochemical properties, are currently underway.

The thorough understanding of the synthetic pathways for zirconium were further leveraged for the preparation of the hafnium bis-PDP molecules Hf(^R1^PDP^Ph^)_2_ (R^1^ = Me or Mes) from tetrabenzylhafnium, with the isolation and characterization of important (^R1^PDP^Ph^)HfBn_2_ (R^1^ = Me or Mes) and (cyclo-^Mes^PDP^Ph^)HfBn intermediates. Both Hf(^Me^PDP^Ph^)_2_ and Hf(^Mes^PDP^Ph^)_2_ were found to be photoluminescent following visible light excitation, with quantum efficiencies of 18% and 41% and excited-state lifetimes of 488 and 450 μs, respectively. These results expand our approach of using d^0^ metal centers for the design of photoluminescent compounds beyond zirconium and direct future interest toward investigating a more substantial change in the identity of the coordinated d^0^ transition metal, particularly those in group 3 or 5.

## Experimental Details

### General Considerations

All air- and moisture-sensitive manipulations were carried out using standard Schlenk line and cannula techniques or in an MBraun inert atmosphere drybox containing an atmosphere of purified nitrogen. Solvents for air- and moisture-sensitive manipulations were dried and deoxygenated using a Glass Contour Solvent Purification System and stored over 4 Å molecular sieves. All solids were dried under a high vacuum, all liquids were deoxygenated prior to bringing them into the glovebox. Deuterated benzene (C_6_D_6_) for NMR measurements was distilled from sodium metal. Tetrabenzylzirconium,^73^ tetrabenzylhafnium,^76^ H_2_^Me^PDP^Ph^,^65^ and H_2_^Mes^PDP^Ph ^^77^ were synthesized according to literature reports. All other chemicals were purchased from commercial sources and used as received.

### Physical Measurements

^1^H and ^13^C{^1^H} NMR spectra were recorded on either an Agilent 400 MHz or Varian INOVA 600 MHz spectrometer. All chemical shifts are reported relative to SiMe_4_ using ^1^H (residual) chemical shifts of the solvent as a secondary standard. Electronic absorption spectra were recorded using a Shimadzu UV-1800 spectrophotometer in gastight quartz cuvettes with a 10 mm path length fitted with J. Young valves. Steady-state emission spectra were obtained in 10 mm path length gastight quartz cuvettes with J. Young valves using a Shimadzu RF-5301 PC spectrofluorophotometer. Time-resolved emission data were collected using a Horiba Jobin Yvon Fluorolog-3 Spectrofluorometer equipped with a single photon counting module in multichannel scaler mode and a 516 nm spectraLED pulsed excitation light source. Emission lifetimes were determined using the provided decay analysis software package, DAS v6.1. Elemental analyses were performed at Robertson Microlit Laboratories, Inc., in Ledgewood, NJ.

### X-ray Crystallography

Single crystals suitable for X-ray diffraction were coated with polyisobutylene oil (Sigma-Aldrich) in a drybox, mounted on a nylon loop, and then quickly transferred to the goniometer head of a Bruker AXS D8 Venture fixed-chi X-ray diffractometer equipped with a Triumph monochromator, a Mo Kα radiation source (λ = 0.71073 Å), and a PHOTON 100 CMOS detector. The samples were cooled to 100 K with an Oxford Cryostream 700 system and optically aligned. The APEX3 software program (version 2016.9–0) was used for diffractometer control, preliminary frame scans, indexing, orientation matrix calculations, least-squares refinement of cell parameters, and the data collection. Three sets of 12 frames each were collected using the omega scan method with a 10 s exposure time. Integration of these frames followed by reflection indexing and least-squares refinement produced a crystal orientation matrix for the crystal lattice that was used for the structural analysis. The data collection strategy was optimized for completeness and redundancy using the Bruker COSMO software suite. The space group was identified, and the data were processed using the Bruker SAINT+ program and corrected for absorption using SADABS. The structures were solved using direct methods (SHELXS) completed by subsequent Fourier synthesis and refined by full-matrix least-squares procedures using the programs provided by SHELXL-2014.

### Comment on Elemental Analysis

Despite repeated attempts, the collection of satisfactory elemental analysis results proved to be problematic for several complexes prepared in this study. The values given below for each compound reflect typical data obtained by elemental analysis. As for their zirconium congeners, the bis-ligand complexes Hf(^Me^PDP^Ph^)_2_ and Hf(^Mes^PDP^Ph^)_2_ gave acceptable values for C, H, and N from combustion analysis if a combustion aid was used. In contrast, all monopincer compounds provided consistently low carbon values, which we attribute to the formation of exceedingly stable zirconium carbide or hafnium carbide even under ideal combustion conditions.^78^ Notably, both group IV carbides are ultrahigh temperature ceramics that are stable at extreme temperatures exceeding 2,000 °C and exhibit excellent oxidation resistance.^79^ No attempt at elemental analysis was made for the complexes (cyclo-^Mes^PDP^Ph^)HfBn and (cyclo-^Mes^PDP^Ph^)ZrBn due to their high light- and temperature-sensitivity.

### Preparation of Zr(^Me^PDP^Ph^)(NMe_2_)_2_(thf)

In the glovebox, a 20 mL scintillation vial was charged with Zr(NMe_2_)_4_ (103 mg, 0.39 mmol, 1 equiv) and dissolved in approximately 8 mL of benzene. H_2_^Me^PDP^Ph^ (150 mg, 0.39 mmol, 1 equiv) was then added as a solid and the walls of the vial were rinsed with an additional 1 mL of benzene. The resulting dark red-brown solution was stirred for 16 h and then evaporated to dryness to yield a dark brown powder. The solid was redissolved in a minimum volume of THF and filtered through a glass microfiber filter into another 20 mL scintillation vial. The concentrated THF solution was layered with pentane and placed into a −35 °C freezer. The desired compound crystallized as large, brown-yellow plates (115 mg, 47% yield) which were suitable for X-ray diffraction. ^1^H NMR (400 MHz, C_6_D_6_, 25 °C, ppm) δ 7.65 (d, 4H, ^3^*J* = 6.8 Hz), 7.22 (t, 4H, ^3^*J* = 7.4 Hz), 7.11 (t, 2H, ^3^*J* = 7.4 Hz), 6.99 (d, 2H, ^3^*J* = 8.0 Hz), 6.53 (t, 1H, ^3^*J* = 8.0 Hz), 6.29 (s, 2H), 3.32 (m, 4H), 2.93 (s, 12H), 2.52 (s, 6H), 1.10 (m, 4H). ^13^C{^1^H} NMR (151 MHz, C_6_D_6_, 25 °C, ppm) δ 154.8, 141.0, 139.8, 139.10, 134.0, 130.3, 130.0, 128.7, 126.7, 114.0, 111.7, 69.2, 42.0, 25.4, 15.8. Anal. Calcd for C_35_H_41_N_5_OZr·C_5_H_12_: C, 67.56; H, 7.51; N, 9.85. Found: C, 62.81; H, 6.15; N, 9.48.

### Preparation of Zr(^Mes^PDP^Ph^)(NMe_2_)_2_

In the glovebox, a 20 mL scintillation vial was charged with Zr(NMe_2_)_4_ (67 mg, 0.25 mmol, 1 equiv) and dissolved in approximately 8 mL of toluene. H_2_^Mes^PDP^Ph^ (150 mg, 0.25 mmol, 1 equiv) was then added as a solid and the walls of the vial were rinsed with an additional 1 mL of toluene. The resulting dark red solution was stirred for 16 h and then evaporated to dryness to yield a red-brown powder. The solid was redissolved in a minimum volume of toluene and filtered through a glass microfiber filter into another 20 mL scintillation vial. The concentrated toluene solution was layered with pentane and placed into a −35 °C freezer. The desired compound crystallized as large, red-brown needles (145 mg, 74% yield). ^1^H NMR (600 MHz, C_6_D_6_, 25 °C, ppm) δ 7.68 (d, 4H, ^3^*J* = 7.9 Hz), 7.22 (t, 4H, ^3^*J* = 7.6 Hz), 7.14 (d, 2H, ^3^*J* = 8.1 Hz), 7.12 (t, 2H, ^3^*J* = 7.6 Hz), 6.79 (s, 4H), 6.60 (t, 1H, ^3^*J* = 8.1 Hz), 6.26 (s, 2H), 2.41 (s, 12H), 2.15 (s, 12H), 2.13 (s, 6H). ^13^C{^1^H} NMR (101 MHz, C_6_D_6_, 25 °C, ppm) δ 155.7, 144.9, 140.8, 139.1, 138.8, 136.6, 134.7, 133.9, 130.8, 130.1, 128.7, 128.3, 128.1, 127.8, 127.7, 126.7, 115.7, 112.0, 38.5, 21.2, 21.1. Anal. Calcd for C_47_H_49_N_5_Zr·C_6_H_6_: C, 74.60; H, 6.50; N, 8.21. Found: C, 71.96; H, 6.43; N, 8.02.

### Preparation of Zr(^Me^PDP^Ph^)Bn_2_

In the glovebox, a 20 mL scintillation vial was charged with ZrBn_4_ (176 mg, 0.39 mmol, 1 equiv), dissolved in approximately 5 mL of benzene, and protected from light using aluminum foil. H_2_^Me^PDP^Ph^ (150 mg, 0.39 mmol, 1 equiv) was then added as a solid and the walls of the vial were rinsed with 1 mL of benzene. The resulting dark red-brown solution was stirred for 16 h and then evaporated to dryness to yield a red-brown powder. The solid was redissolved in a minimum volume of toluene and filtered through a glass microfiber filter into another 20 mL scintillation vial. The concentrated toluene solution was layered with pentane and placed into a −35 °C freezer. The desired compound crystallized as large, red-orange needles (150 mg, 59% yield) which were suitable for X-ray diffraction. ^1^H NMR (600 MHz, C_6_D_6_, 25 °C, ppm) δ 7.53 (t, 4H, ^3^*J* = 7.6 Hz), 7.24 (t, 4H, ^3^*J* = 7.6 Hz), 7.12 (t, 2H, ^3^*J* = 7.4 Hz), 6.71 (t, 4H, ^3^*J* = 7.6 Hz), 6.65 (d, 2H, ^3^*J* = 7.9 Hz), 6.58 (d, 4H, ^3^*J* = 7.3 Hz), 6.47 (t, 2H, ^3^*J* = 7.3 Hz), 6.32 (t, 1H, ^3^*J* = 7.9 Hz), 6.00 (quart, 2H, ^4^*J* = 0.8 Hz), 3.08 (s, 4H), 2.59 (broad S, 6H). ^13^C{^1^H} NMR (151 MHz, C_6_D_6_, 25 °C, ppm) δ 153.3, 139.0, 138.3, 138.0, 133.4, 133.3, 130.7, 129.7, 129.2, 128.9, 128.7, 127.0, 124.6, 113.7, 112.0, 76.2, 16.4. Anal. Calcd for C_41_H_35_N_3_Zr: C, 74.50; H, 5.34; N, 6.36. Found: C, 68.97; H, 5.03; N, 6.13.

### Preparation of (Cyclo-^Mes^PDP^Ph^)ZrBn

In the glovebox, a J. Young tube was charged with H_2_^Me^PDP^Ph^ (50 mg, 0.08 mmol, 1 equiv) and ZrBn_4_ (48 mg, 1.25 equiv) and dissolved in ca. 1 mL of C_6_D_6_. The J. Young tube was removed from the glovebox and heated to 80 °C for 24 h while protected from light. The J. Young tube was then taken back into the glovebox and emptied into a 20 mL scintillation vial. The solvent was removed *in vacuo* and the resulting dark red solid was dissolved in a minimum volume of diethyl ether and filtered into a 20 mL scintillation vial. A small amount of dark red, single-crystalline material suitable for X-ray diffraction was obtained after several days at −35 °C. *All scale-up attempts were unsuccessful.*^1^H NMR (400 MHz, C_6_D_6_, 25 °C, ppm) δ 7.69 (d, 2H, ^3^*J* = 7.3 Hz), 7.65 (d, 2H, ^3^*J* = 7.7 Hz), 7.36 (s, 1H), 7.28 (t, 4H, ^3^*J* = 7.0 Hz), 7.06 (d, 1H, ^3^*J* = 8.1 Hz), 7.02 (d, 1H, ^3^*J* = 7.8 Hz), 6.96 (s, 2H), 6.88 (s, 1H), 6.77 (d, 2H, ^3^*J* = 6.6 Hz), 6.72 (t, 2H, ^3^*J* = 7.7 Hz), 6.54 (s, 1H), 6.54 (t, 1H, ^3^*J* = 8.1 Hz), 6.41 (t, 2H, ^3^*J* = 7.2 Hz), 6.28 (s, 1H), 6.27 (d, 2H, ^3^*J* = 7.5 Hz), 2.77 (d, 1H, ^3^*J* = 10.1 Hz), 2.67 (d, 1H, ^3^*J* = 10.1 Hz), 2.53 (s, 3H), 2.44 (s, 3H), 2.34 (s, 3H), 2.26 (s, 3H), 2.20 (s, 3H), 2.12 (d, 1H, ^3^*J* = 8.3 Hz), 1.86 (d, 1H, ^3^*J* = 8.3 Hz).

### Preparation of Hf(^Me^PDP^Ph^)Bn_2_

The title compound was prepared in a manner analogous to Zr(^Me^PDP^Ph^)Bn_2_ except HfBn_4_ was used. The compound was isolated as red-orange needles (190 mg, 66% yield) which were suitable for X-ray diffraction. ^1^H NMR (600 MHz, C_6_D_6_, 25 °C, ppm) δ 7.53 (d, 4H, ^3^*J* = 6.9 Hz), 7.23 (t, 4H, ^3^*J* = 7.5 Hz), 7.12 (t, 2H, ^3^*J* = 7.4 Hz), 6.72 (t, 4H, ^3^*J* = 7.6 Hz), 6.63 (d, 2H, ^3^*J* = 8.0 Hz), 6.57 (d, 4H, ^3^*J* = 7.2 Hz), 6.50 (t, 2H, ^3^*J* = 7.3 Hz), 6.25 (t, 1H, ^3^*J* = 8.0 Hz), 6.06 (quart, 2H, ^4^*J* = 0.8 Hz), 2.80 (s, 4H), 2.64 (d, 6H, ^4^*J* = 0.8 Hz). ^13^C{^1^H} NMR (151 MHz, C_6_D_6_, 25 °C, ppm) δ 153.2, 139.3, 139.0, 137.9, 134.3, 133.1, 131.0, 130.2, 129.7, 128.7, 128.4, 127.0, 124.6, 114.5, 111.9, 84.1, 16.2. Anal. Calcd for C_41_H_35_N_3_Hf: C, 65.81; H, 4.72; N, 5.62. Found: C, 61.78; H, 4.64; N, 5.40.

### Preparation of Hf(^Mes^PDP^Ph^)Bn_2_

Two J. Young tubes were each charged with H_2_^Mes^PDP^Ph^ (30 mg, 0.10 mmol, 1 equiv) and HfBn_4_ (28 mg, 1.05 equiv) and the solids were dissolved in ca. 0.6 mL C_6_D_6_. The J. Young tubes were covered in aluminum foil, removed from the glovebox, and heated to 60 °C for 24 h. The NMR tubes were brought back into the glovebox, emptied into a 20 mL scintillation vial and the solvent removed *in vacuo* to produce a red-brown powder. The solid was dissolved in a minimum volume of toluene and passed through a glass microfiber filter into another 20 mL scintillation vial. The concentrated toluene solution was then layered with pentane and placed into a −35 °C freezer. The title compound was isolated as an orange microcrystalline material (25 mg, 26% yield). *All scale-up attempts were unsuccessful.*^1^H NMR (600 MHz, C_6_D_6_, 25 °C, ppm) δ 7.56 (d, 4H, ^3^*J* = 7.8 Hz), 7.24 (t, 4H, ^3^*J* = 7.8 Hz), 7.14 (t, 2H, ^3^*J* = 7.4 Hz), 6.98 (t, 4H, ^3^*J* = 7.7 Hz), 6.87 (d, 4H, ^3^*J* = 7.3 Hz), 6.86 (d, 2H, ^3^*J* = 8.1 Hz), 6.76 (t, 2H, ^3^*J* = 7.4 Hz), 6.72 (s, 4H), 6.33 (t, 1H, ^3^*J* = 8.1 Hz), 6.14 (s, 2H), 2.27 (s, 4H), 2.17 (s, 12H), 2.08 (s, 6H). ^13^C{^1^H} NMR (151 MHz, C_6_D_6_, 25 °C, ppm) δ 153.2, 144.7, 139.88, 139.86, 138.4, 137.3, 137.1, 135.0, 132.7, 130.7, 130.3, 129.5, 128.4, 128.3, 128.1, 126.8, 124.2, 116.4, 112.4, 89.2, 21.1, 20.8.

### Preparation of (Cyclo-^Mes^PDP^Ph^)HfBn

Two J. Young tubes were each charged with H_2_^Mes^PDP^Ph^ (30 mg, 0.05 mmol, 1 equiv) and HfBn_4_ (29 mg, 1.05 equiv) and the solids were dissolved in ca. 0.6 mL C_6_D_6_. The J. Young tubes were covered in aluminum foil, removed from the glovebox, and heated to 140 °C for 3 h. The NMR tubes were brought back into the glovebox, emptied into a 20 mL scintillation vial and the solvent removed *in vacuo* to produce a dark red powder. The solid was dissolved in a minimum volume of diethyl ether and passed through a glass microfiber filter into another 20 mL scintillation vial. The concentrated ether solution was placed into a −35 °C freezer for 24 h which produced the product as dark red crystals (45 mg, 52% yield) suitable for X-ray diffraction. ^1^H NMR (600 MHz, C_6_D_6_, 25 °C, ppm) δ 7.78 (s, 1H), 7.66 (dddd, 2H, ^3^*J* = 7.68 Hz, ^4^*J* = 1.97 Hz, ^4^*J* = 1.28 Hz, ^5^*J* = 0.62 Hz), 7.65 (dddd, 2H, ^3^*J* = 7.68 Hz, ^4^*J* = 1.97 Hz, ^4^*J* = 1.28 Hz, ^5^*J* = 0.62 Hz), 7.27 (dddd, 2H, ^3^*J* = 7.68 Hz, ^3^*J* = 7.48 Hz, ^4^*J* = 1.49 Hz, ^5^*J* = 0.62 Hz), 7.27 (dddd, 2H, ^3^*J* = 7.68 Hz, ^3^*J* = 7.48 Hz, ^4^*J* = 1.49 Hz, ^5^*J* = 0.62 Hz), 7.17 (dd, 1H, ^3^*J* = 7.48 Hz, ^4^*J* = 1.28 Hz), 7.17 (dd, 1H, ^3^*J* = 7.48 Hz, ^4^*J* = 1.28 Hz), 7.04 (dd, 1H, ^3^*J* = 7.96 Hz, ^4^*J* = 0.7 Hz), 6.98 (s, 1H), 6.97 (dd, 1H, ^3^*J* = 7.96 Hz, ^4^*J* = 0.7 Hz), 6.88 (s, 1H), 6.77 (t, 2H, ^3^*J* = 7.58 Hz), 6.75 (s, 1H), 6.59 (s, 1H), 6.49 (t, 1H, ^3^*J* = 7.96 Hz), 6.44 (t, 1H, ^3^*J* = 7.48 Hz), 6.41 (d, 2H, ^3^*J* = 7.68 Hz), 6.26 (s, 1H), 2.58 (s, 3H), 2.57 (d, 1H, ^2^*J* = 11.5 Hz), 2.47 (s, 3H), 2.36 (s, 3H), 2.26 (s, 3H), 2.20 (s, 3H), 2.11 (d, 1H, ^2^*J* = 11.5 Hz), (d, 1H, ^2^*J* = 10.9 Hz), 1.68 (d, 1H, ^2^*J* = 10.9 Hz). ^13^C{^1^H} NMR (151 MHz, C_6_D_6_, 25 °C, ppm) δ 154.3, 153.8, 142.6, 142.0, 141.5, 140.1, 139.9, 138.3, 137.6, 136.8, 135.8, 135.4, 135.2, 133.9, 131.8, 131.3, 130.4, 130.3, 129.8, 129.4, 129.1, 129.0, 128.9, 128.8, 128.7, 128.3, 127.1, 124.8, 116.1, 115.2, 112.8, 112.4, 90.6, 79.7, 23.4, 21.5, 21.4, 21.31, 21.25.

### Preparation of Hf(^Me^PDP^Ph^)_2_

HfBn_4_ (192 mg, 0.35 mmol, 0.55 equiv) and H_2_^Me^PDP^Ph^ (250 mg, 0.64 mmol, 1 equiv) were loaded into an oven-dried 50 mL thick-walled glass vessel in the glovebox. Approximately 4 mL of benzene was added *excluding* a stir bar. The thick-walled vessel was sealed with a PTFE screw cap and the yellow-orange solution heated to 120 °C for 16 h. During the reaction, the product formed as large red crystals. After cooling to room temperature, the reaction vessel was brought back into the glovebox and the crystalline material was isolated on a medium porosity frit and washed with two 2 mL portions of benzene and 10 mL of pentane. The product was obtained as red crystals (205 mg, 67% yield). ^1^H NMR (600 MHz, CDCl_3_, 25 °C, ppm) δ 7.43 (d, 4H, ^3^*J* = 6.8 Hz), 7.40 (t, 4H, ^3^*J* = 7.4 Hz), 7.32 (t, 2H, ^3^*J* = 7.2 Hz), 7.16 (t, 1H, ^3^*J* = 8.0 Hz), 6.83 (d, 2H, ^3^*J* = 8.0 Hz), 5.83 (quart., 2H, ^4^*J* = 0.8 Hz), 2.05 (d, 6H, ^4^*J* = 0.8 Hz). ^13^C{^1^H} NMR (151 MHz, CDCl_3_, 25 °C, ppm) δ 155.1, 142.0, 141.9, 136.9, 135.3, 131.4, 129.4, 128.6, 127.1, 114.0, 111.8, 14.6. Anal. Calcd for C_54_H_42_N_6_Hf·C_6_H_6_: C, 69.86; H, 4.69; N, 8.15. Found: C, 69.70; H, 4.95; N, 7.53.

### Preparation of Hf(^Mes^PDP^Ph^)_2_

HfBn_4_ (107 mg, 0.20 mmol, 0.55 equiv) and H_2_^Mes^PDP^Ph^ (214 mg, 0.36 mmol, 1 equiv) were loaded into an oven-dried 50 mL thick-walled glass vessel in the glovebox. Approximately 5 mL of mesitylene was added along with a stir bar. The thick-walled vessel was sealed with a PTFE screw cap and the yellow-orange solution heated to 180 °C for 16 h. After cooling to room temperature, the reaction vessel was opened to air and DCM and ethyl acetate were added under regular benchtop conditions. The red solution was passed through a silica gel plug using ethyl acetate as the eluent. The resulting solution was concentrated on the rotary evaporator, layered with hexanes, and placed into the freezer. The product was obtained as red crystals (203 mg, 83% yield). ^1^H NMR (600 MHz, C_6_D_6_, 25 °C, ppm) δ 7.71 (d, 8H, ^3^*J* = 8.2 Hz), 7.38 (t, 8H, ^3^*J* = 7.6 Hz), 7.21 (t, 4H, ^3^*J* = 7.5 Hz), 6.51 (s, 8H), 6.49 (d, 4H, ^3^*J* = 8.1 Hz), 6.26 (t, 2H, ^3^*J* = 8.1 Hz), 5.98 (s, 4H), 2.07 (s, 24H), 1.86 (s, 12H). ^13^C{^1^H} NMR (101 MHz, C_6_D_6_, 25 °C, ppm) δ 155.0, 143.6, 139.8, 138.6, 138.2, 136.8, 136.3, 132.0, 131.6, 129.8, 128.8, 127.2, 116.1, 112.7, 23.0, 20.9. Anal. Calcd for C_86_H_74_N_6_Hf: C, 75.39; H, 5.44; N, 6.13. Found: C, 75.24; H, 5.55; N, 5.90.

## Supporting Information Available

Additional experimental procedures, spectroscopic and crystallographic data, and computational details (PDF)
