# Supplementary material for: Organometallic Intermediates in the Synthesis of Photoluminescent Zirconium and Hafnium Complexes with Pyridine Dipyrrolide Ligands
Source: Organometallics. 2023 Mar 10;42(11):1220–31. doi: 10.1021/acs.organomet.3c00058 (PMC10266360; doi:10.1021/acs.organomet.3c00058)
Supplement: Supplementary file 1 — om3c00058_si_001.pdf [file om3c00058_si_001.pdf]

Organometallic Intermediates in the Synthesis of Photoluminescent Zirconium and Hafnium Complexes with Pyridine Dipyrrolide Ligands

*Dylan C. Leary, Yu Zhang,[‡] Jose G. Rodriguez, Novruz G. Akhmedov, Jeffrey L. Petersen, Brian S. Dolinar, and Carsten Milsmann**

C. Eugene Bennett Department of Chemistry, West Virginia University, Morgantown, West Virginia, USA.

[‡]Current address: Department of Chemistry, Tufts University, Medford, Massachusetts, USA

camilsmann@mail.wvu.edu

Supporting Information

Contents

1. X-Ray Crystallography S3

2. NMR Characterization.. S7

3. Photophysical Measurements S50

1. X-Ray Crystallography

Table S1. Crystallographic data for ($^{Me}PDP^{Ph}$)Zr(NMe_2)$_2$(thf) and ($^{Mes}PDP^{Ph}$)Zr(NMe_2)$_2$.

	($^{Me}PDP^{Ph}$)Zr(NMe_2)$_2$(thf) · 0.5 C_5H_{12}	($^{Mes}PDP^{Ph}$)Zr(NMe_2)$_2$ · C_6H_6
chem. formula	$C_{37.5}H_{47}N_5OZr$	$C_{53}H_{55}N_5Zr$
cryst size, mm	0.199 × 0.145 × 0.139	0.170 x 0.306 x 0.504
Fw, g mol^{-1}	675.02	853.24
space group	P -1	P -1
a, Å	9.308(2)	8.2112(4)
b, Å	13.554(3)	15.7575(7)
c, Å	15.393(3)	17.5530(8)
α, deg	67.585(4)	98.4831(13)
β, deg	75.909(5)	102.6476(13)
γ, deg	71.359(5)	92.2598(13)
V, Å^3	1684.6(6)	2185.73(18)
Z	2	2
T, K	100(2)	100(2)
ρ calcd, g cm^{-3}	1.331	1.296
reflns collected/2Θ$_{max}$	25232/61.32	37130/60.14
unique reflns/I > 2σ(*I*)	10151/7731	12749/10253
No. of params/restraints	437/696	542/0
λ, Å	0.71073	0.71073
R_1^{a}/goodness of fit^b	0.0509/1.014	0.0416/1.042
wR_2^{c} ($I > 2\sigma(I)$)	0.1002	0.0841
Residual density, e$^-$/Å^{-3}	0.84/-1.03	0.70/-0.71

[a]Observation criterion: $I > 2\sigma(I)$, $R_1 = \sum((||F_O| - |F_C||) / \sum|F_O|$. [b]$GoF = [\sum[w(F_o^2 - F_c^2)^2] / (n-p)]^{1/2}$. [c]$wR_2 = [\sum[w(F_o^2 - F_c^2)^2] / \sum[w(F_o^2)^2]]^{1/2}$

Table S2. Crystallographic data for (^{Me}PDP^{Ph})ZrBn$_2$ and (cyclo-^{Mes}PDP^{Ph})ZrBn.

	(^{Me}PDP^{Ph})ZrBn$_2$ · 0.75 C_7H_8	(cyclo-^{Mes}PDP^{Ph})ZrBn · 2 $C_4H_{10}O$
chem. formula	$C_{46.25}H_{41}N_3Zr$	$C_{58}H_{63}N_3O_2Zr$
cryst size, mm	0.118 × 0.164 × 0.500	0.108 x 0.194 x 0.355
Fw, g mol^{-1}	730.04	925.33
space group	P -1	P -1
a, Å	9.9459(6)	11.8577(6)
b, Å	10.9153(8)	12.2031(6)
c, Å	18.9606(13)	17.3845(9)
α, deg	79.311(2)	79.911(2)
β, deg	76.950(2)	87.589(2)
γ, deg	63.350(2)	78.637(2)
V, Å^3	1783.8(2)	2428.0(2)
Z	2	2
T, K	100(2)	100(2)
ρ calcd, g cm^{-3}	1.359	1.266
reflns collected/2Θ_{max}	56973/56.69	41649/50.00
unique reflns/I > 2σ(I)	56973/50495	8499/6910
No. of params/restraints	666/432	586/0
λ, Å	0.71073	0.71073
R_1[a]/goodness of fit[b]	0.0546/1.021	0.0503/1.112
wR_2[c] (I > 2σ(I))	0.1249	0.0871
Residual density, e$^-$/Å^{-3}	1.48/-0.98	0.59/-0.68

[a]Observation criterion: $I > 2\sigma(I)$, $R_1 = \sum((||F_o| - |F_c||) / \sum|F_o|$. [b]GoF = $[\sum[w(F_o^2 - F_c^2)^2] / (n-p)]^{1/2}$. [c]$wR_2 = [\sum[w(F_o^2 - F_c^2)^2] / \sum[w(F_o^2)^2]]^{1/2}$

Table S3. Crystallographic data for (^{Me}PDP^{Ph})HfBn$_2$ and (cyclo-^{Mes}PDP^{Ph})HfBn.

	(^{Me}PDP^{Ph})HfBn$_2$ · 0.75 C_7H_8	(cyclo-^{Mes}PDP^{Ph})HfBn · 2 $C_4H_{10}O$
chem. formula	$C_{46.25}H_{41}N_3Hf$	$C_{58}H_{63}N_3O_2Zr$
cryst size, mm	0.174 × 0.191 × 0.619	0.110 x 0.176 x 0.377
Fw, g mol^{-1}	817.31	1012.60
space group	P -1	P -1
a, Å	9.9534(4)	11.8618(2)
b, Å	10.9224(5)	12.1914(2)
c, Å	18.9075(8)	17.4248(3)
α, deg	79.275(2)	79.8540(10)
β, deg	77.0720(10)	87.5350(10)
γ, deg	63.4560(10)	78.6090(10)
V, Å^3	1783.48(13)	2431.48(7)
Z	2	2
T, K	100(2)	100(2)
ρ calcd, g cm^{-3}	1.522	1.383
reflns collected/2Θ$_{max}$	67864/61.08	120483/55.00
unique reflns/I > 2σ(*I*)	67864/66020	11162/10577
No. of params/restraints	545/255	574/7
λ, Å	0.71073	0.71073
R_1[a]/goodness of fit[b]	0.0391/1.030	0.0208/1.100
wR_2[c] ($I > 2\sigma(I)$)	0.1016	0.0462
Residual density, e$^-$/Å^{-3}	1.82/-2.08	0.71/-0.66

[a]Observation criterion: $I > 2\sigma(I)$, $R_1 = \sum((||F_o| - |F_c||) / \sum|F_o|$. [b]GoF = $[\sum[w(F_o^2 - F_c^2)^2] / (n-p)]^{1/2}$. [c]$wR_2 = [\sum[w(F_o^2 - F_c^2)^2] / \sum[w(F_o^2)^2]]^{1/2}$

Table S4. Crystallographic data for $Hf(^{Me}PDP^{Ph})_2$ and $Hf(^{Mes}PDP^{Ph})_2$.

	$Hf(^{Me}PDP^{Ph})_2 \cdot 2\ C_4H_8O$	$Hf(^{Mes}PDP^{Ph})_2 \cdot C_6H_{12}$
chem. formula	$C_{62}H_{58}HfN_6O_2$	$C_{92}H_{88}HfN_6$
cryst size, mm	0.077 × 0.086 × 0.309	0.209 x 0.167 x 0.136
Fw, g mol^{-1}	1097.63	1456.17
space group	C 2/c	P -1
a, Å	26.5712(10)	16.1230(8)
b, Å	12.9346(4)	16.1452(8)
c, Å	15.5893(6)	16.2592(8)
α, deg	90	90.597(2)
β, deg	105.8416(12)	111.429(2)
γ, deg	90	111.464(2)
V, Å^3	5154.4(3)	3615.5(3)
Z	4	2
T, K	100(2)	100(2)
ρ calcd, g cm^{-3}	1.414	1.338
reflns collected/$2\Theta_{max}$	23700/55.08	131384/60.29
unique reflns/I > 2σ(I)	5926/5157	21215/15637
No. of params/restraints	298/5	939/156
λ, Å	0.71073	0.71073
R_1^a/goodness of fit^b	0.0382/0.987	0.0390/1.044
wR_2^c ($I > 2\sigma(I)$)	0.0919	0.0645
Residual density, e$^-$/Å^{-3}	1.19/-1.46	1.61/-1.74

^{a}Observation criterion: $I > 2\sigma(I)$, $R_1 = \sum((||F_o| - |F_c||) / \sum|F_o|$. ^{b}GoF = $[\sum[w(F_o^2 - F_c^2)^2] / (n-p)]^{1/2}$. ^{c}$wR_2 = [\sum[w(F_o^2 - F_c^2)^2] / \sum[w(F_o^2)^2]]^{1/2}$

2. NMR Characterization

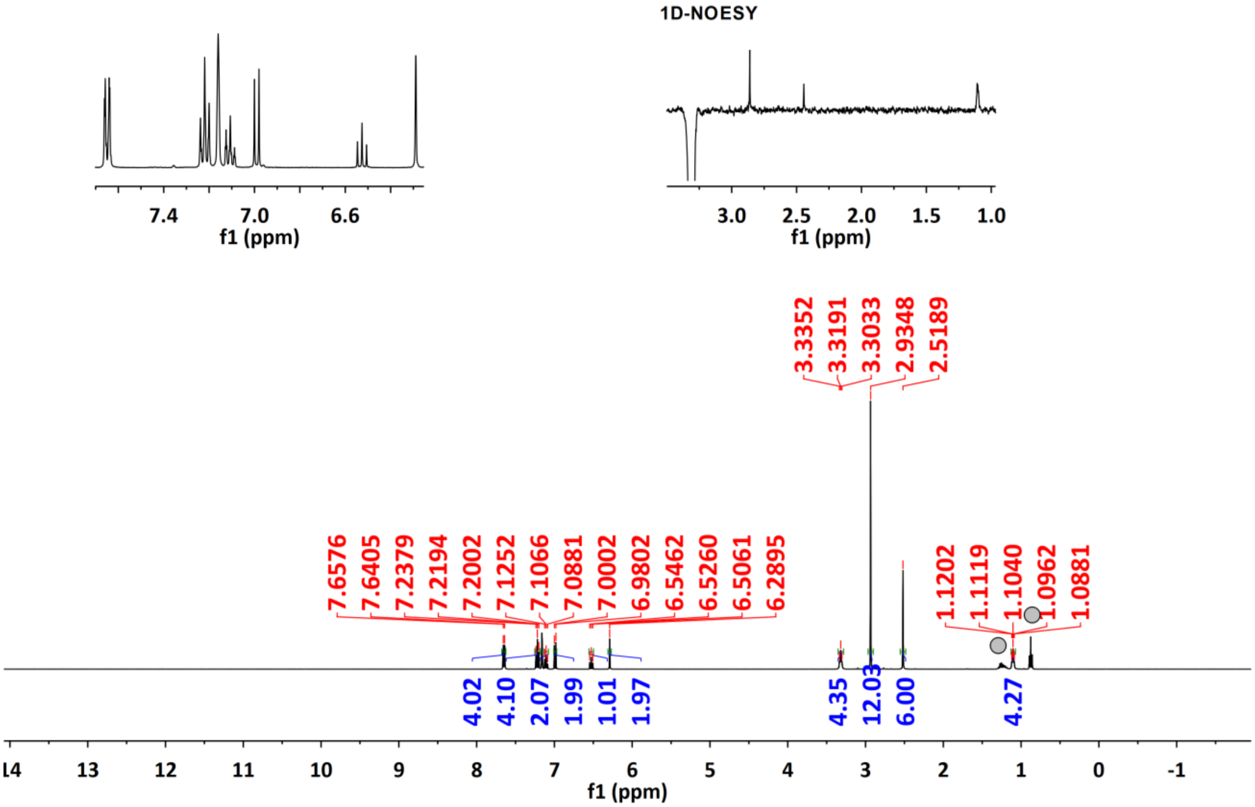

Figure S1. 400 MHz ^1H NMR spectrum of $Zr(^{Me}PDP^{Ph})(NMe_2)_2(thf)$ collected in C_6D_6. Residual pentane signals are labelled with grey circles.

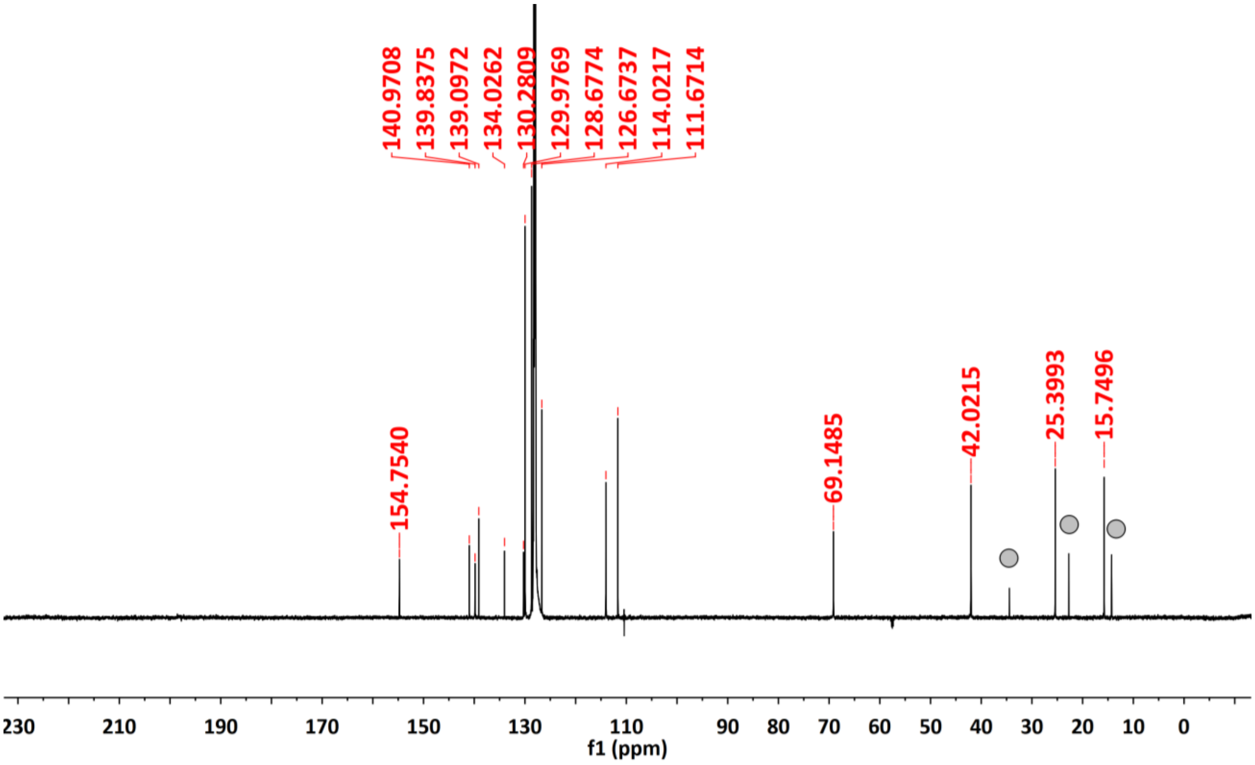

Figure S2. 151 MHz ^{13}C {^{1}H} NMR spectrum of Zr(^{Me}PDP^{Ph})(NMe_2)$_2$(thf) collected in C_6D_6. An instrument artifact is evident at 110.43 ppm. Residual pentane signals are marked with grey circles.

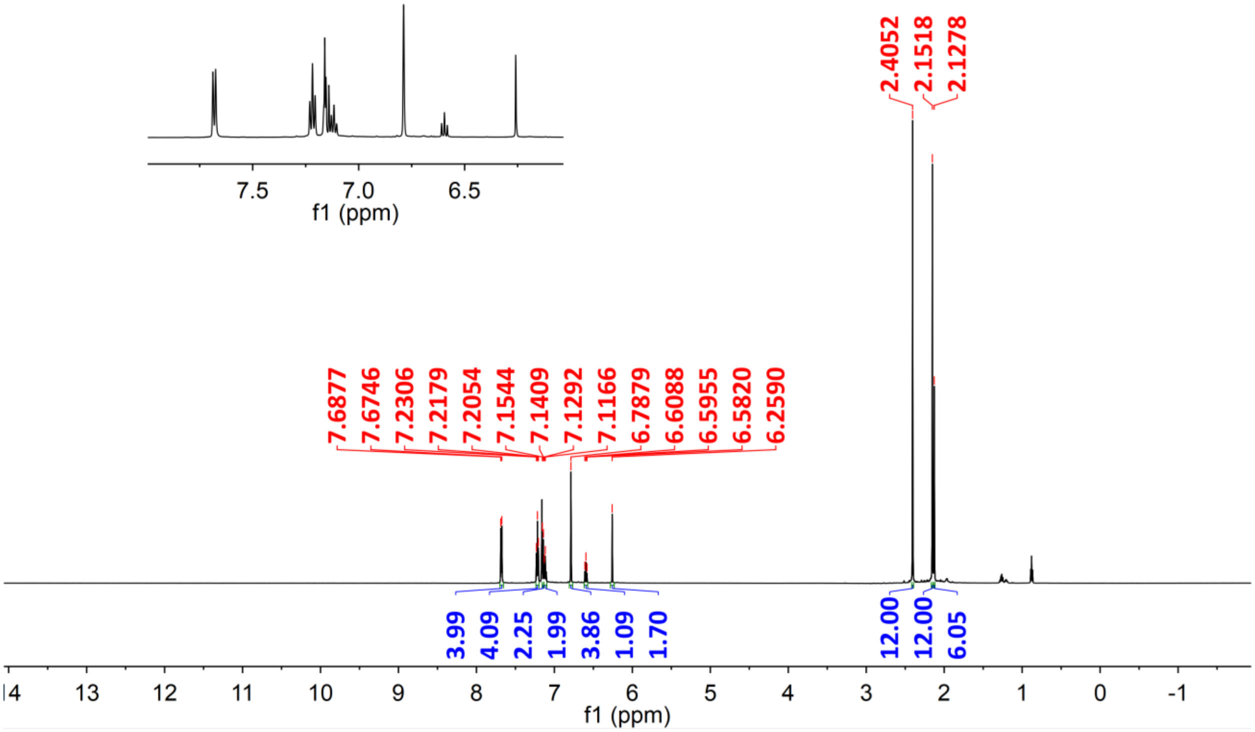

Figure S3. 600 MHz ^{1}H NMR spectrum of Zr(^{Mes}PDP^{Ph})(NMe_2)$_2$ collected in C_6D_6.

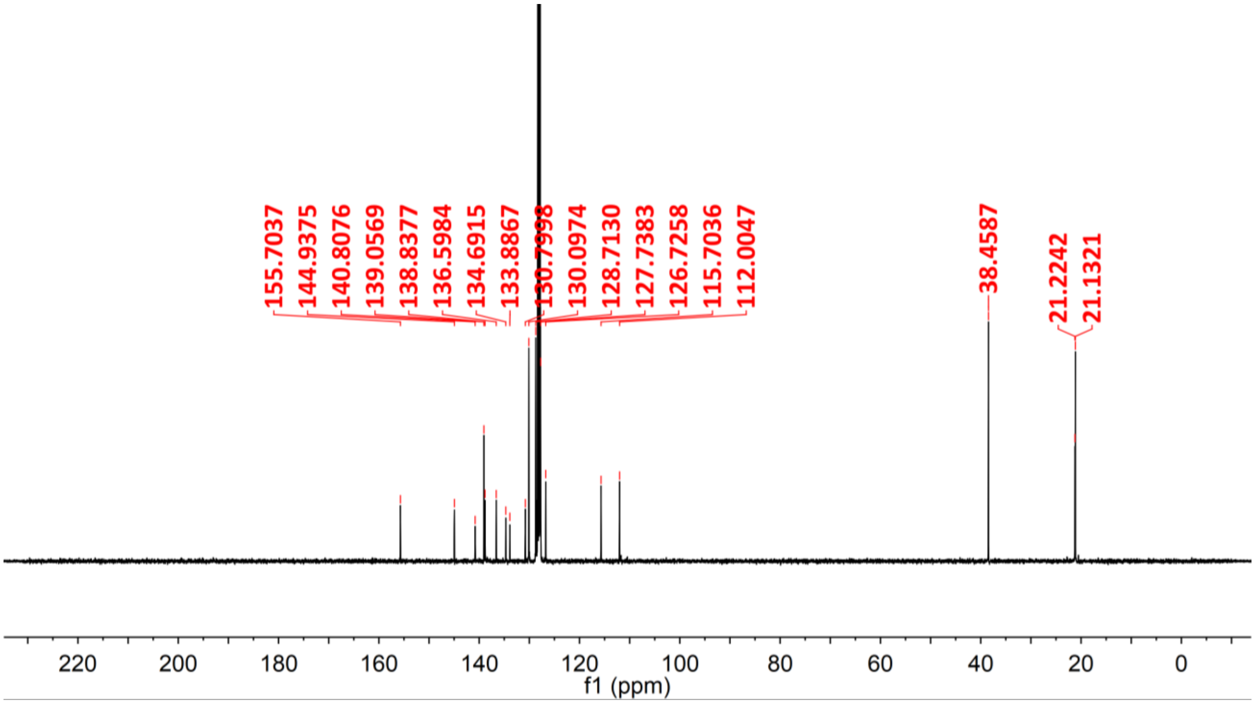

Figure S4. 101 MHz ^{13}C {^{1}H} NMR spectrum of Zr(^{Mes}PDP^{Ph})(NMe_2)$_2$ collected in C_6D_6.

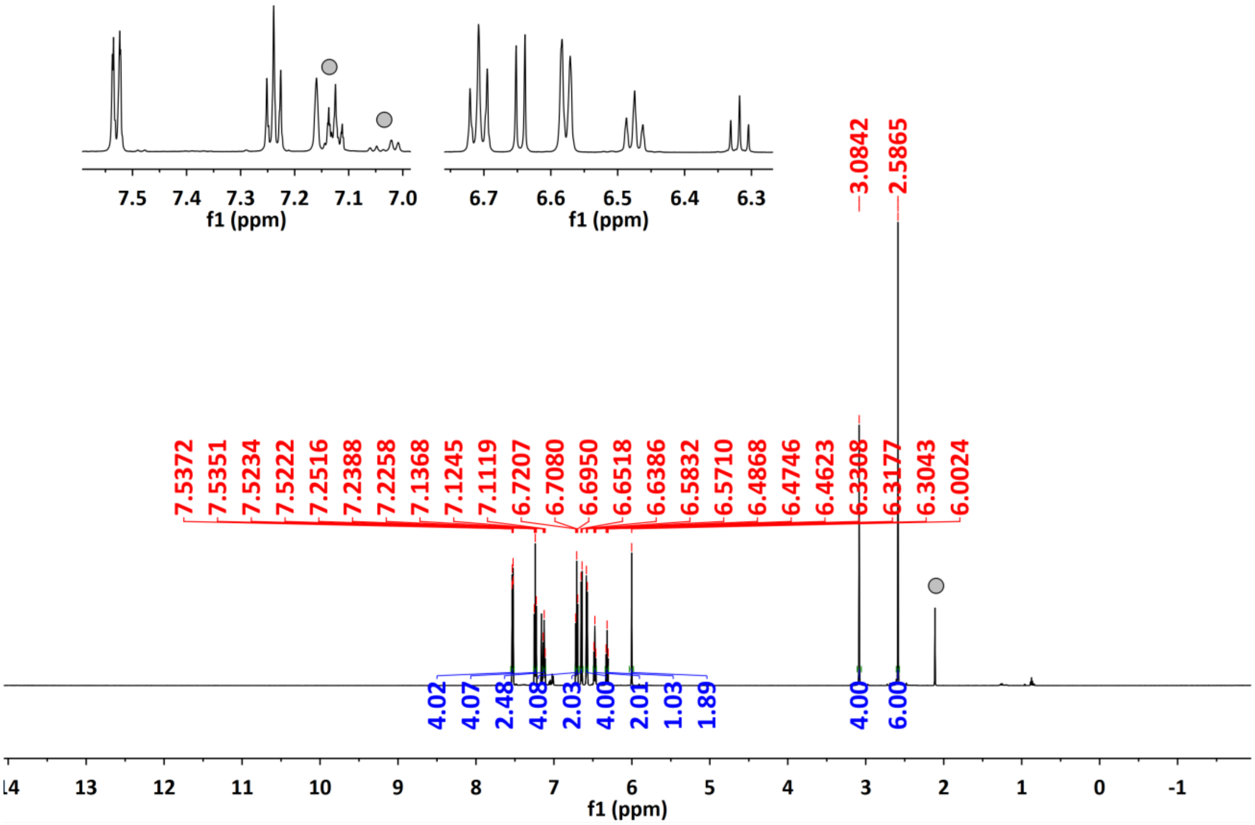

Figure S5. 600 MHz ^{1}H NMR spectrum of Zr(^{Me}PDP^{Ph})Bn_2 collected in C_6D_6. Residual toluene signals are labelled with grey circles.

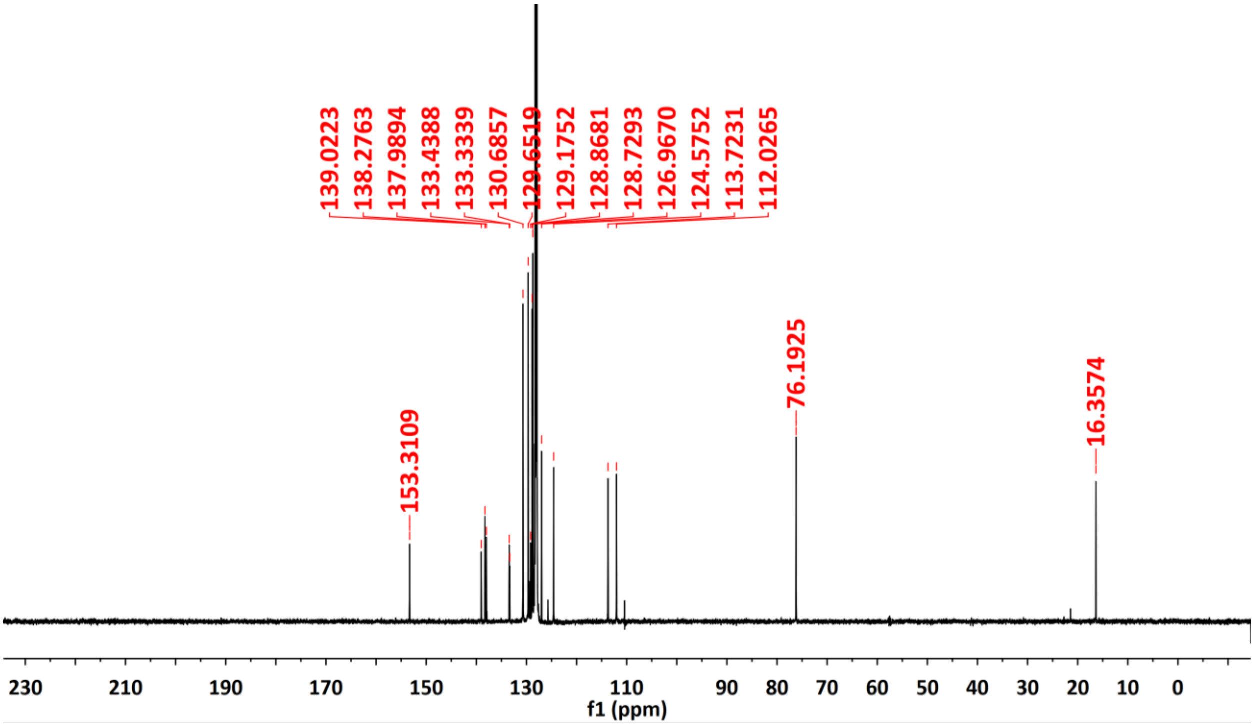

Figure S6. 151 MHz ^{13}C {^{1}H} NMR spectrum of Zr(^{Me}PDP^{Ph})Bn_2 collected in C_6D_6. An instrument artifact is evident at 110.43 ppm. Residual toluene signals are present as minor signals.

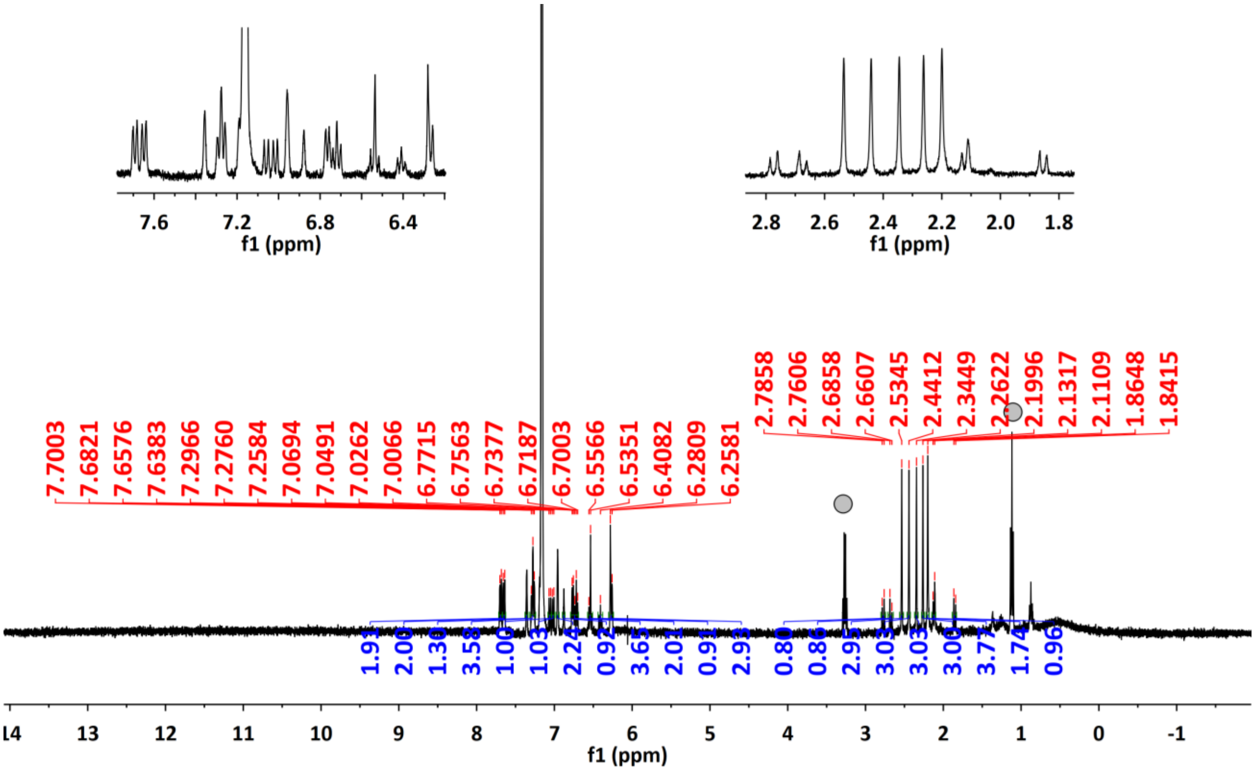

Figure S7. 400 MHz ^{1}H NMR spectrum of Zr(cyclo-^{Mes}PDP^{Ph})Bn collected in C_6D_6. Residual diethyl ether signals are labelled with grey circles.

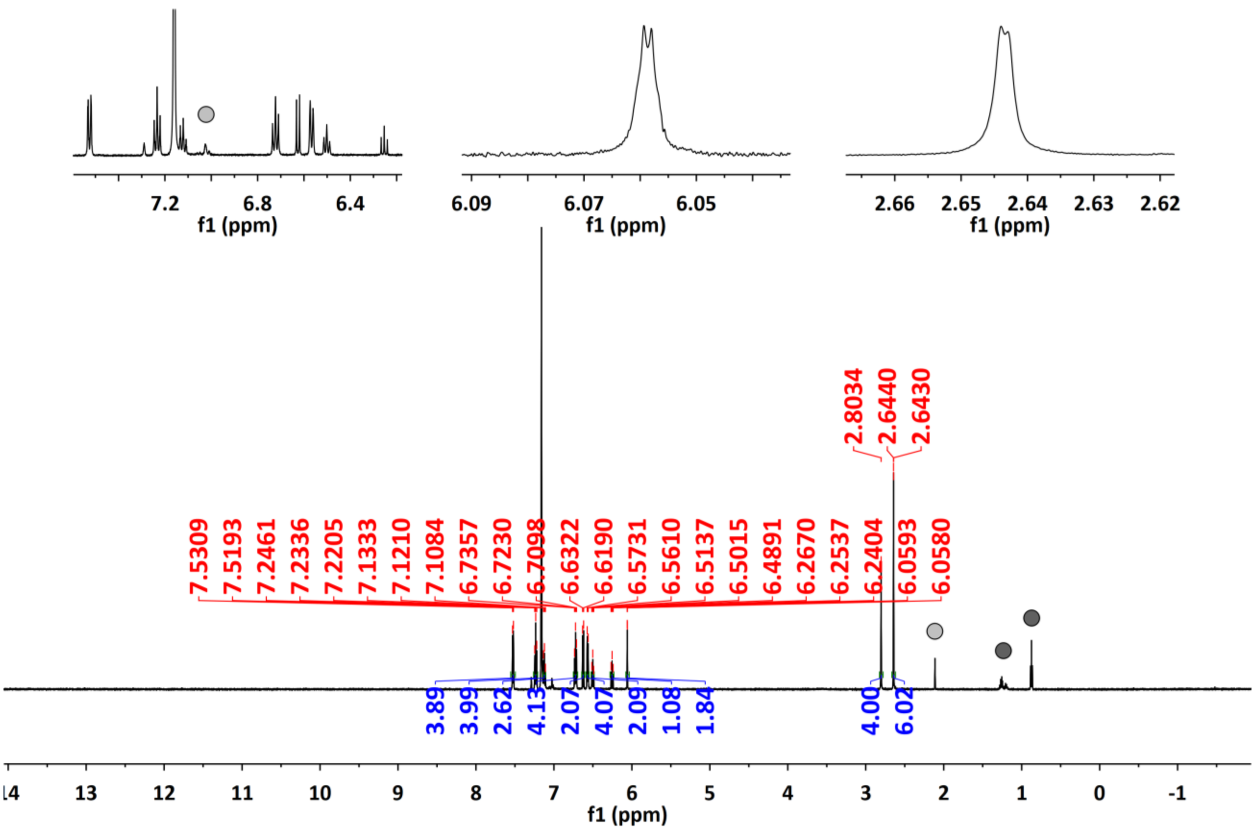

Figure S8. 600 MHz ^{1}H NMR spectrum of Hf(^{Me}PDP^{Ph})Bn_2 collected in C_6D_6. Residual toluene and pentane signals are labelled with light and dark grey circles, respectively.

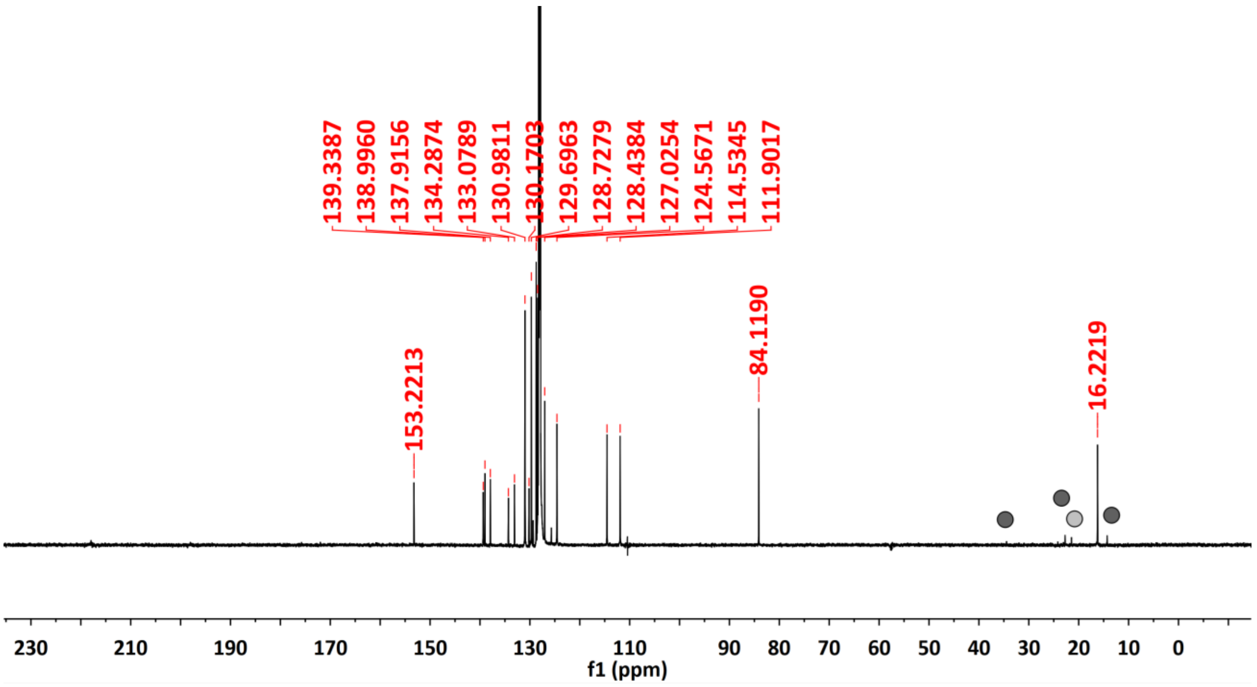

Figure S9. 151 MHz ^{13}C {^{1}H} NMR spectrum of Hf(^{Me}PDP^{Ph})Bn_2 collected in C_6D_6. An instrument artifact is evident at 110.43 ppm. Residual toluene and pentane signals are labelled with light and dark grey circles, respectively.

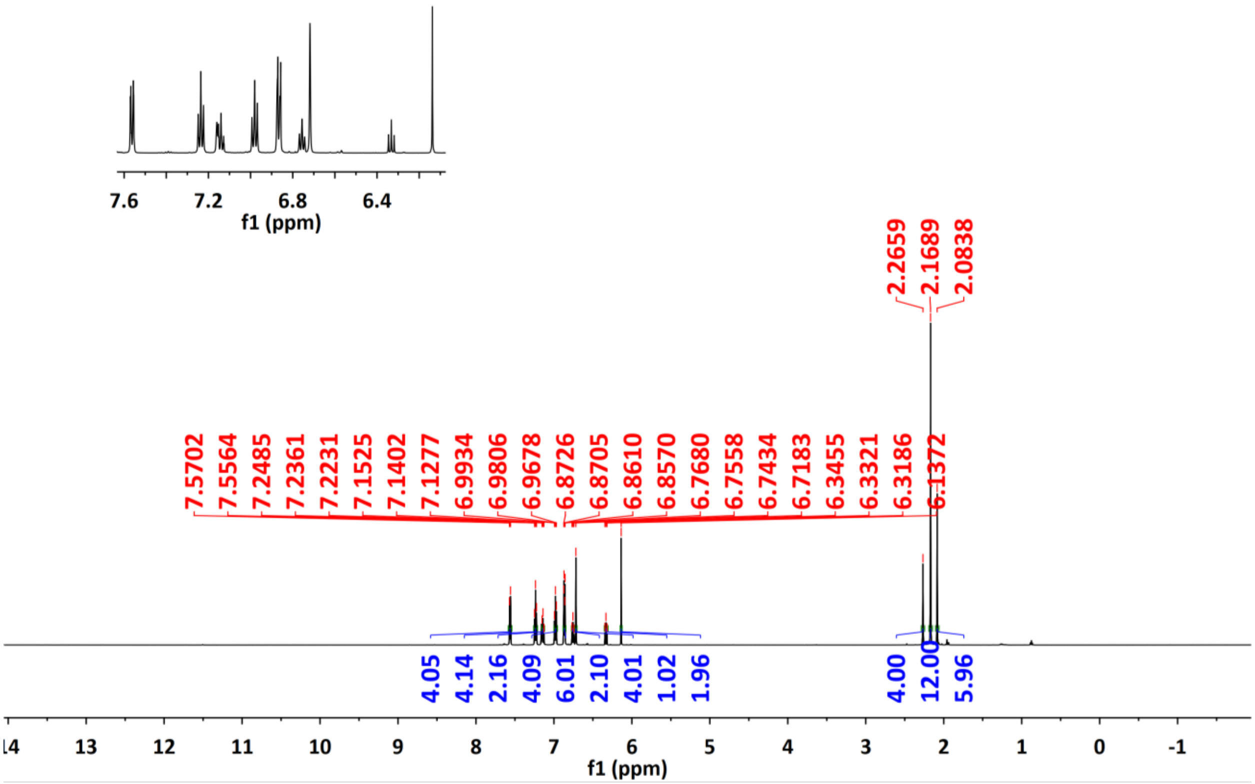

Figure S10. 600 MHz ^{1}H NMR spectrum of Hf(^{Mes}PDP^{Ph})Bn$_2$ collected in C_6D_6.

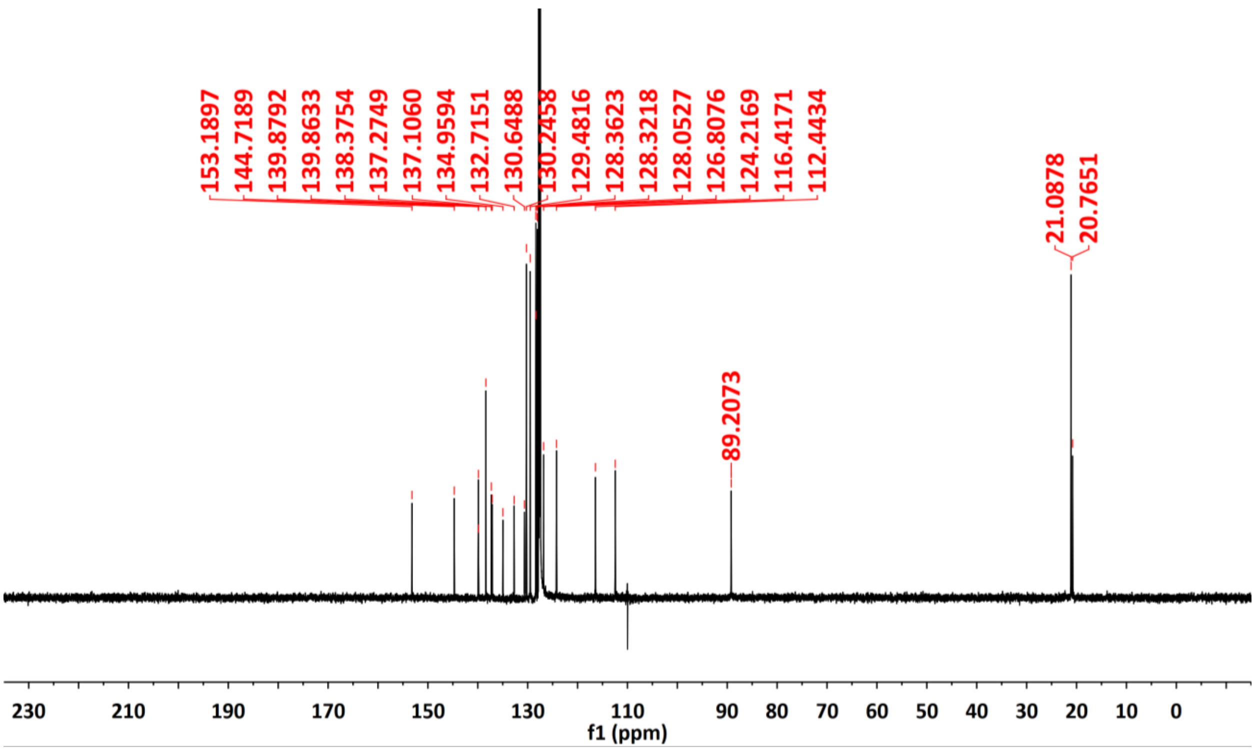

Figure S11. 151 MHz ^{13}C {^{1}H} NMR spectrum of Hf(^{Mes}PDP^{Ph})Bn$_2$ collected in C_6D_6. An instrument artifact is evident at 110.43 ppm.

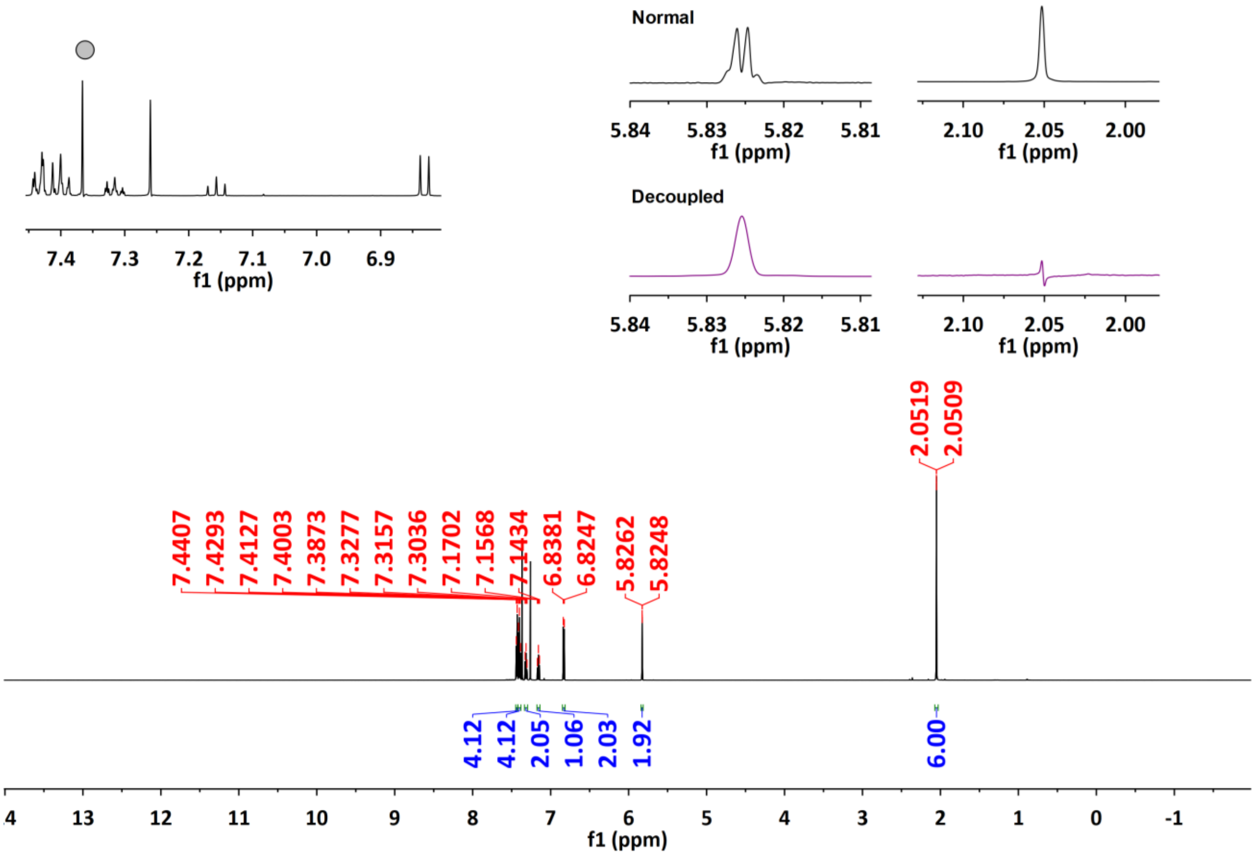

Figure S12. 600 MHz ^{1}H NMR spectrum of Hf(^{Me}PDP^{Ph})$_2$ collected in $CDCl_3$. The residual benzene signal is labelled with a grey circle. The top right inset shows the effects of selective decoupling between the pyrrolide and methyl group protons.

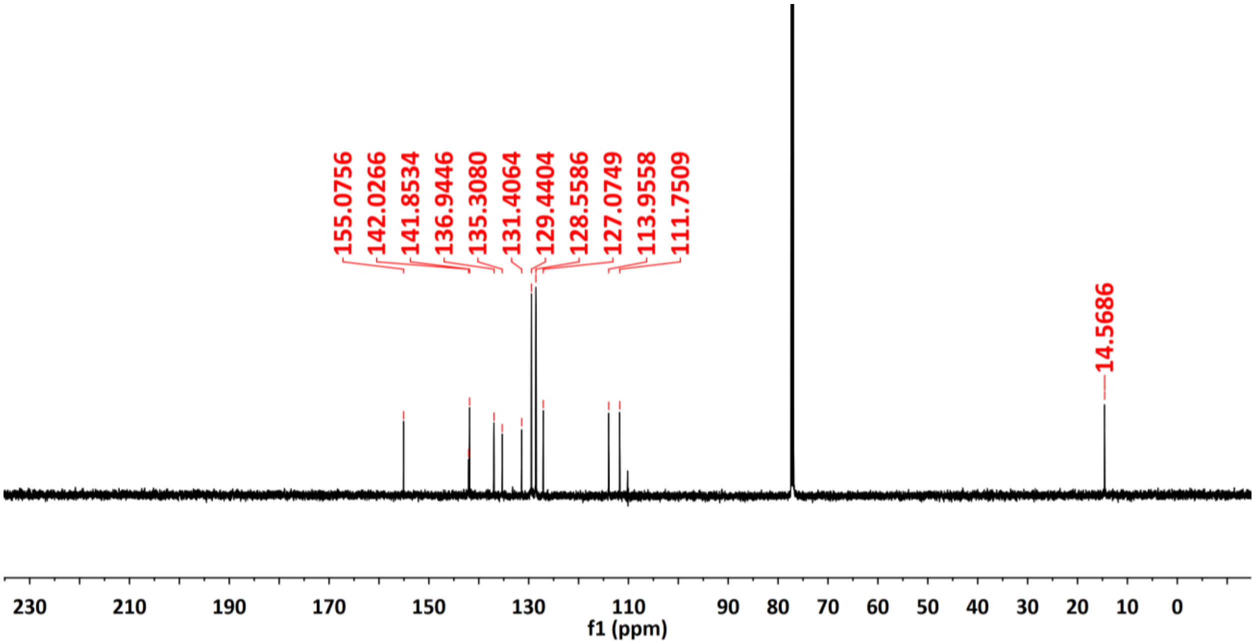

Figure S13. 151 MHz ^{13}C {^{1}H} NMR spectrum of Hf(^{Me}PDP^{Ph})$_2$ collected in $CDCl_3$. An instrument artifact is evident at 110.43 ppm.

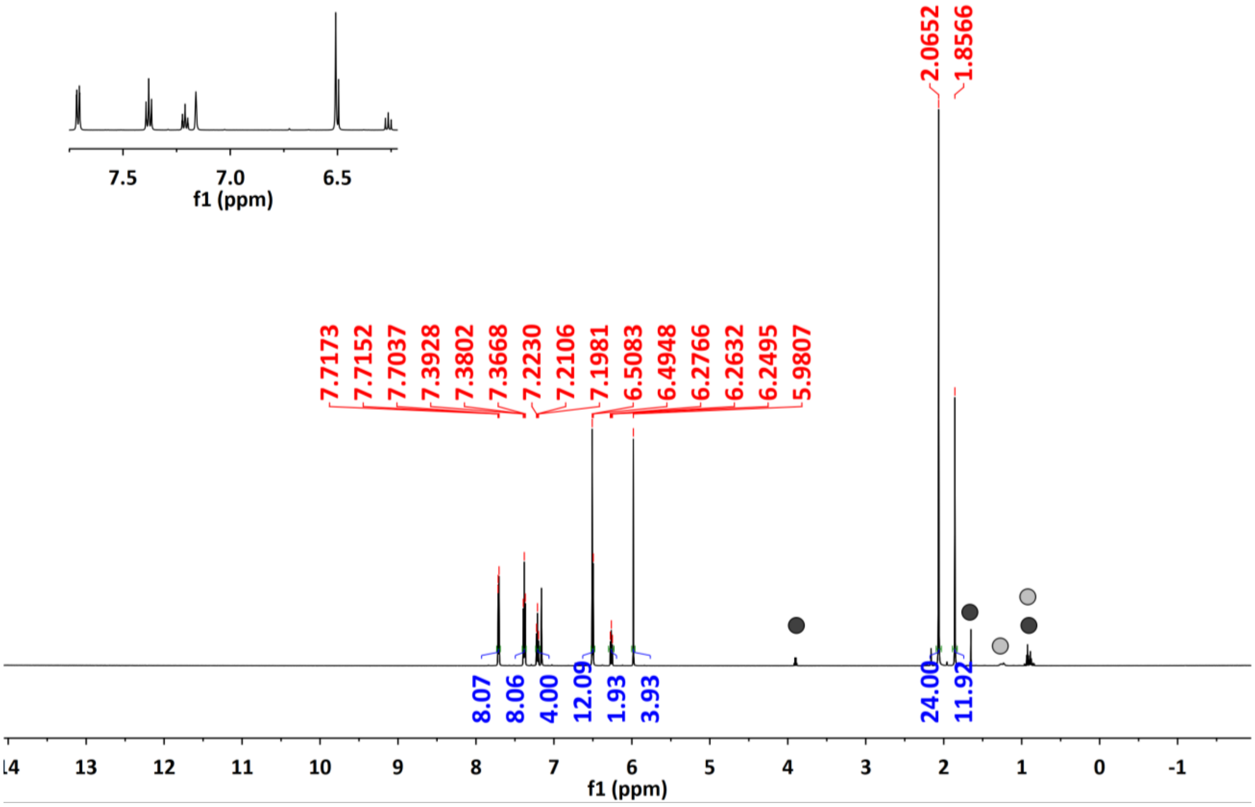

Figure S14. 600 MHz ^{1}H NMR spectrum of Hf(^{Mes}PDP^{Ph})$_2$ collected in C_6D_6. The residual ethyl acetate and hexanes signals are labelled with dark and light grey circles, respectively.

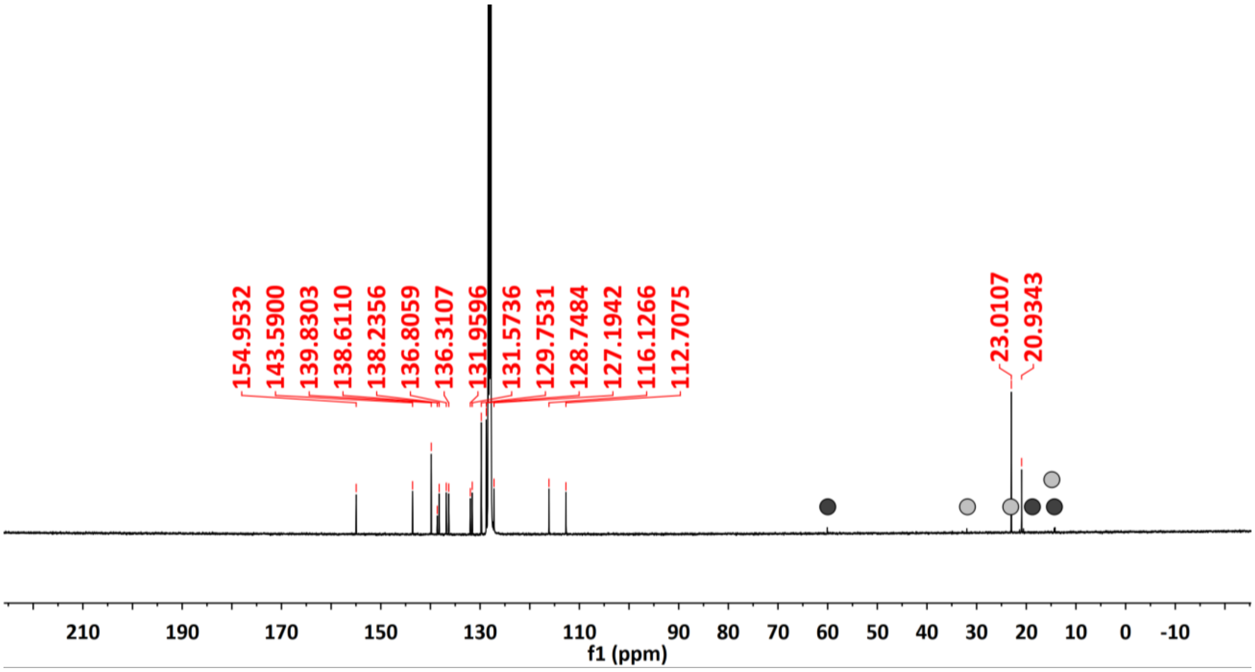

Figure S15. 101 MHz $^{13}C\{^{1}H\}$ NMR spectrum of Hf(^{Mes}PDP^{Ph})$_2$ collected in C_6D_6. The residual ethyl acetate and hexanes signals are labelled with dark and light grey circles, respectively.

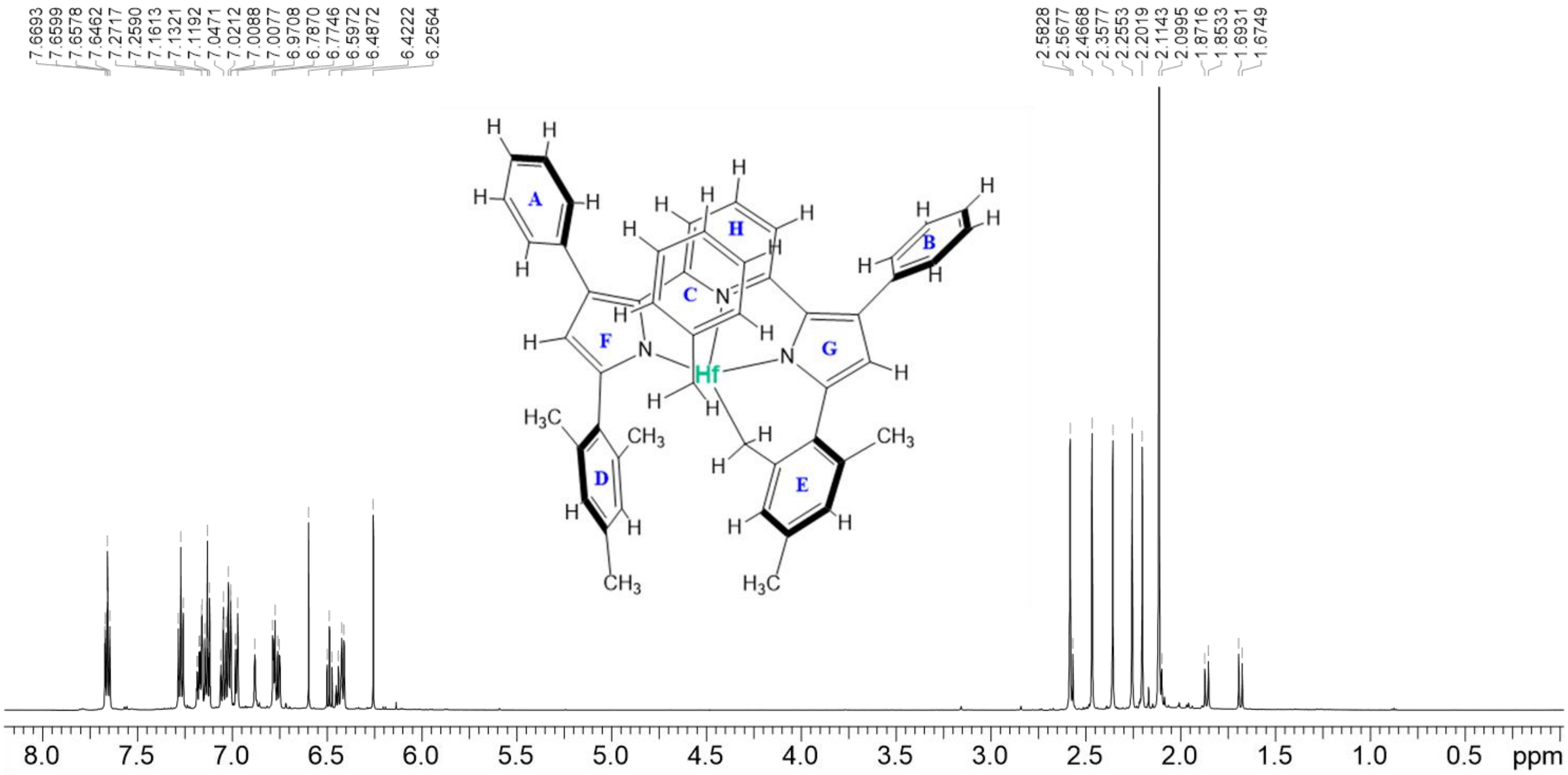

Figure S16. 600 MHz ^{1}H NMR spectrum of (cyclo-^{Mes}PDP^{Ph})HfBn collected in C_6D_6.

Integral Ratio

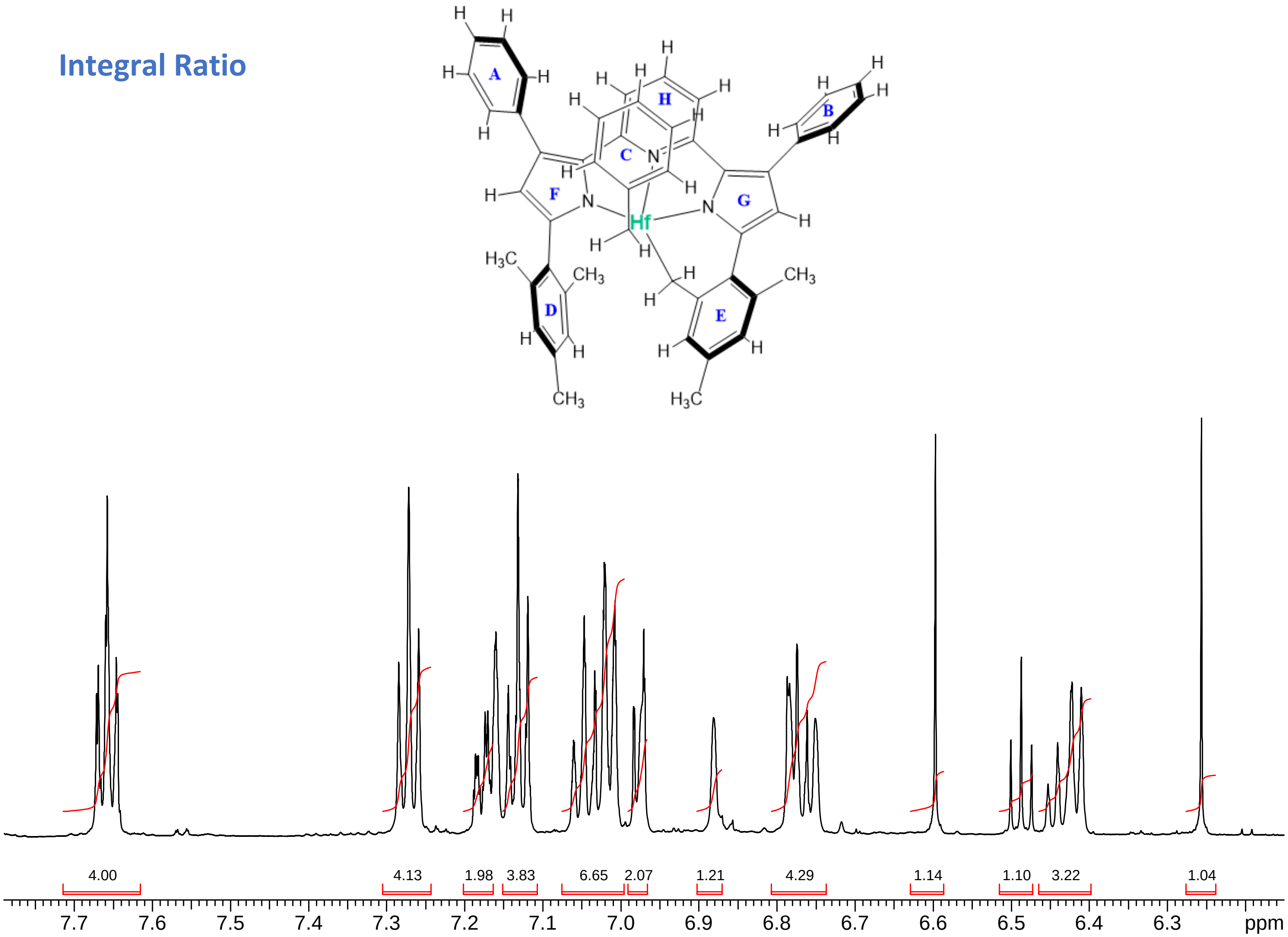

Figure S17. Expanded aromatic region of the 600 MHz ^{1}H NMR spectrum of (cyclo-^{Mes}PDP^{Ph})HfBn collected in C_6D_6.

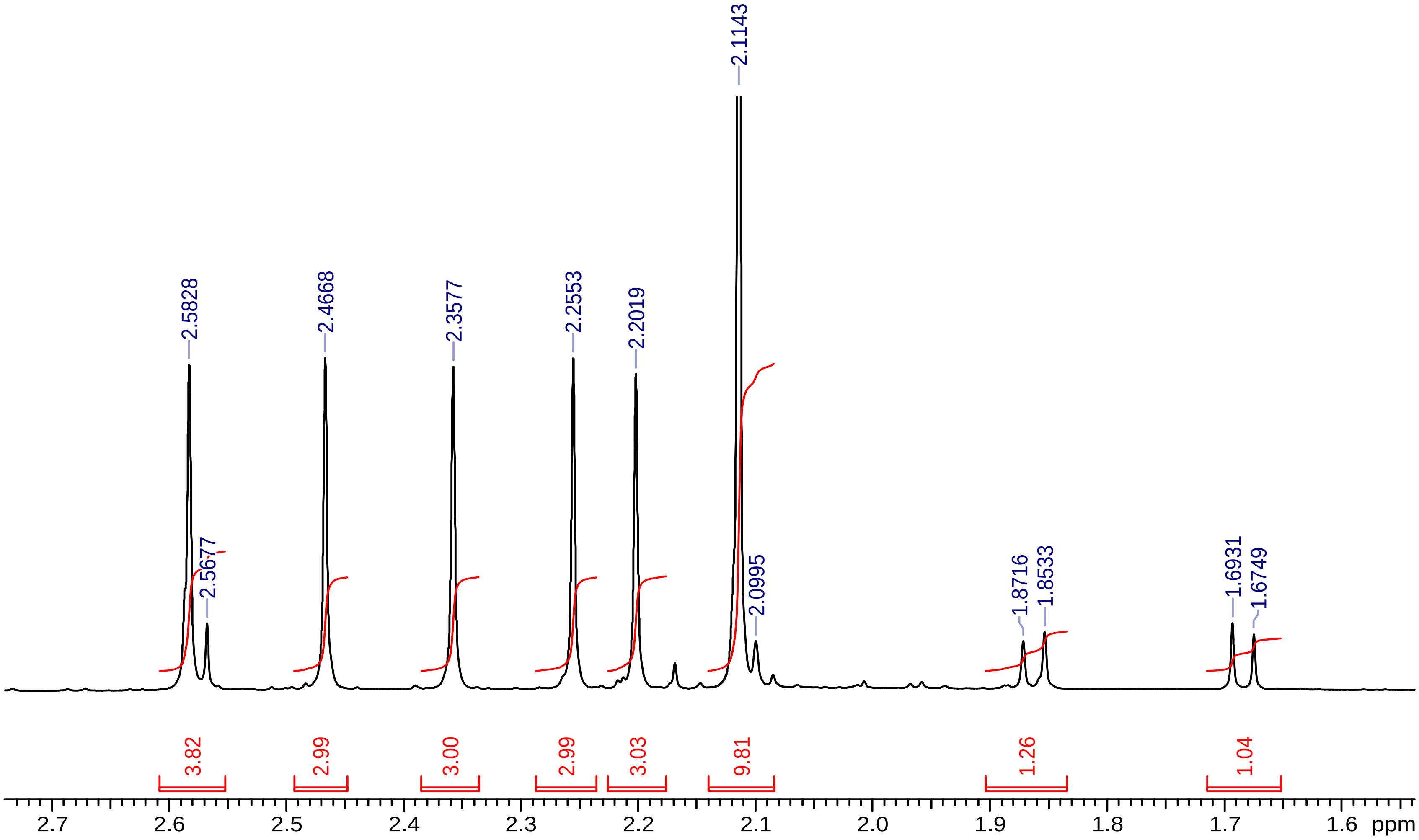

Figure S18. Expanded aliphatic region of the 600 MHz ^{1}H NMR spectrum of (cyclo-^{Mes}PDP^{Ph})HfBn in C_6D_6.

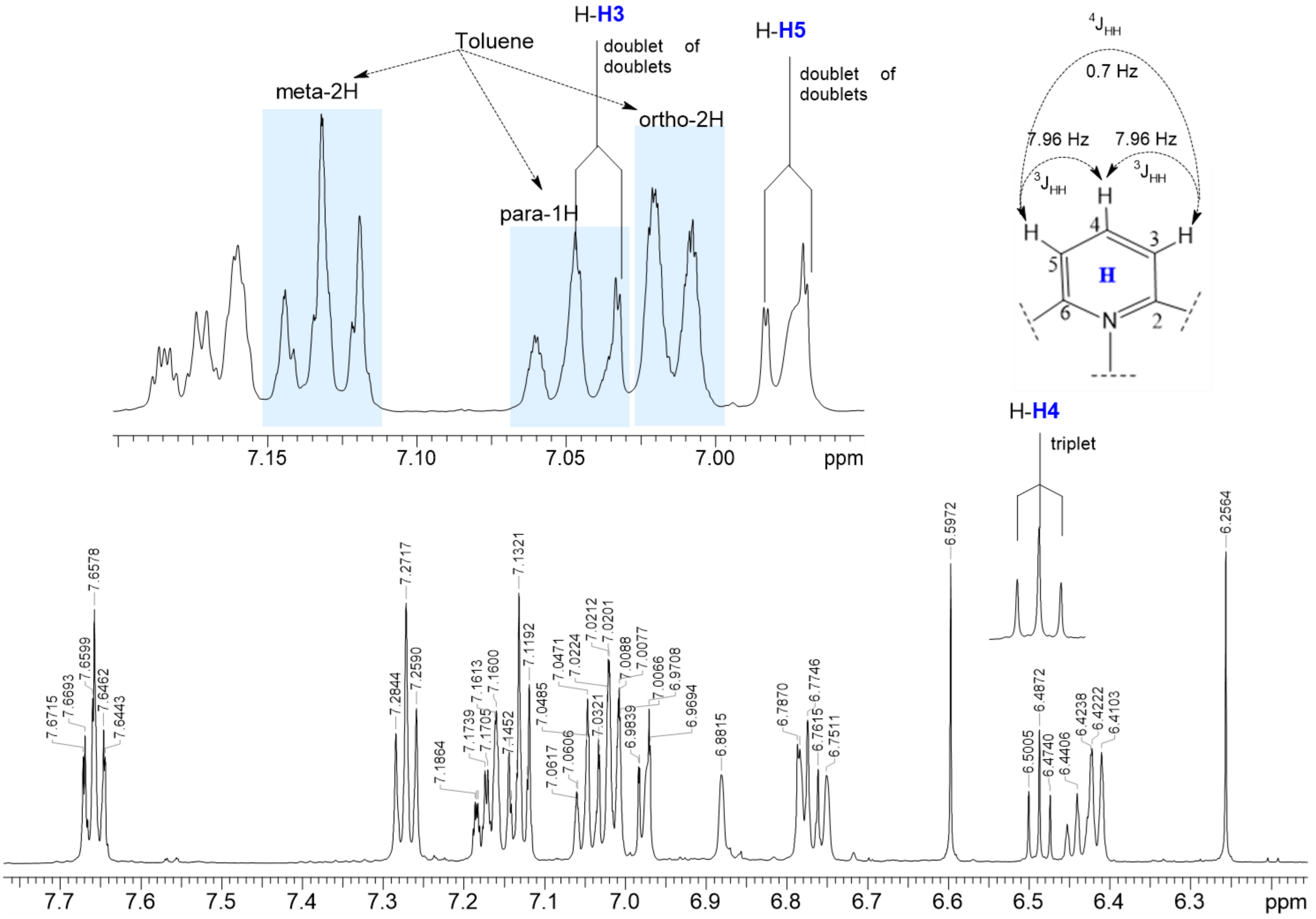

Figure S19. Assignment of the pyridine protons in (cyclo-^{Mes}PDP^{Ph})HfBn.

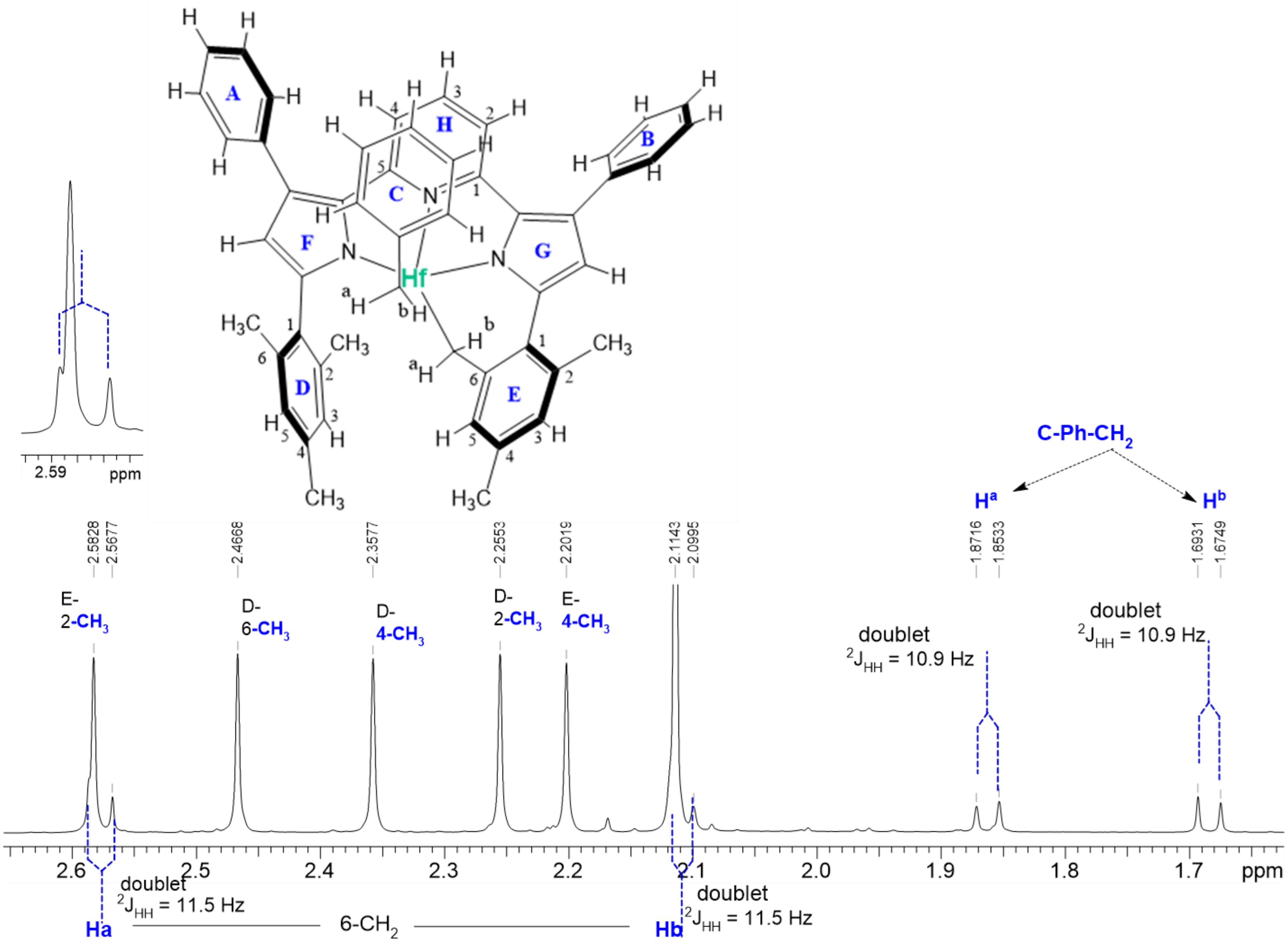

Figure S20. Assignment of the resonances corresponding to the aliphatic protons in (cyclo-^{Mes}PDP^{Ph})HfBn.

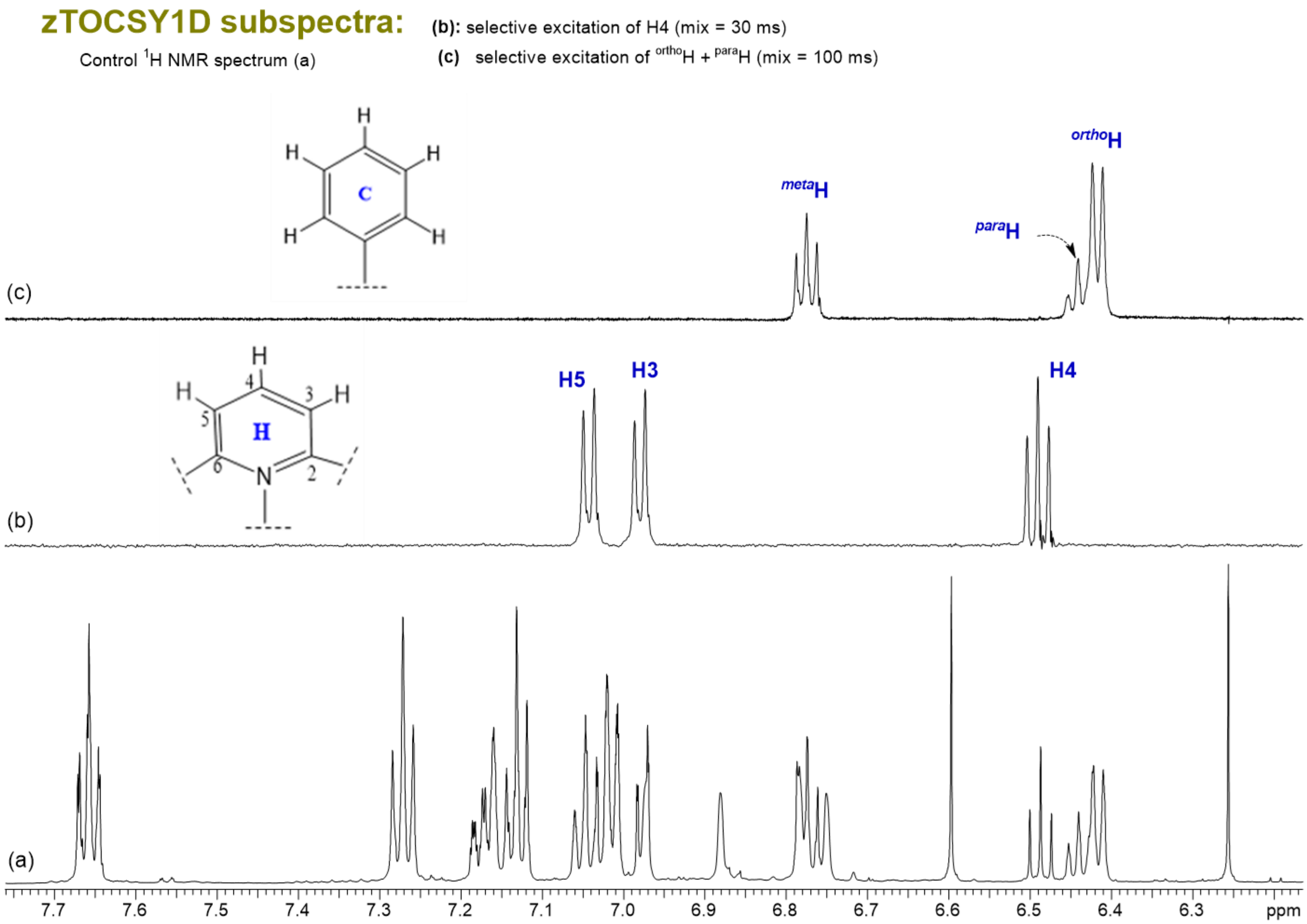

Figure S21. zTOCSY1D subspectra (b-c) of (cyclo-^{Mes}PDP^{Ph})HfBn allowing the identification of the indicated aromatic spin systems.

zTOCSY1D subspectrum:

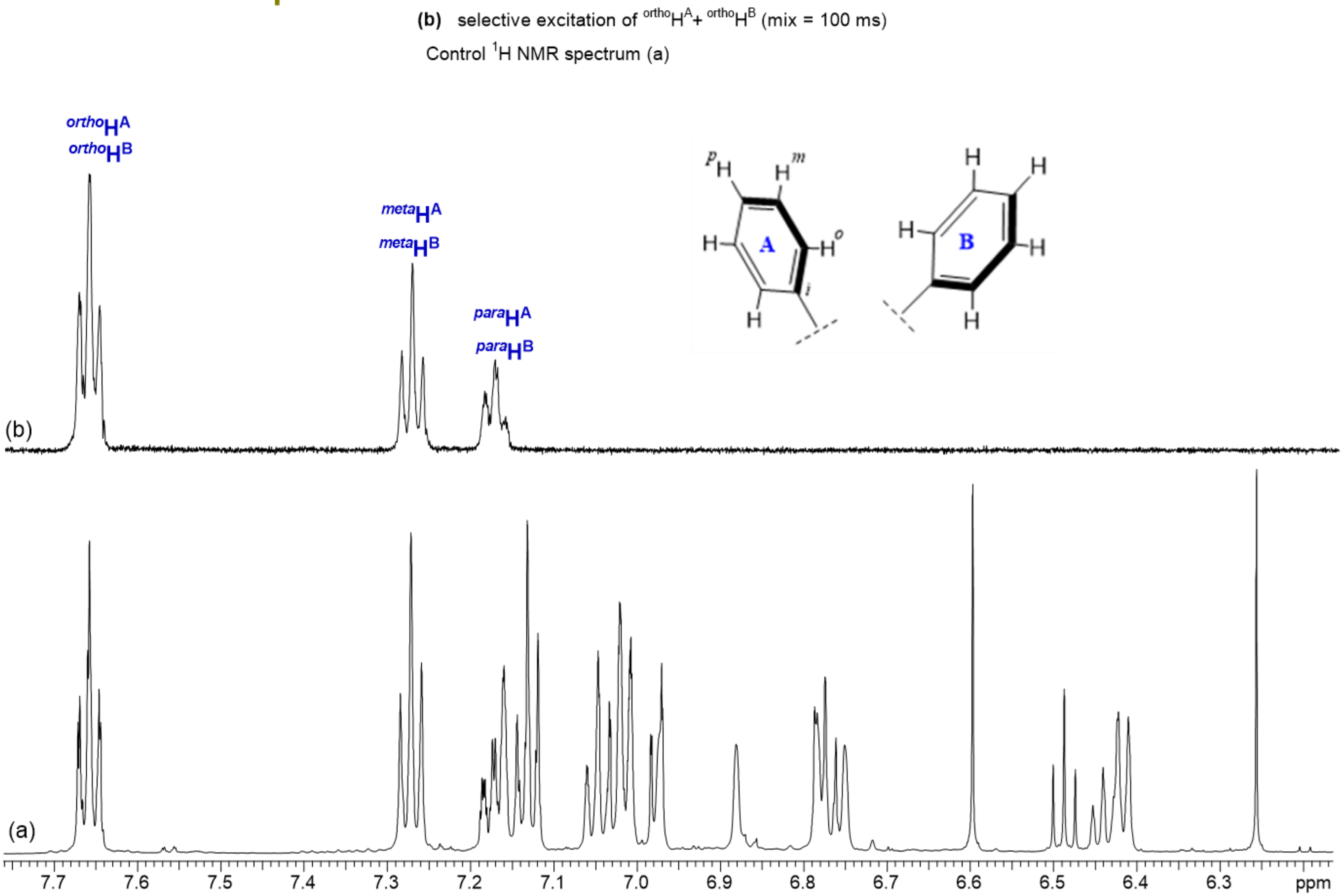

Figure S22. zTOCSY1D subspectrum (b) of (cyclo-^{Mes}PDP^{Ph})HfBn allowing the identification of the indicated aromatic spin systems.

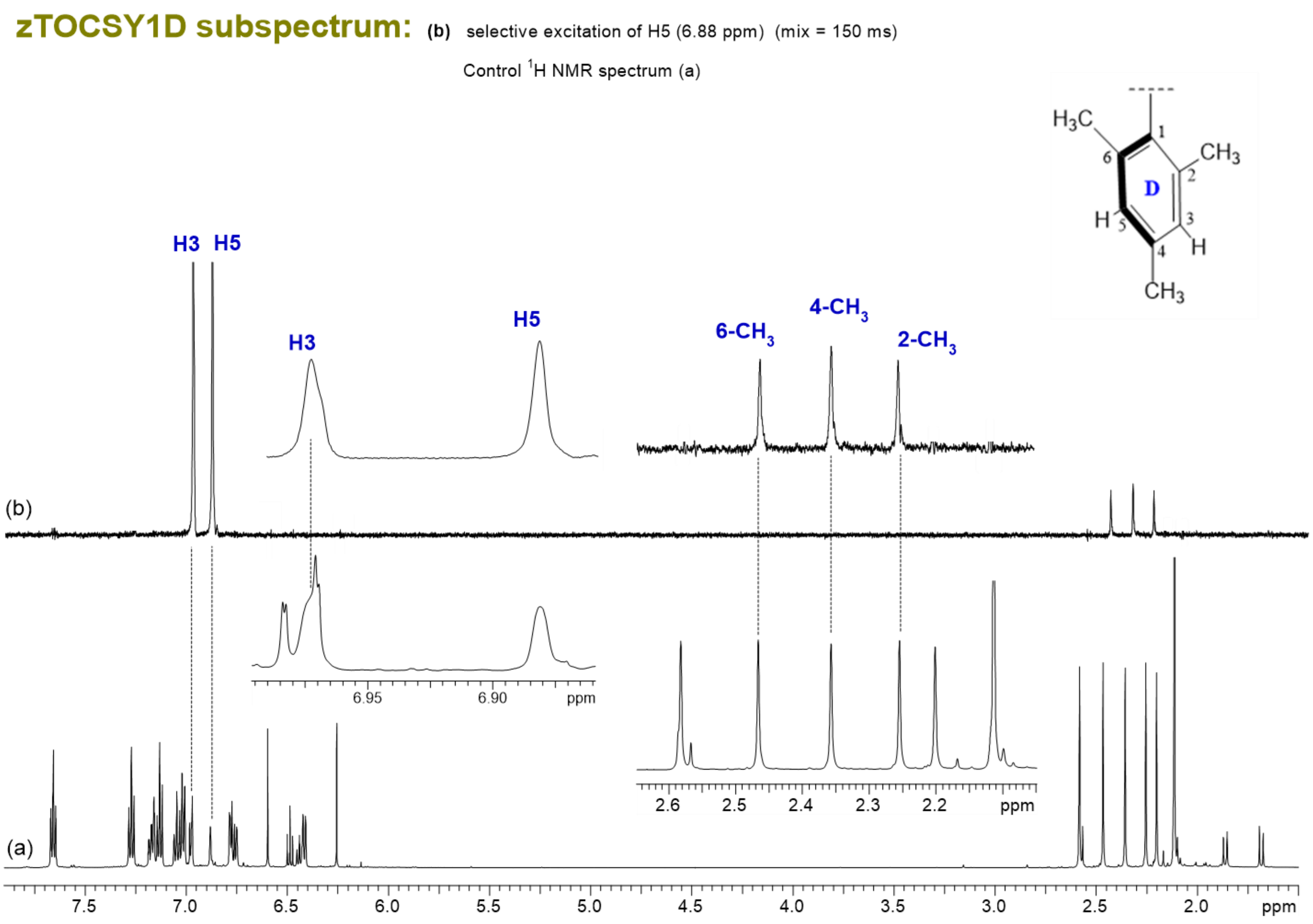

Figure S23. zTOCSY1D subspectrum (b) of (cyclo-^{Mes}PDP^{Ph})HfBn allowing the identification of the indicated resonances.

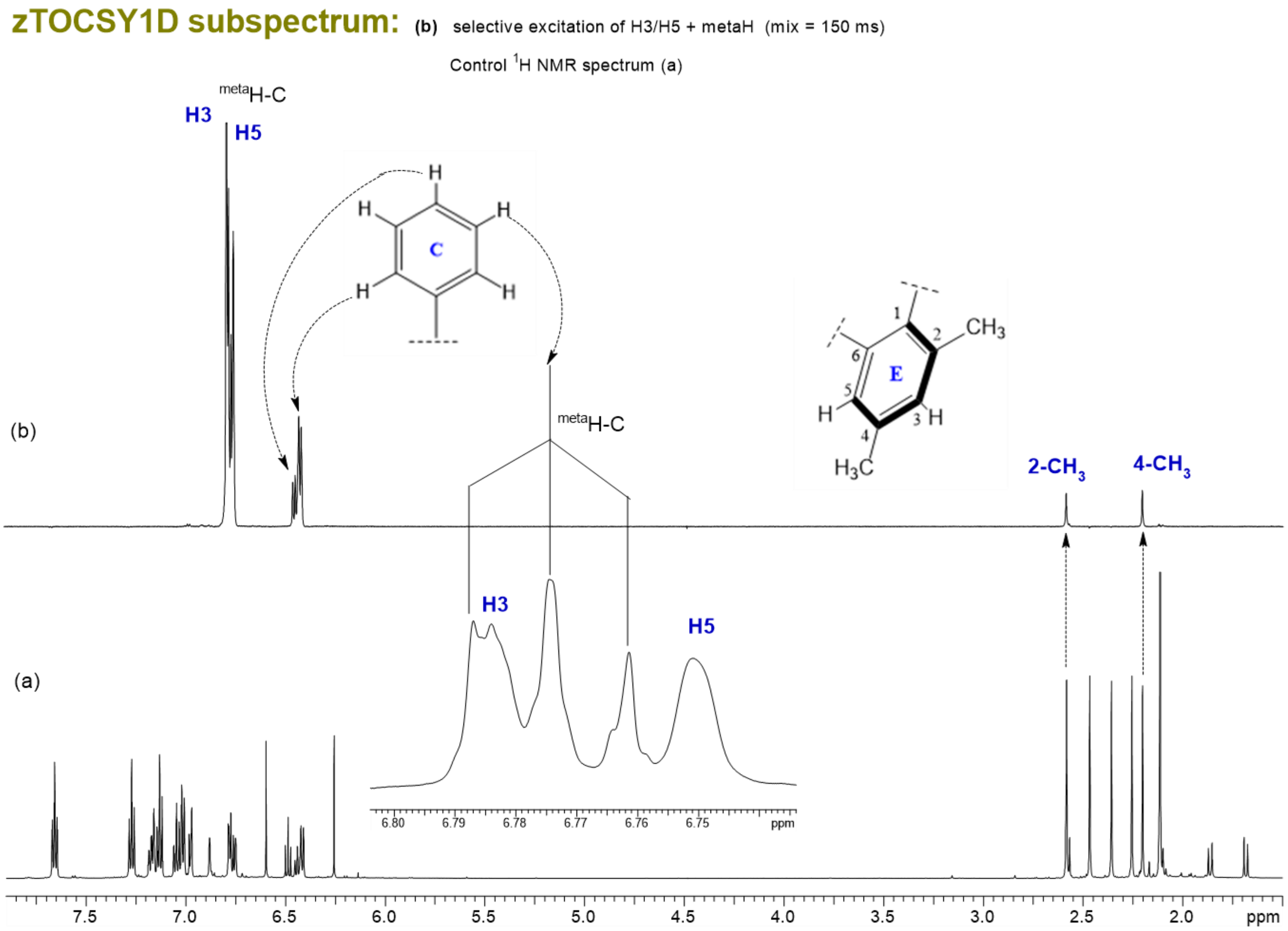

Figure S24. zTOCSY1D subspectrum (b) of (cyclo-^{Mes}PDP^{Ph})HfBn allowing the identification of the indicated resonances.

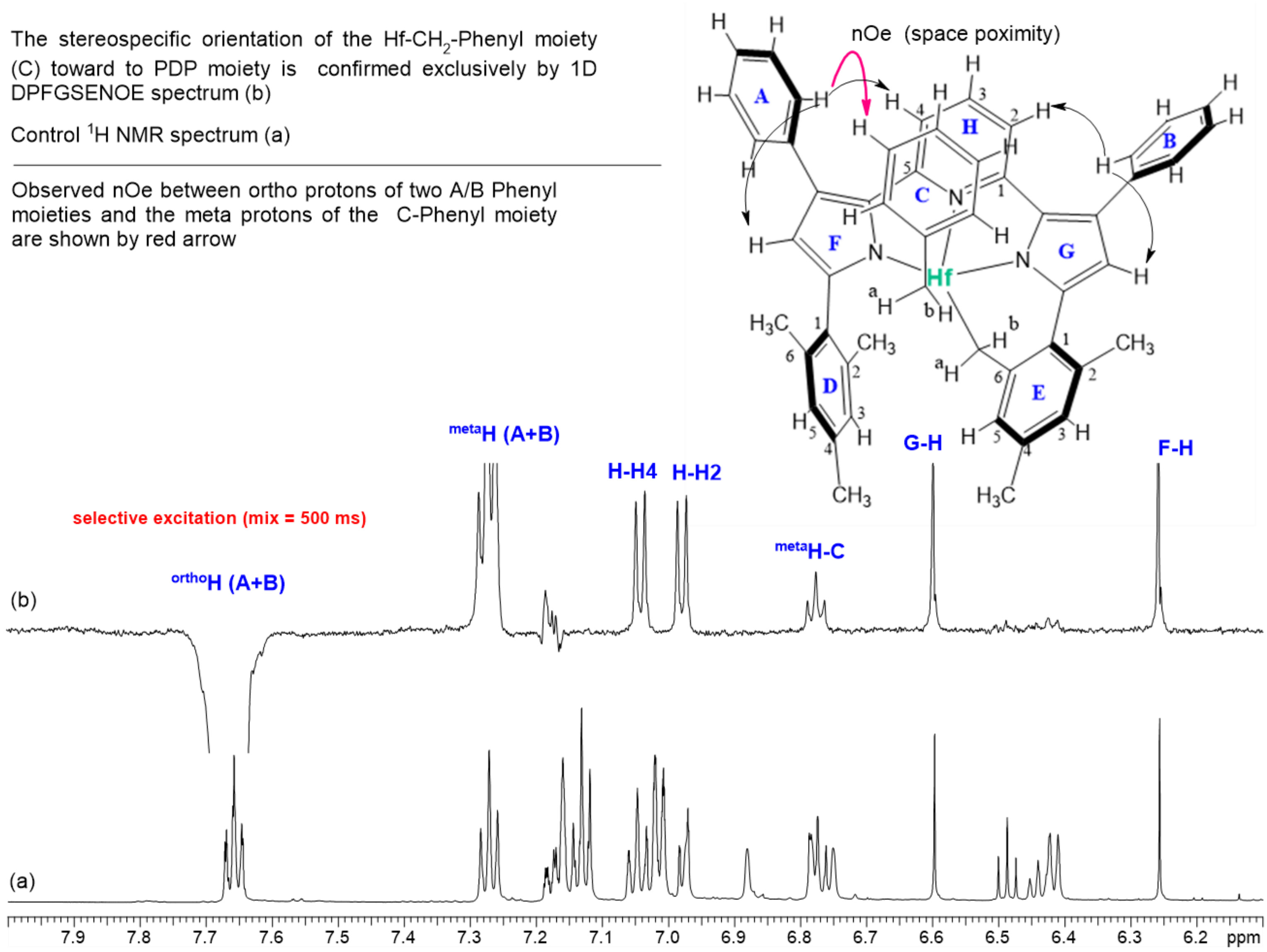

Figure S25. DPFGSENOE subspectrum (b) of (cyclo-^{Mes}PDP^{Ph})HfBn upon selective excitation of the resonance at 7.66 ppm.

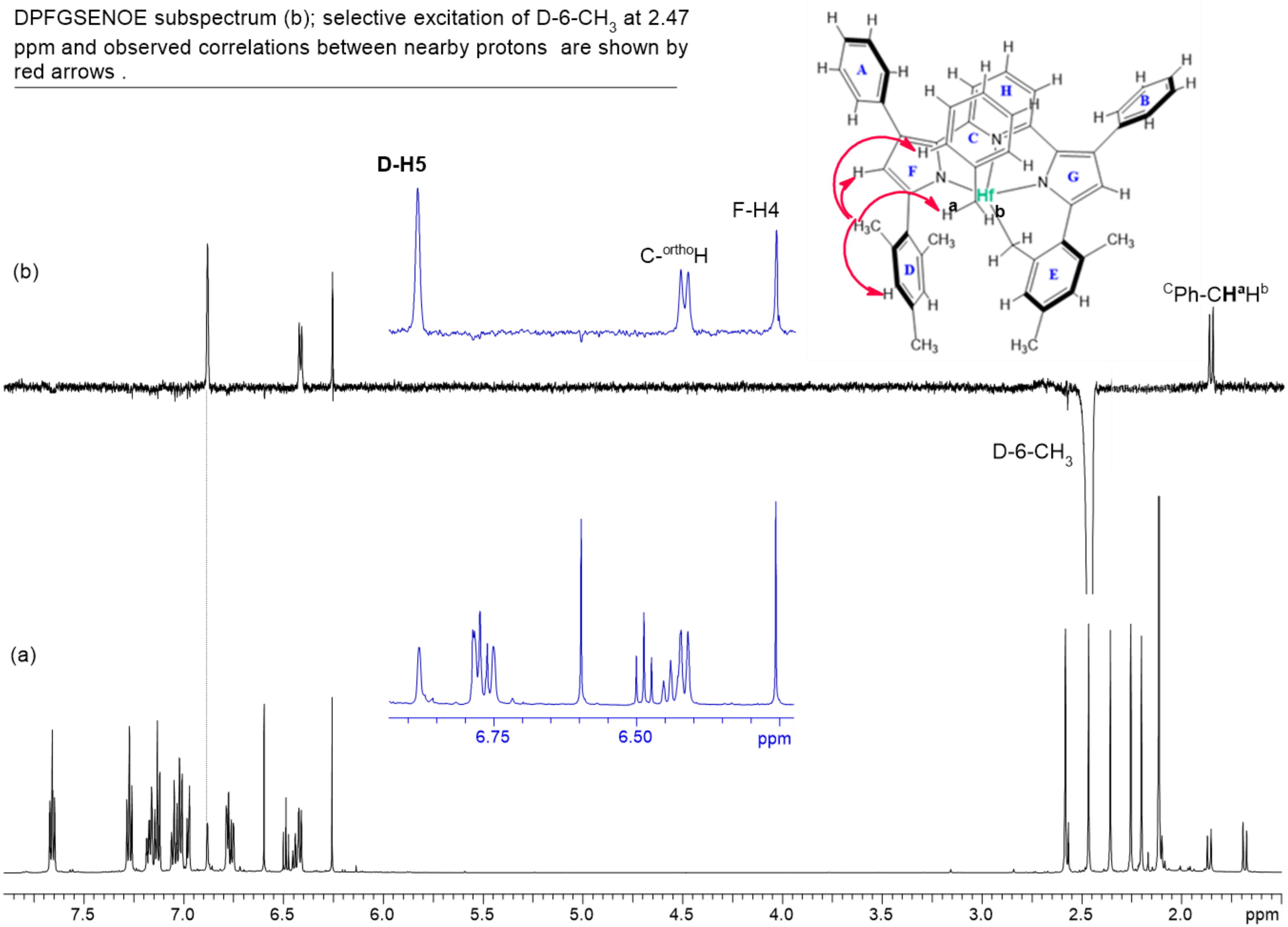

Figure S26. DPFGSENOE subspectrum (b) of (cyclo-^{Mes}PDP^{Ph})HfBn upon selective excitation of the resonance at 2.45 ppm.

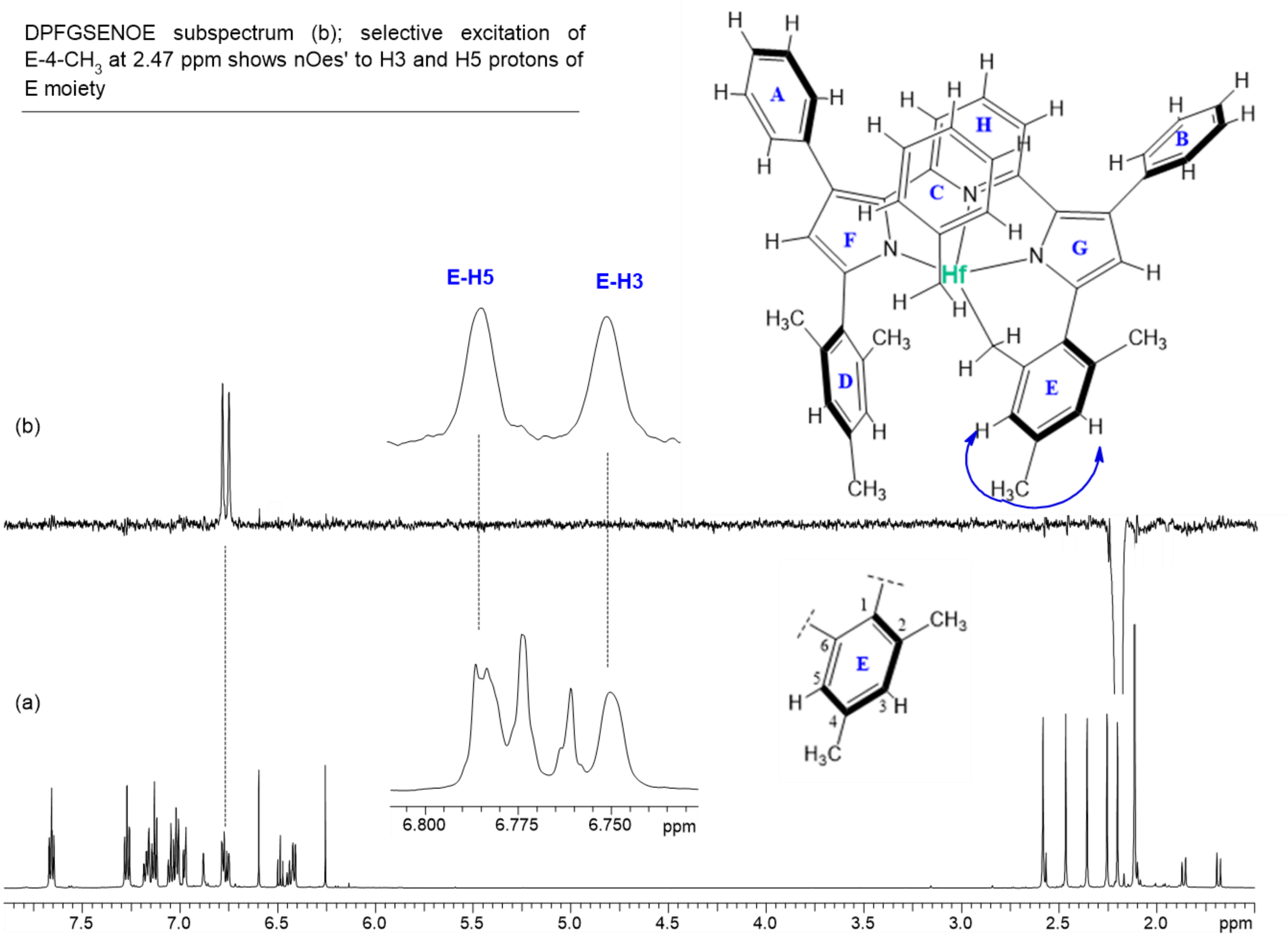

Figure S27. DPFGSENOE subspectrum (b) of (cyclo-^{Mes}PDP^{Ph})HfBn upon selective excitation of the resonance at 2.20 ppm.

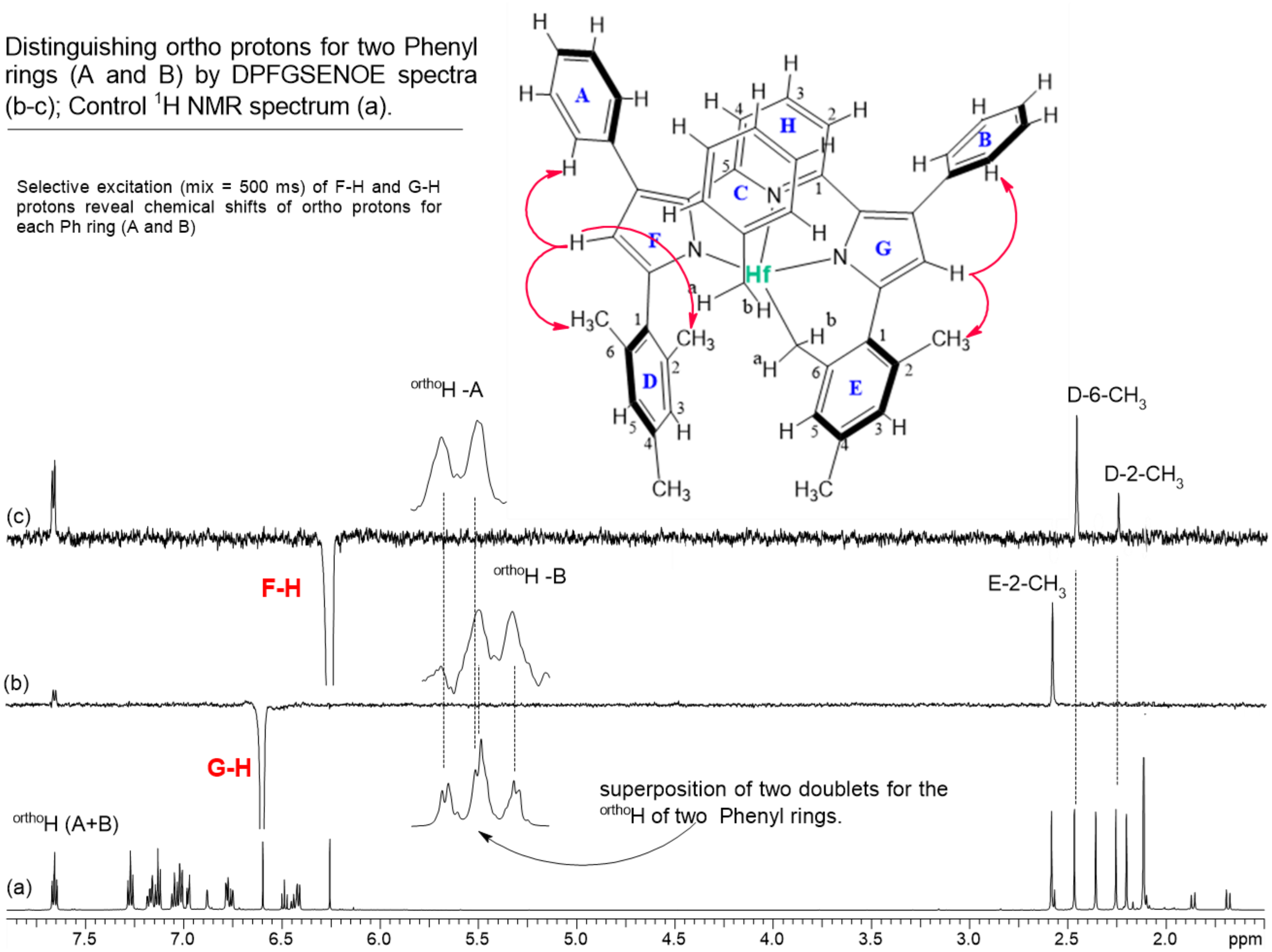

Figure S28. DPFGSENOE subspectra (b-c) of (cyclo-^{Mes}PDP^{Ph})HfBn allowing the assignment of the two phenyl substituents on the cyclo-^{Mes}PDP^{Ph} ligand.

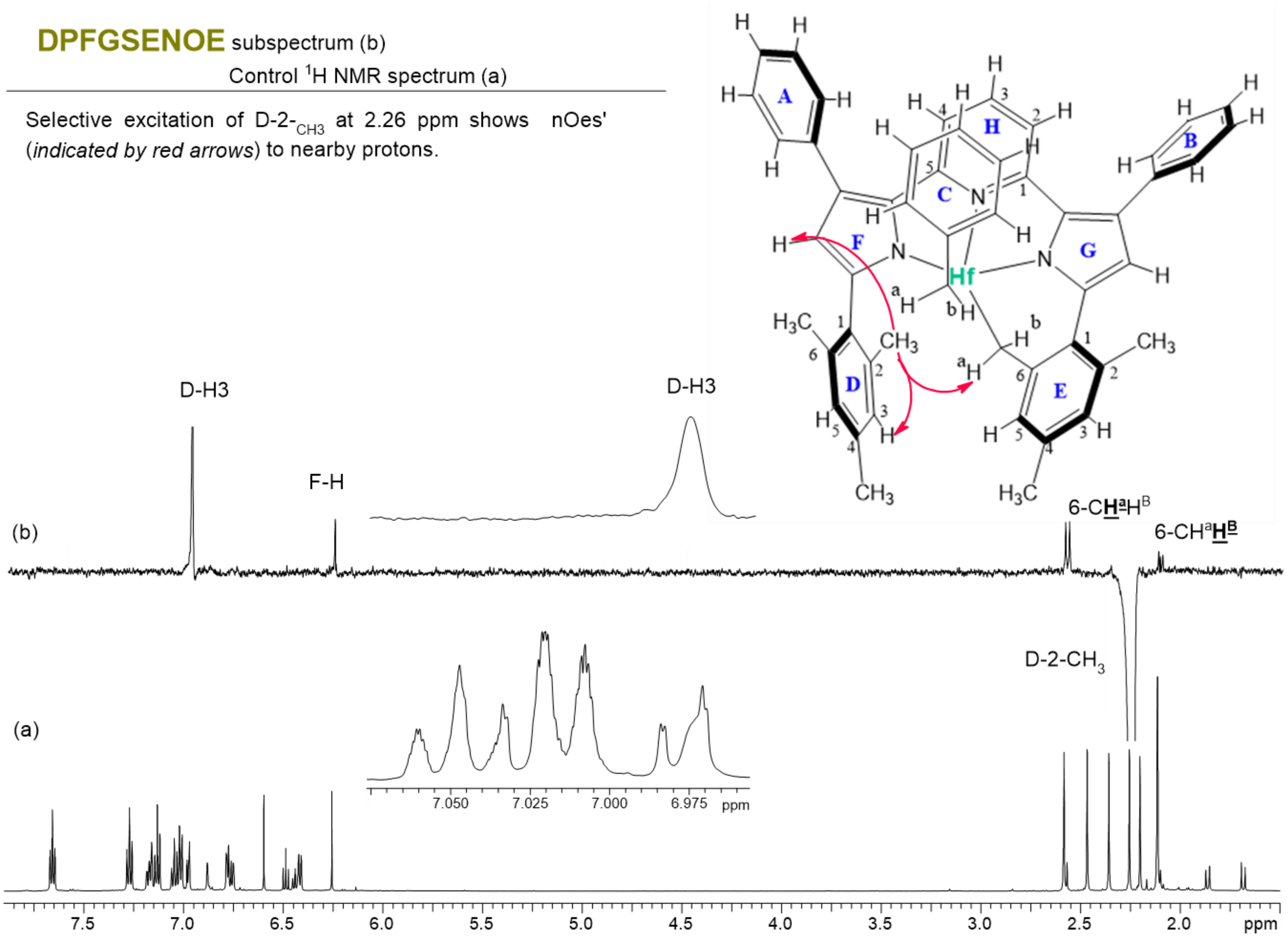

Figure S29. DPFGSENOE subspectrum (b) of (cyclo-^{Mes}PDP^{Ph})HfBn upon selective excitation of the resonance at 2.26 ppm.

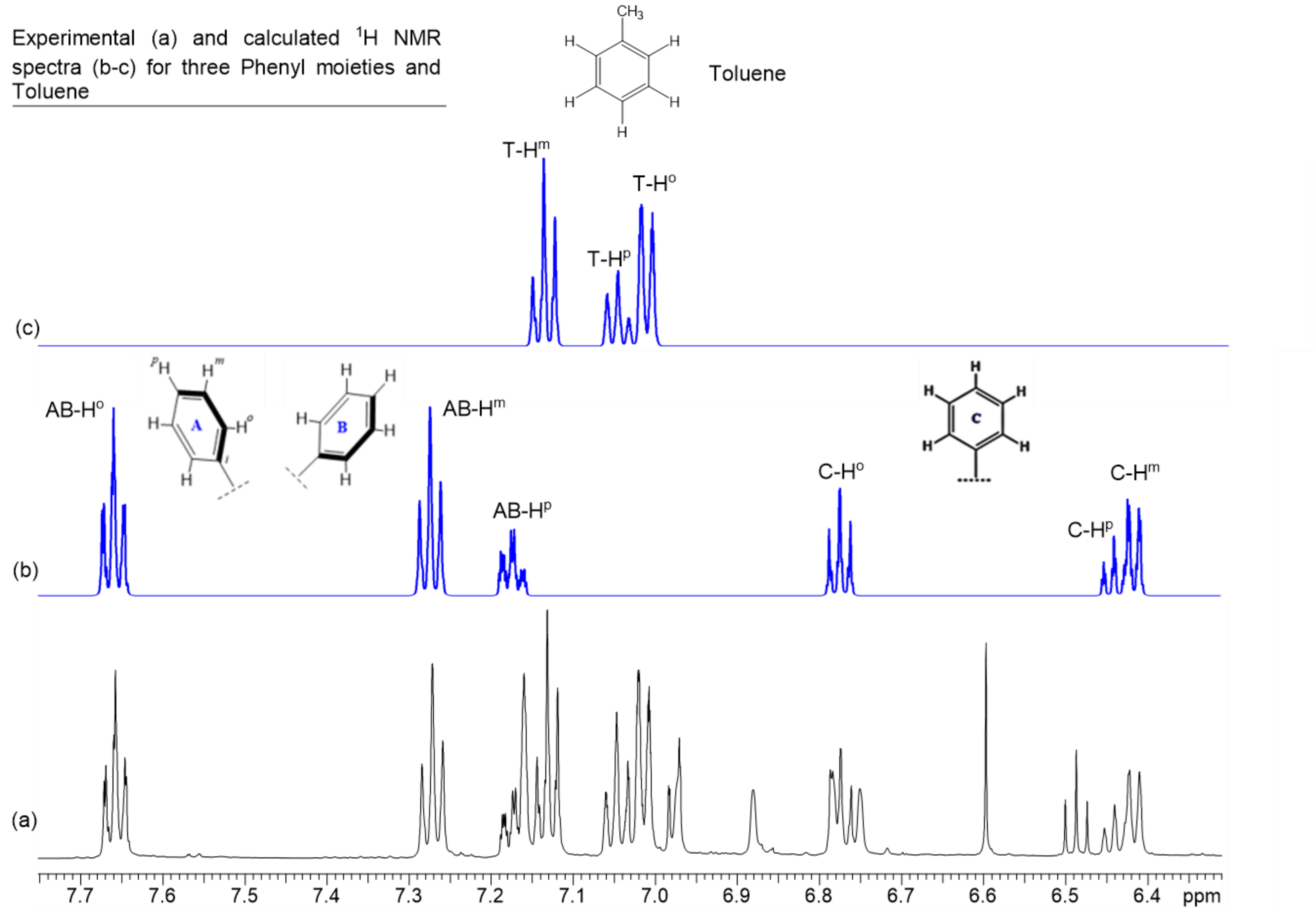

Figure S30. Comparison of the experimental and calculated ^{1}H NMR spectra for the three phenyl moieties in (cyclo-^{Mes}PDP^{Ph})HfBn.

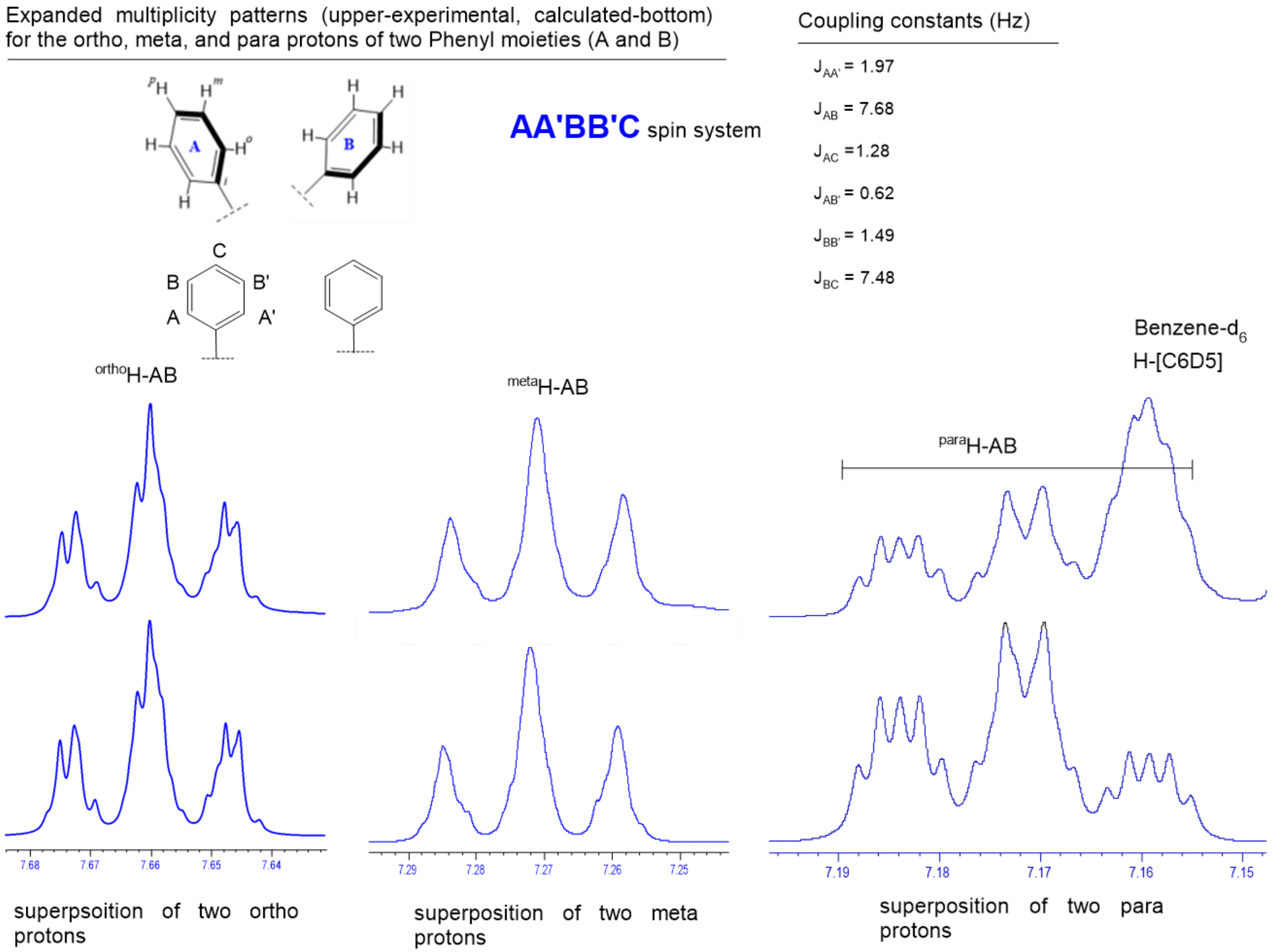

Figure S31. Expanded multiplicity patterns for the phenyl protons in (cyclo-^{Mes}PDP^{Ph})HfBn.

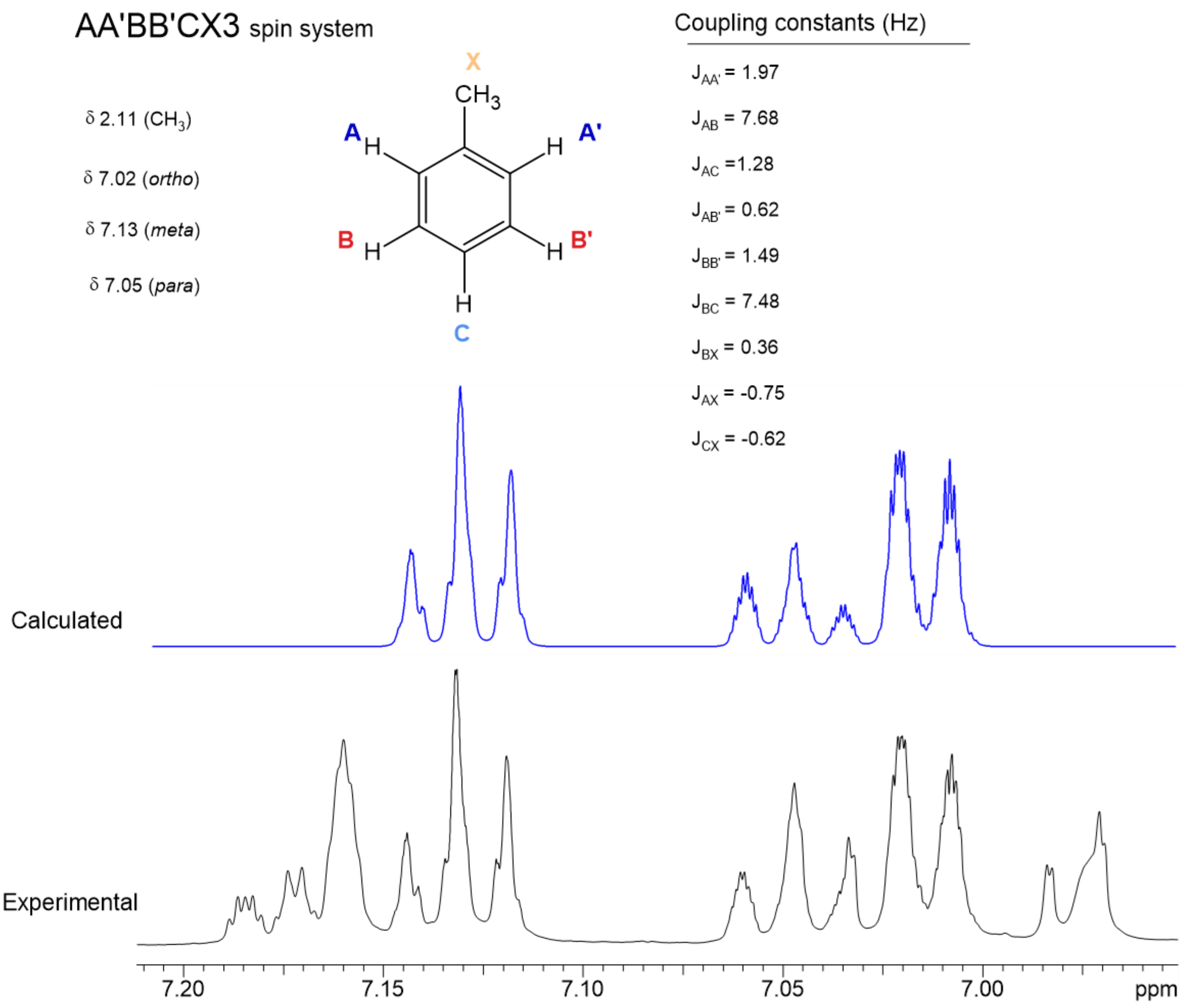

Figure S32. Calculated and experimental multiplicity patterns of ortho, meta, and para protons of toluene as an impurity in the ^{1}H NMR spectrum (cyclo-^{Mes}PDP^{Ph})HfBn.

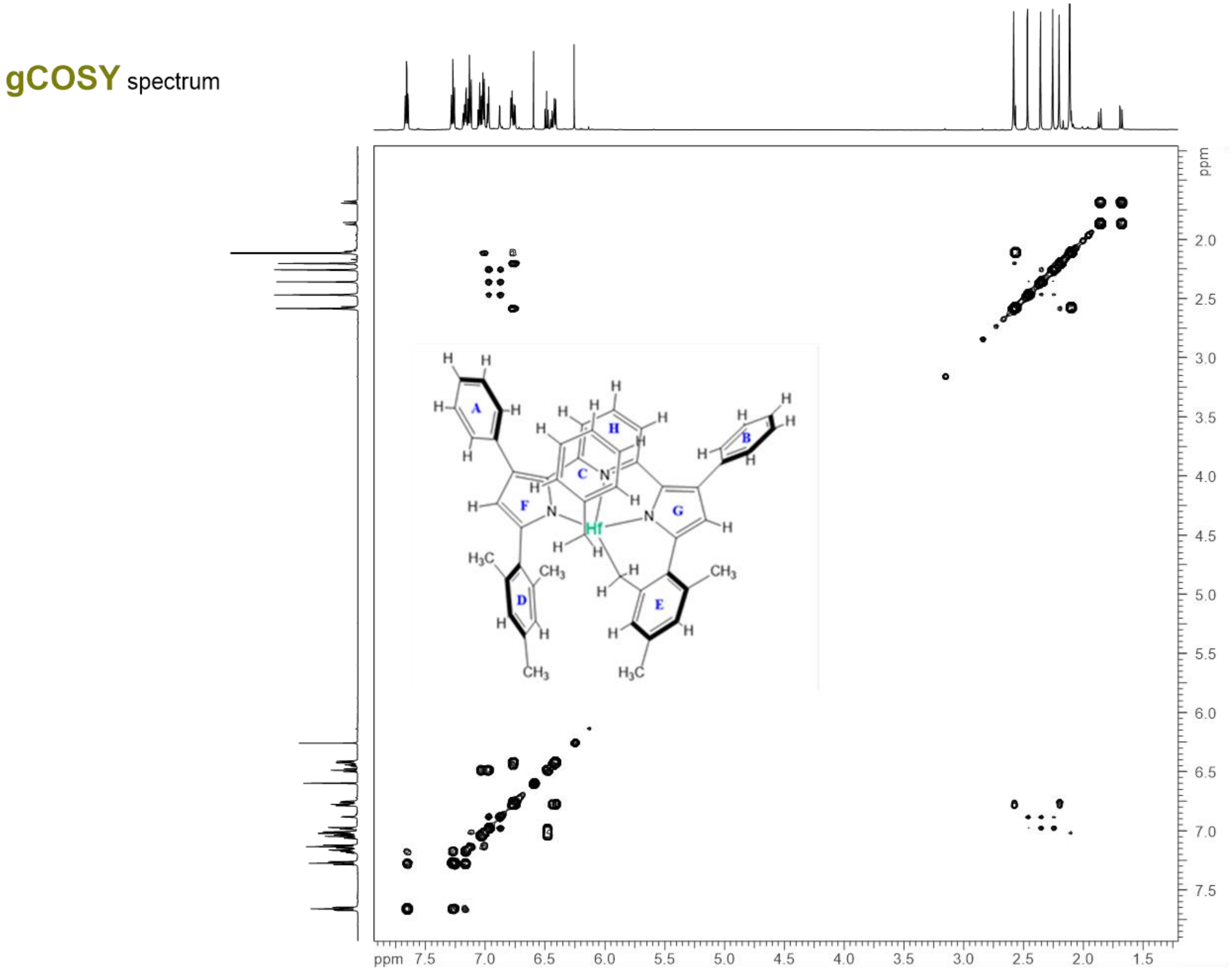

Figure S33. gCOSY spectrum of (cyclo-^{Mes}PDP^{Ph})HfBn in C_6D_6.

Expanded region of the gCOSY spectrum reveals long-range correlations between methyl protons ($^6J_{HH}$) for the D and E moieties

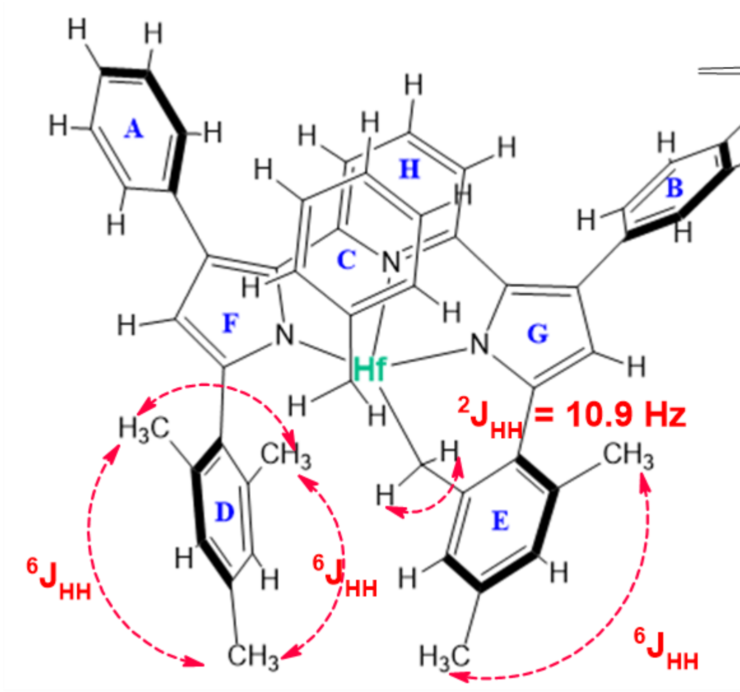

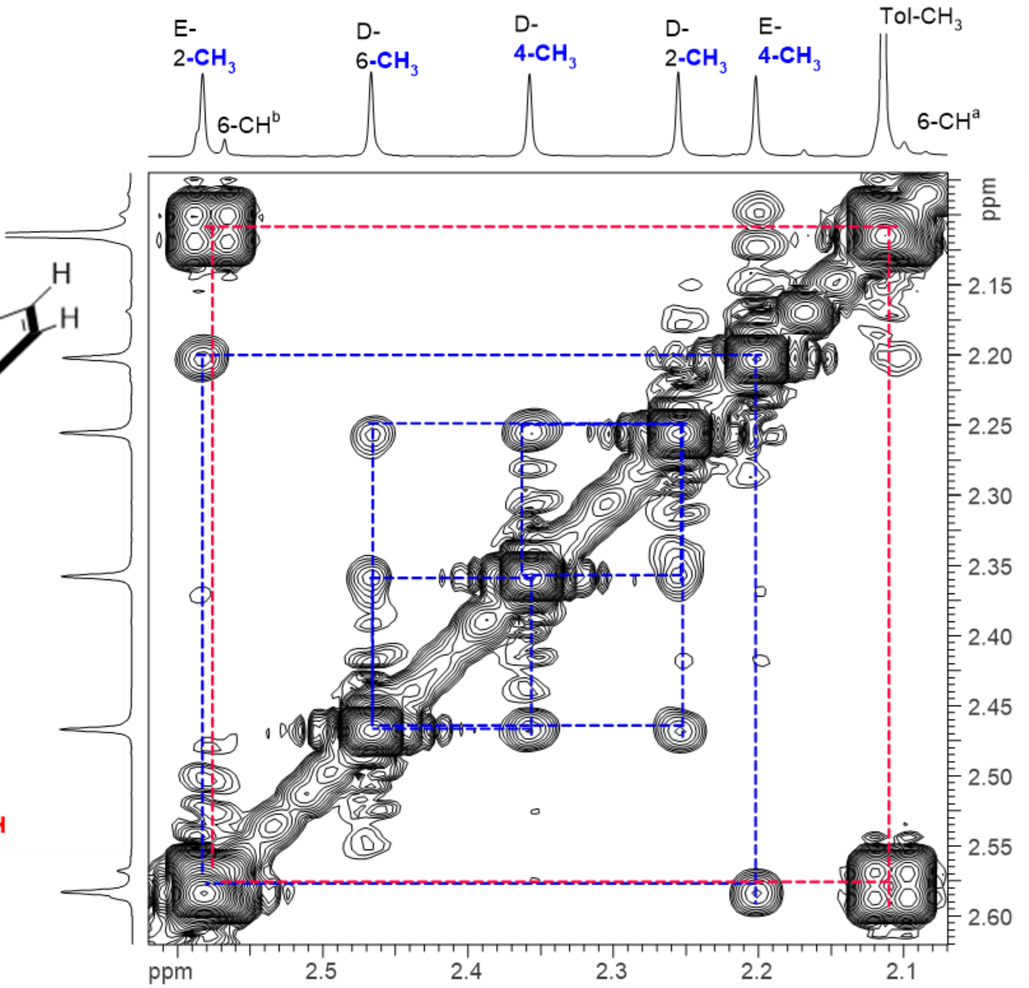

Figure S34. Expanded section of the gCOSY spectrum of (cyclo-$^{Mes}PDP^{Ph}$)HfBn in C_6D_6.

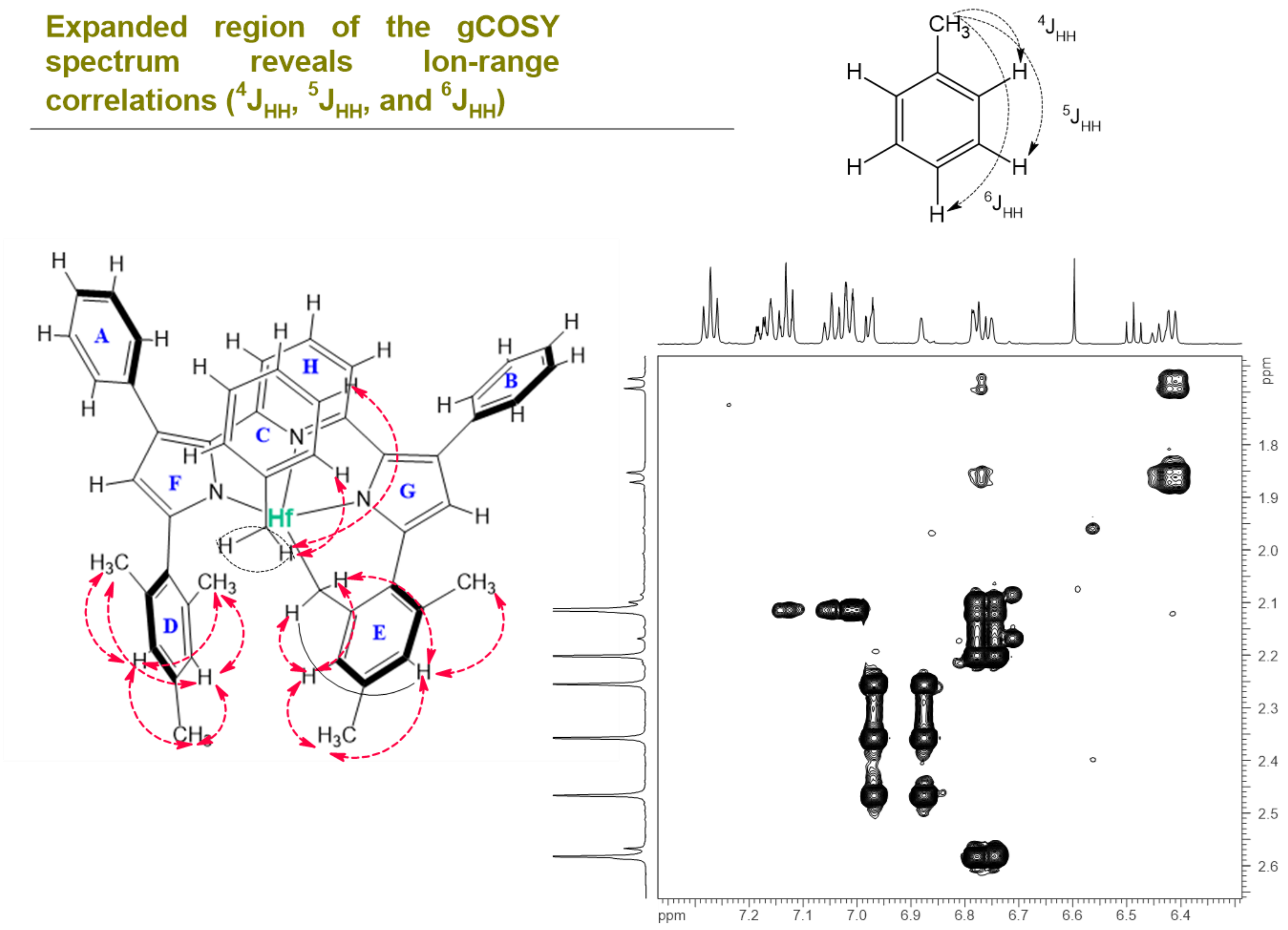

Figure S35. Expanded section of the gCOSY spectrum of (cyclo-^{Mes}PDP^{Ph})HfBn in C_6D_6.

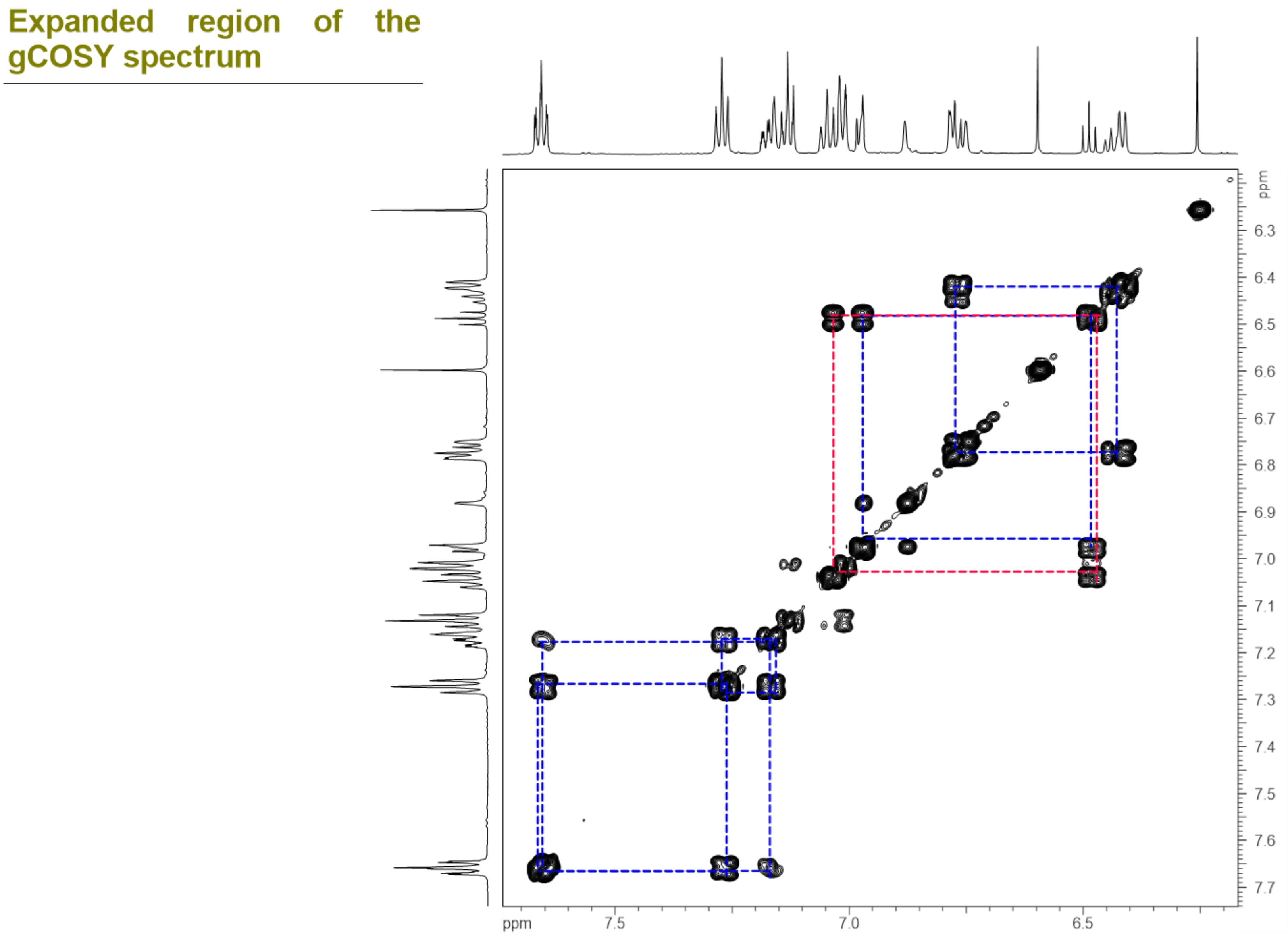

Figure S36. Expanded section of the gCOSY spectrum of (cyclo-^{Mes}PDP^{Ph})HfBn in C_6D_6.

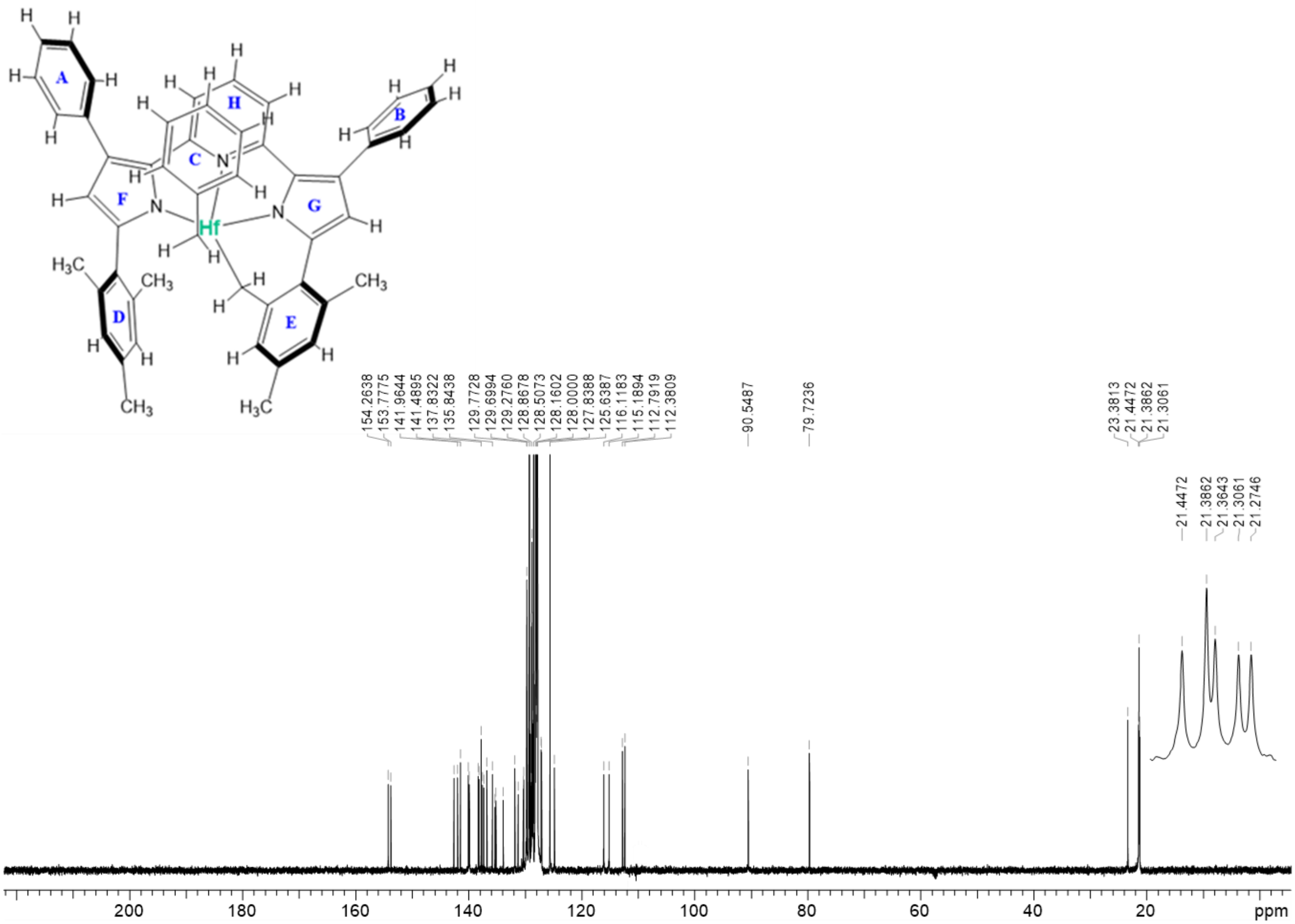

Figure S37. 125 MHz ^{13}C {^{1}H} NMR spectrum of (cyclo-^{Mes}PDP^{Ph})HfBn in C_6D_6.

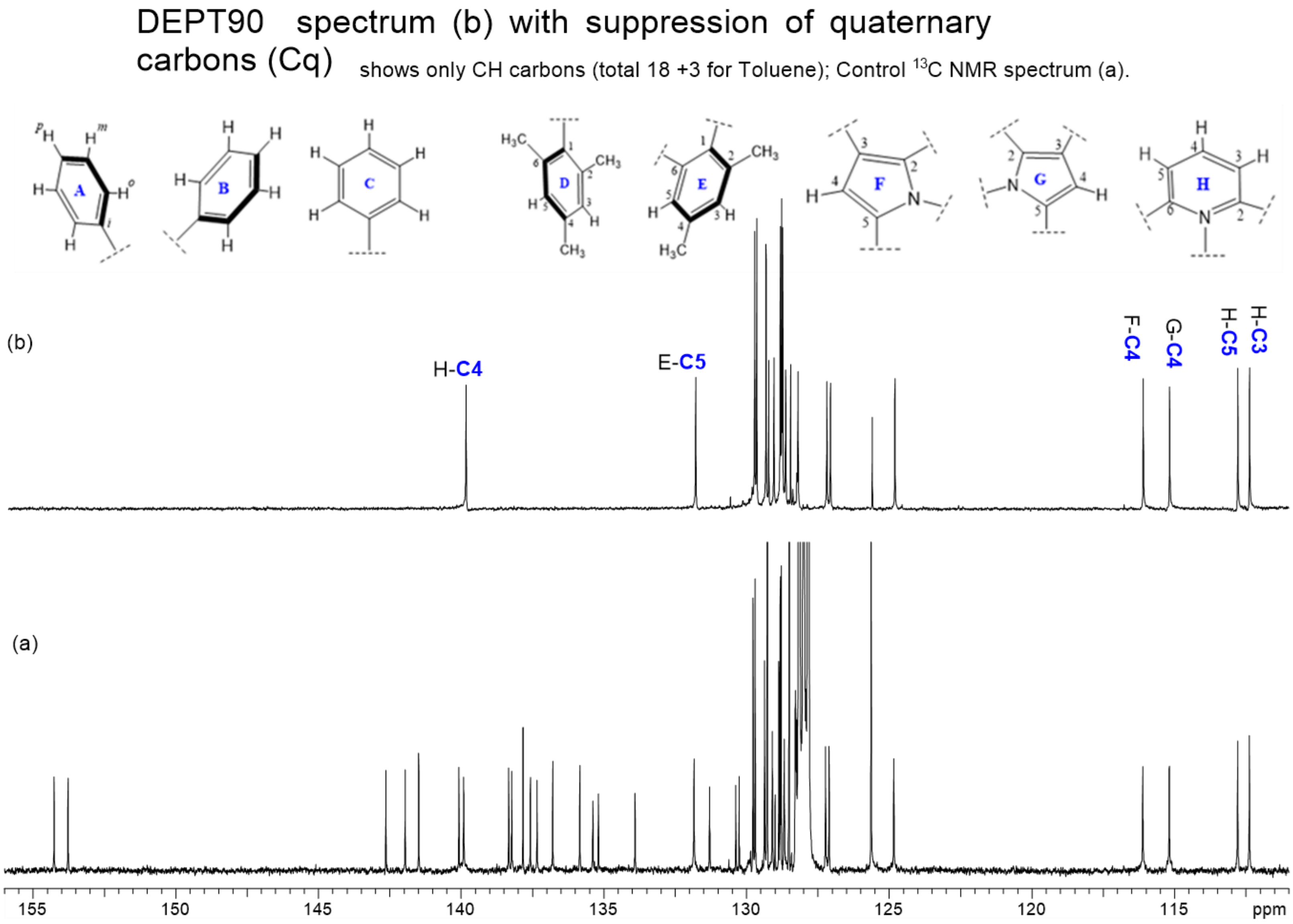

Figure S38. DEPT90 spectrum (b) of (cyclo-^{Mes}PDP^{Ph})HfBn with suppression of quarternary carbons.

Expanded portion of the DEPT90 spectrum (b) with suppression of quaternary carbons (Cq) shows only CH carbons (total 18 +3 for Toluene); Control ^{13}C NMR spectrum (a).

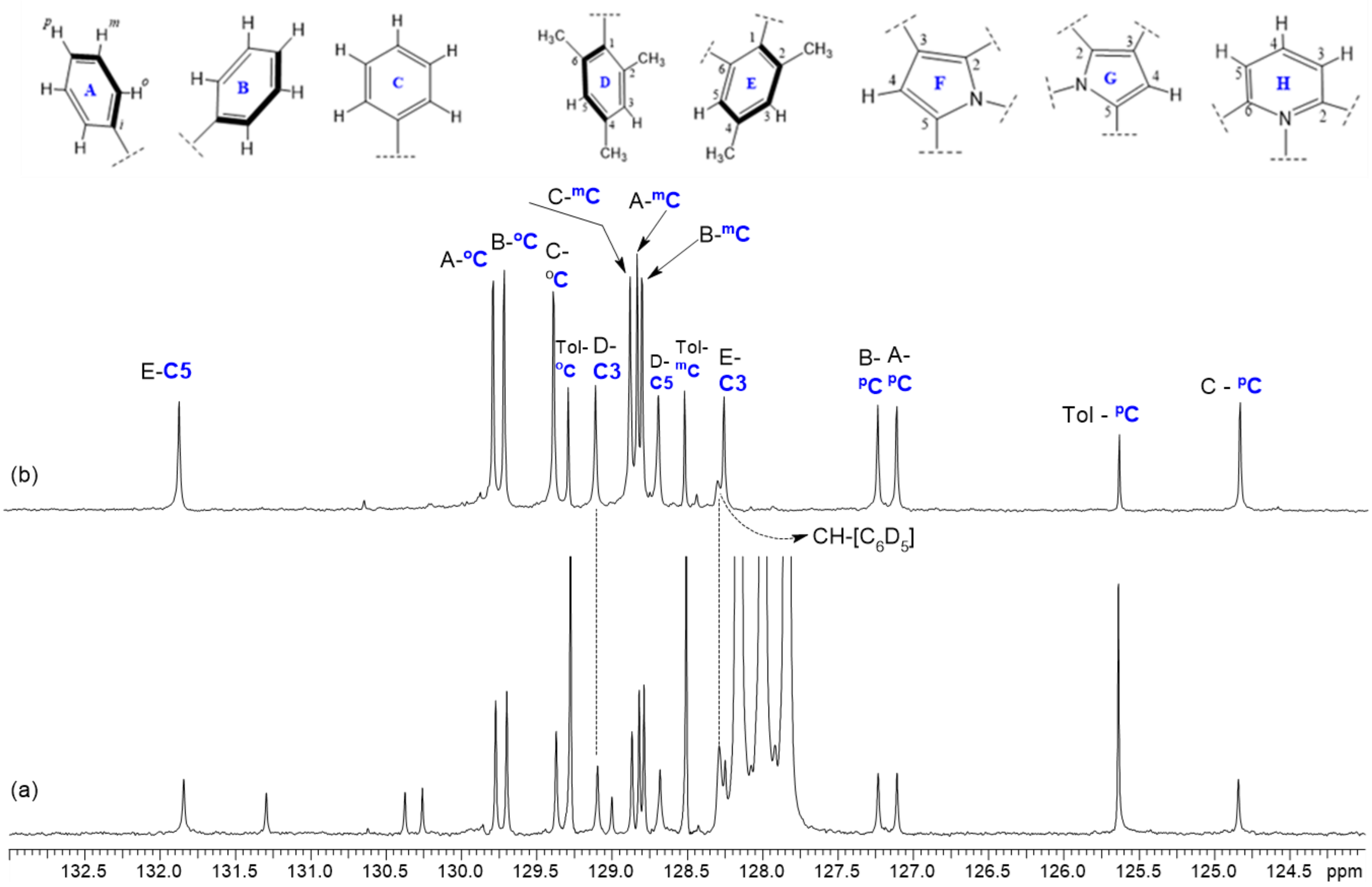

Figure S39. Expanded section of the DEPT90 spectrum (b) of (cyclo-^{Mes}PDP^{Ph})HfBn with suppression of quarternary carbons.

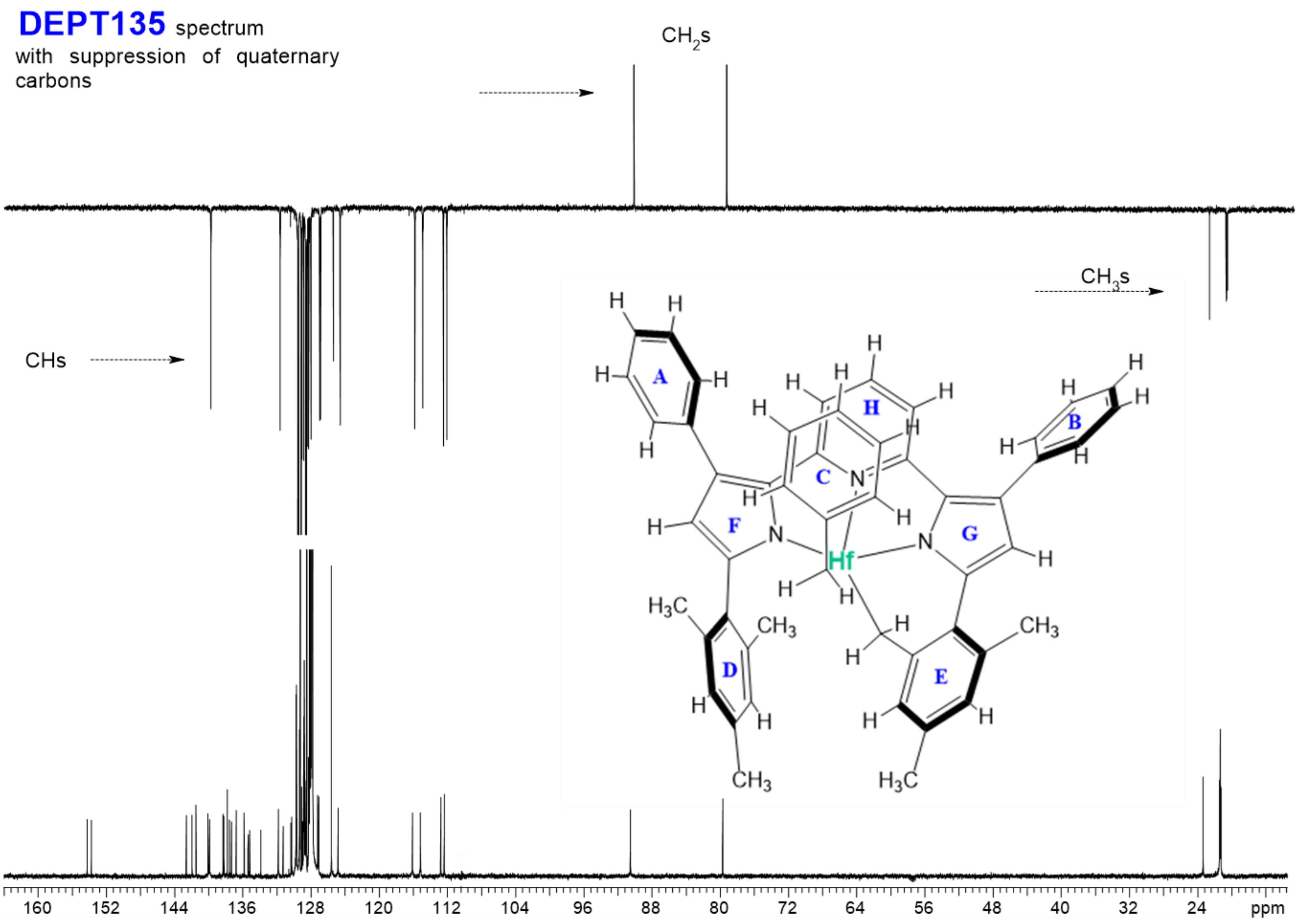

Figure S40. DEPT135 spectrum (top) of (cyclo-^{Mes}PDP^{Ph})HfBn with suppression of quarternary carbons.

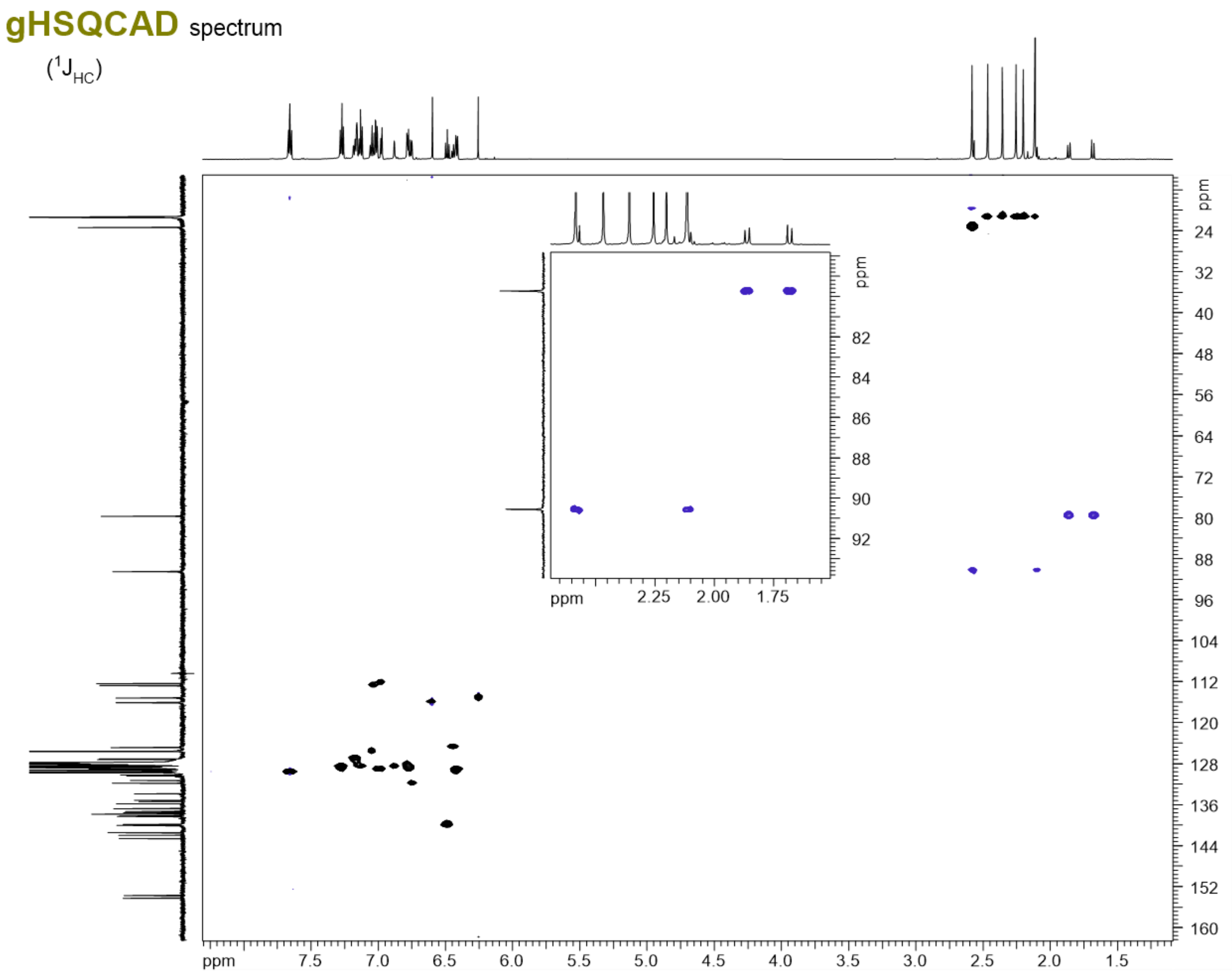

Figure S41. gHSQCAD spectrum of (cyclo-^{Mes}PDP^{Ph})HfBn.

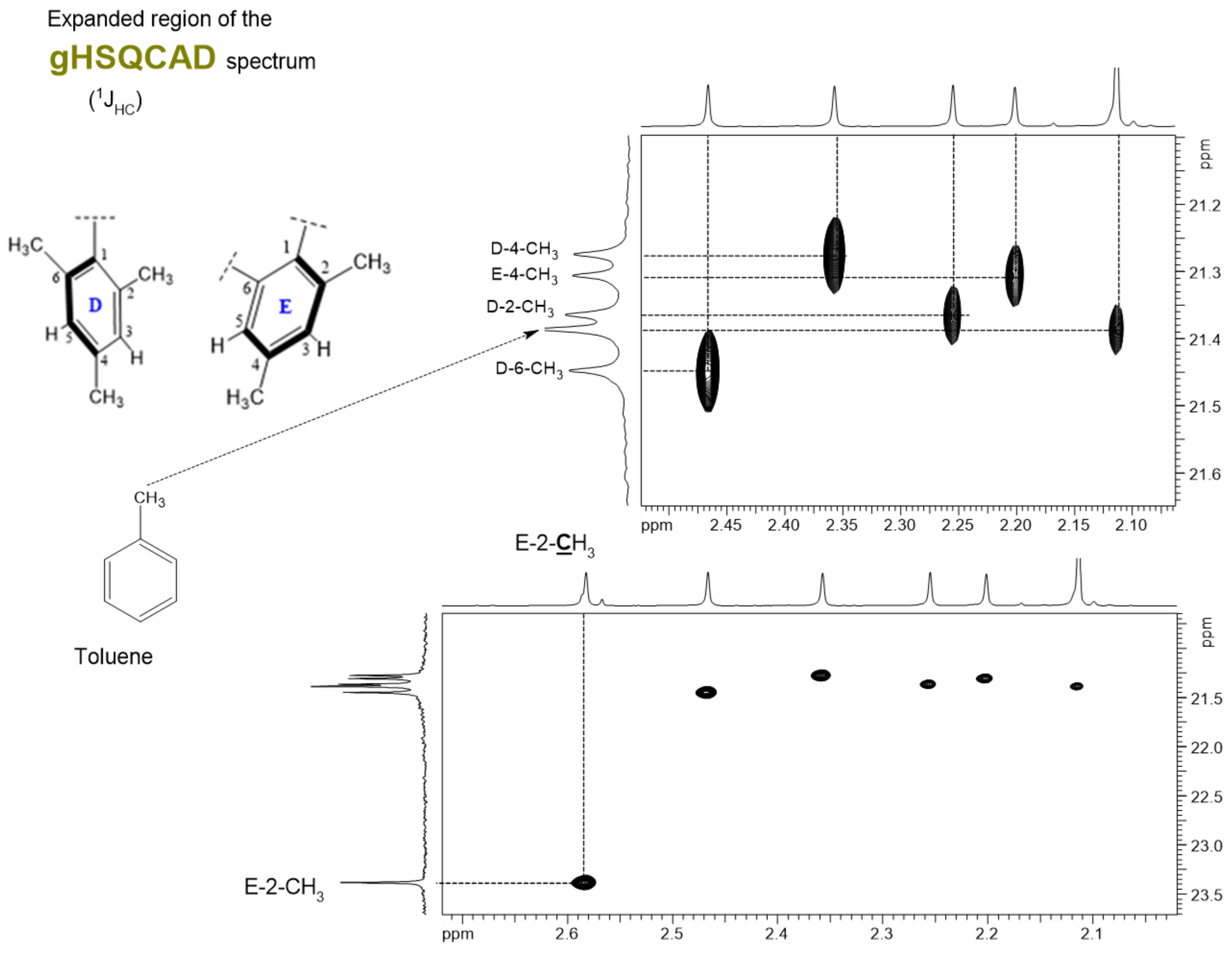

Figure S42. Expanded sections of the gHSQCAD spectrum of (cyclo-^{Mes}PDP^{Ph})HfBn.

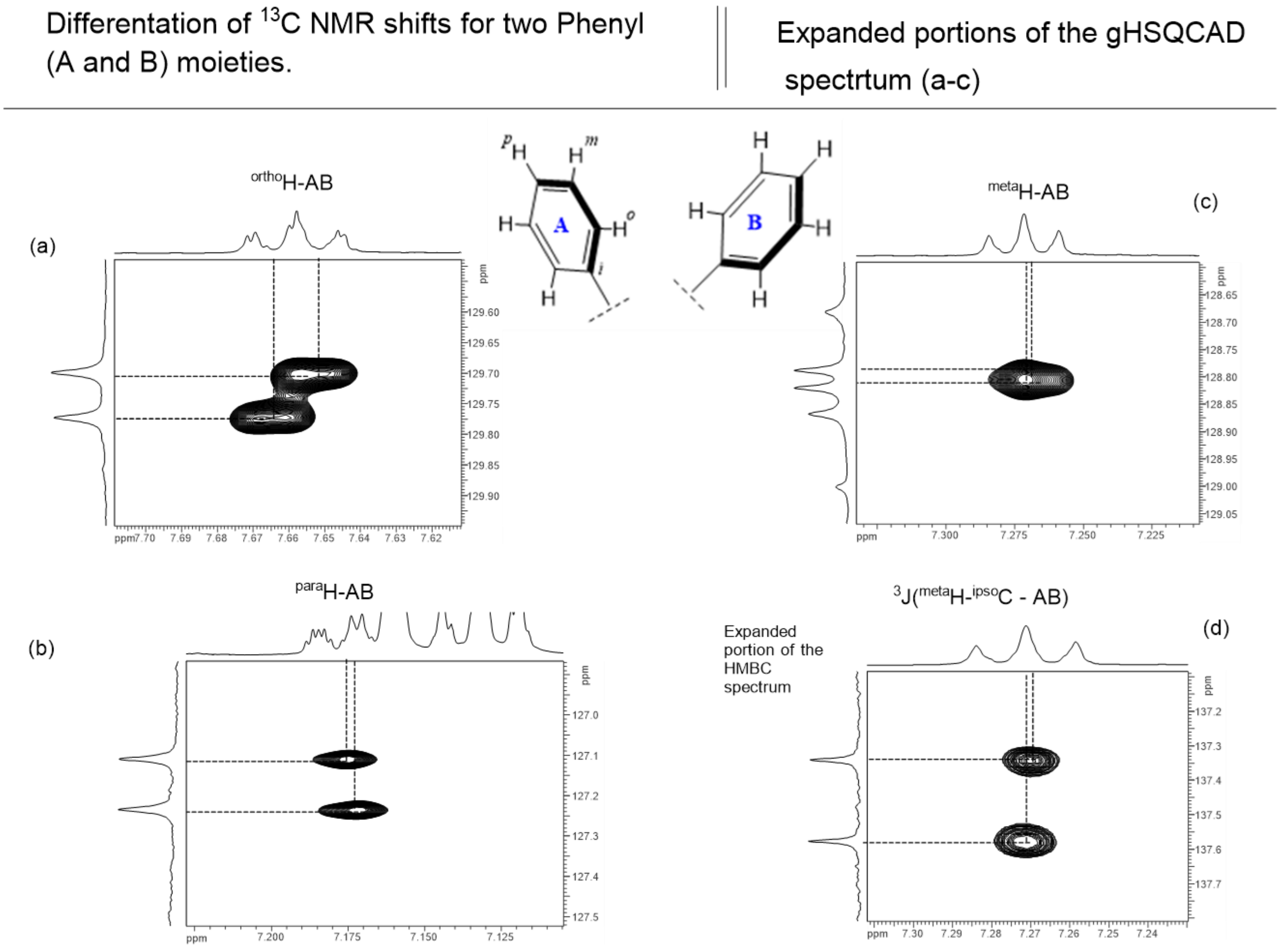

Figure S43. Expanded sections of the gHSQCAD spectrum of (cyclo-^{Mes}PDP^{Ph})HfBn.

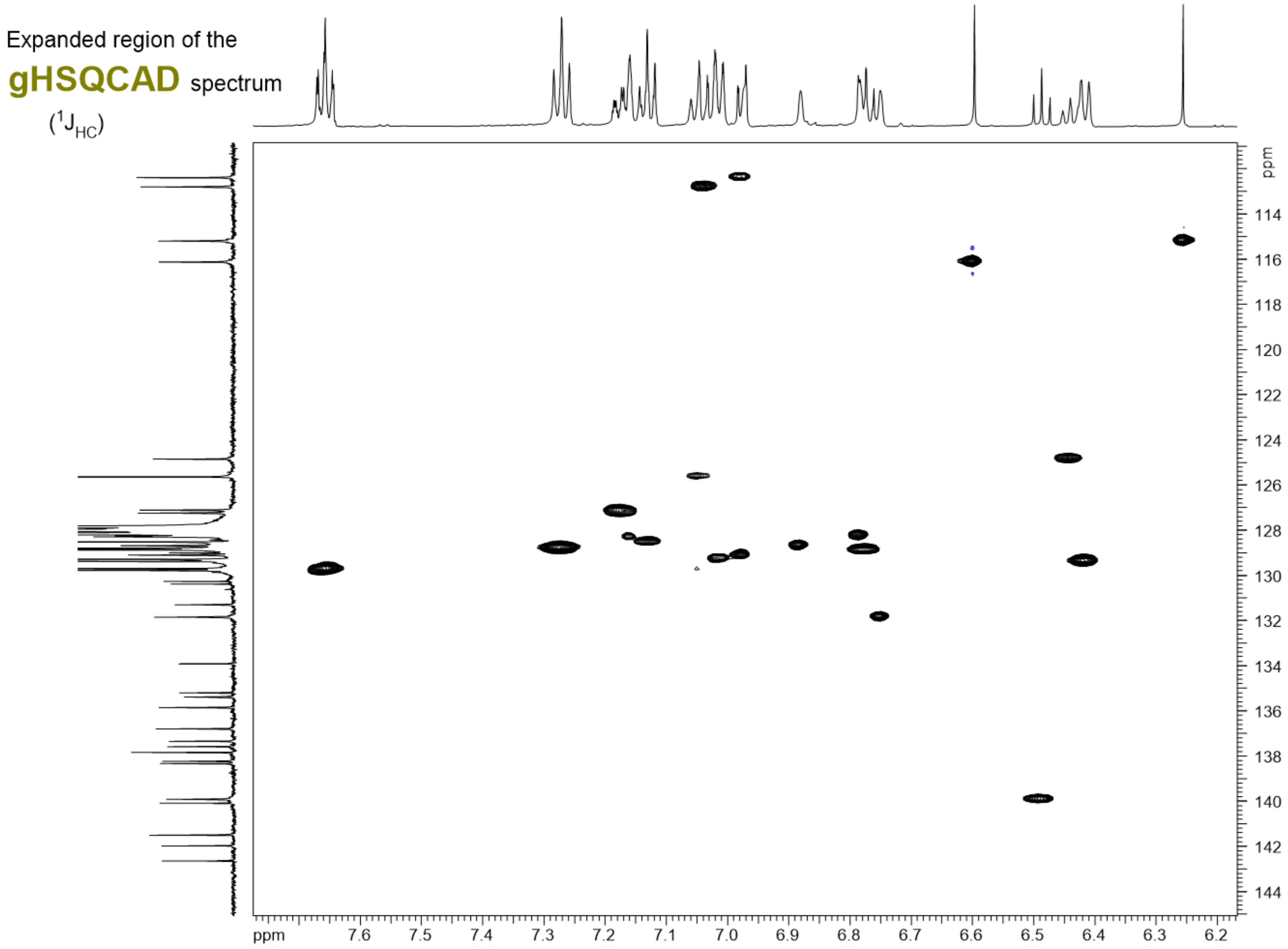

Figure S44. Expanded section of the gHSQCAD spectrum of (cyclo-^{Mes}PDP^{Ph})HfBn.

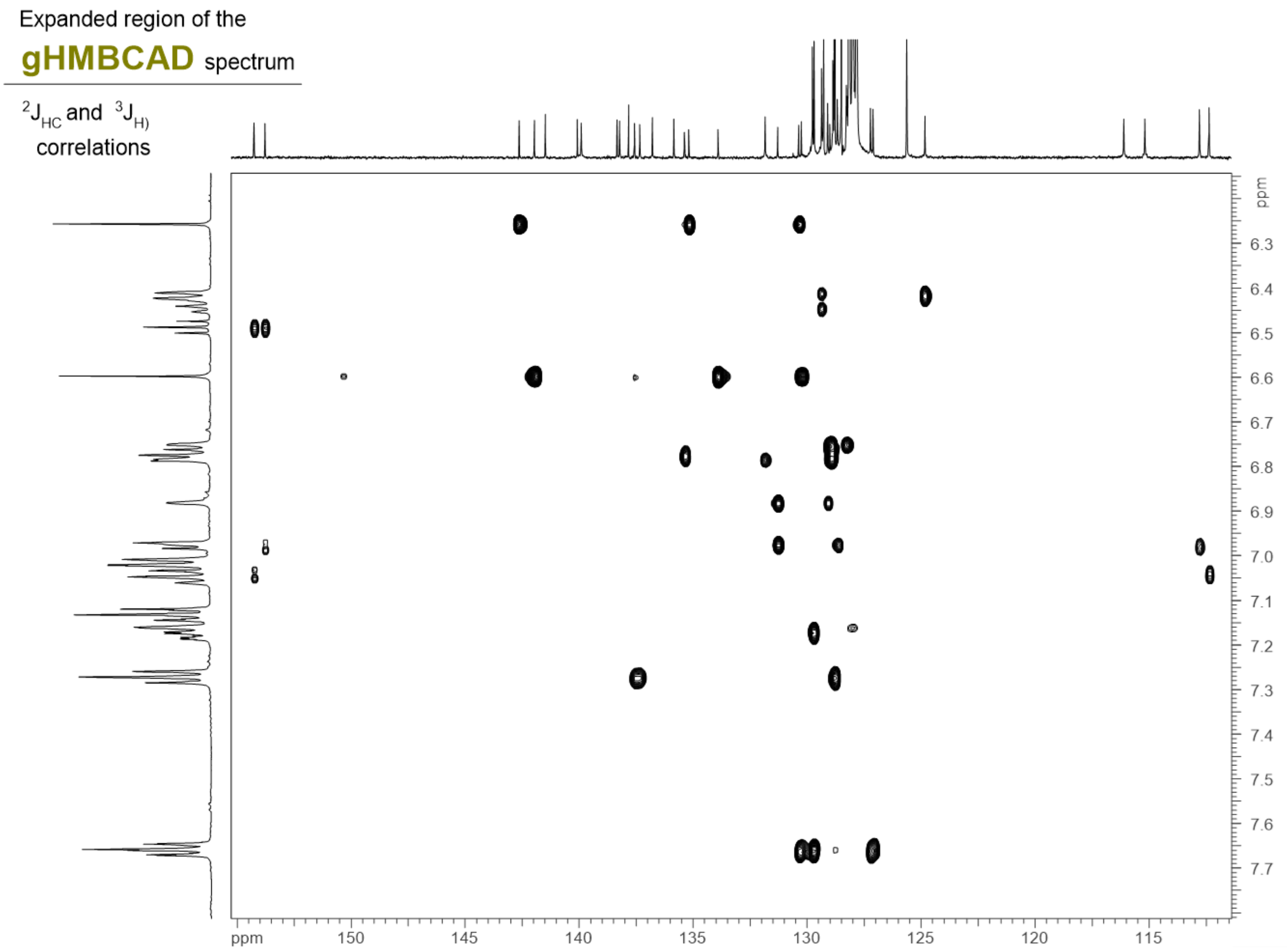

Figure S45. Expanded section of the gHMBCAD spectrum of (cyclo-^{Mes}PDP^{Ph})HfBn.

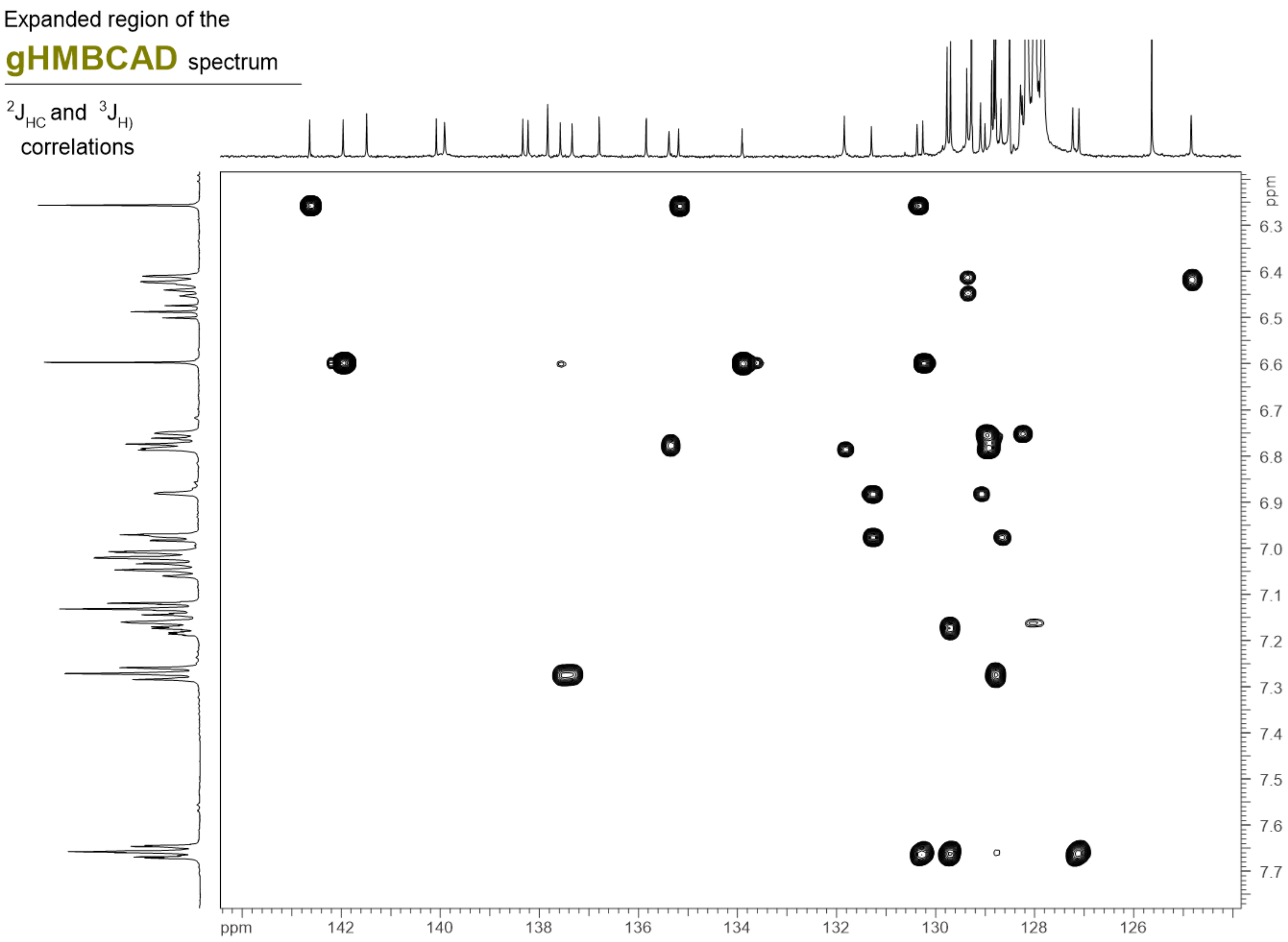

Figure S46. Expanded section of the gHMBCAD spectrum of (cyclo-^{Mes}PDP^{Ph})HfBn.

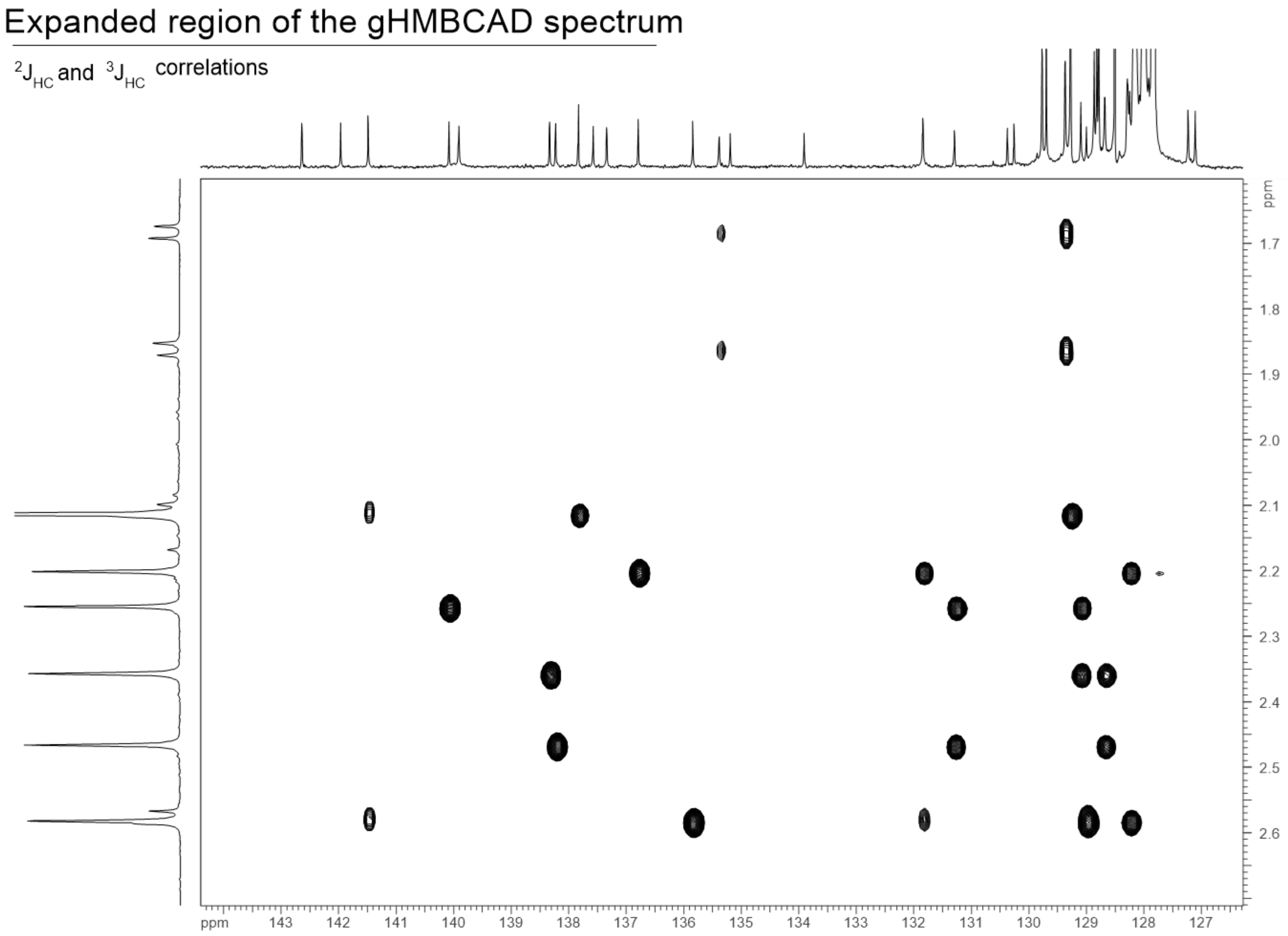

Figure S47. Expanded section of the gHMBCAD spectrum of (cyclo-^{Mes}PDP^{Ph})HfBn.

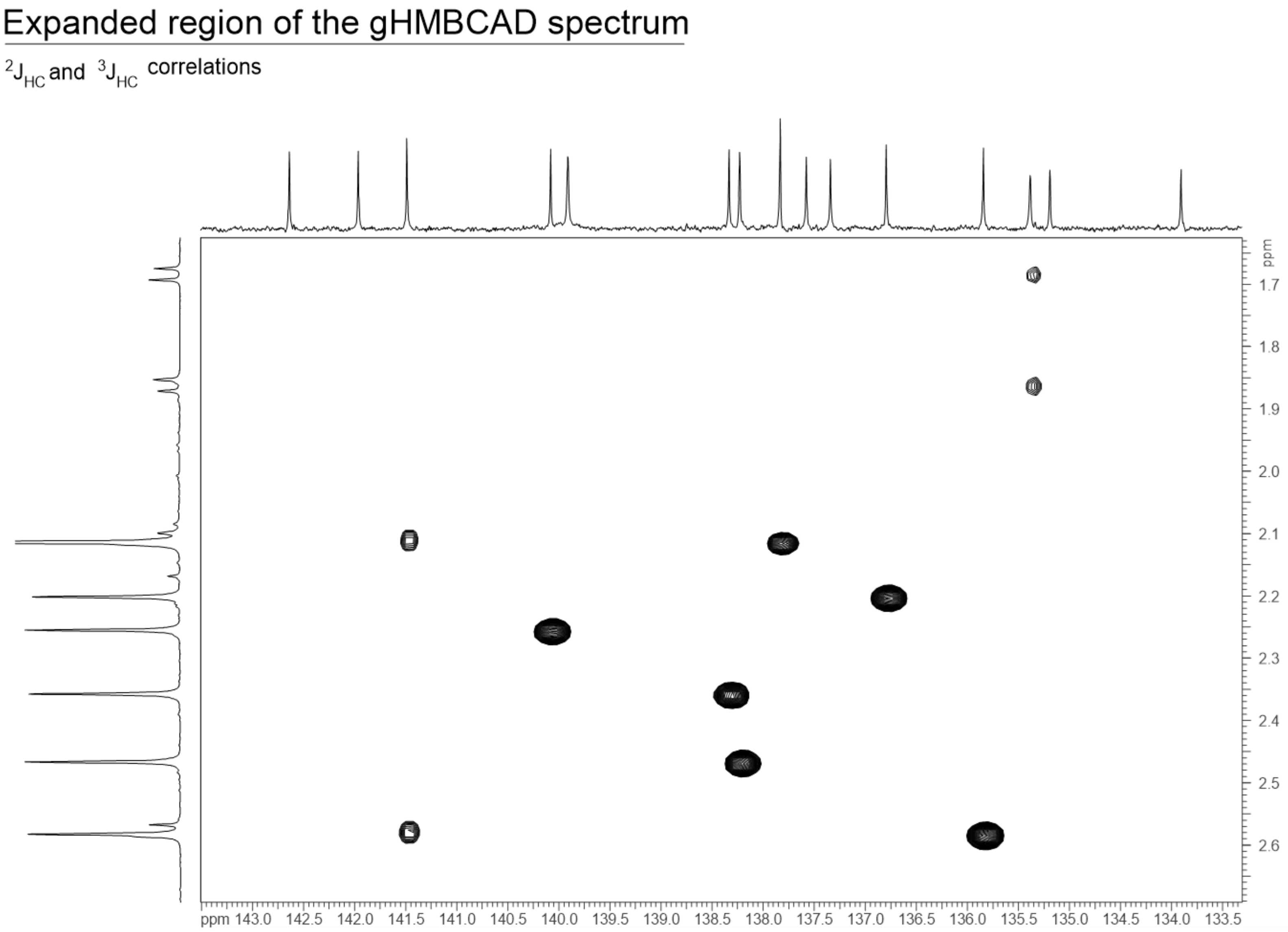

Figure S48. Expanded section of the gHMBCAD spectrum of (cyclo-^{Mes}PDP^{Ph})HfBn.

Differentation of ^{13}C NMR shifts for C2 and C6 carbons of Pyridine moiety based on $^4J_{HC}$ HMBC correlations

Expanded portions of the gHMBCAD spectrtum

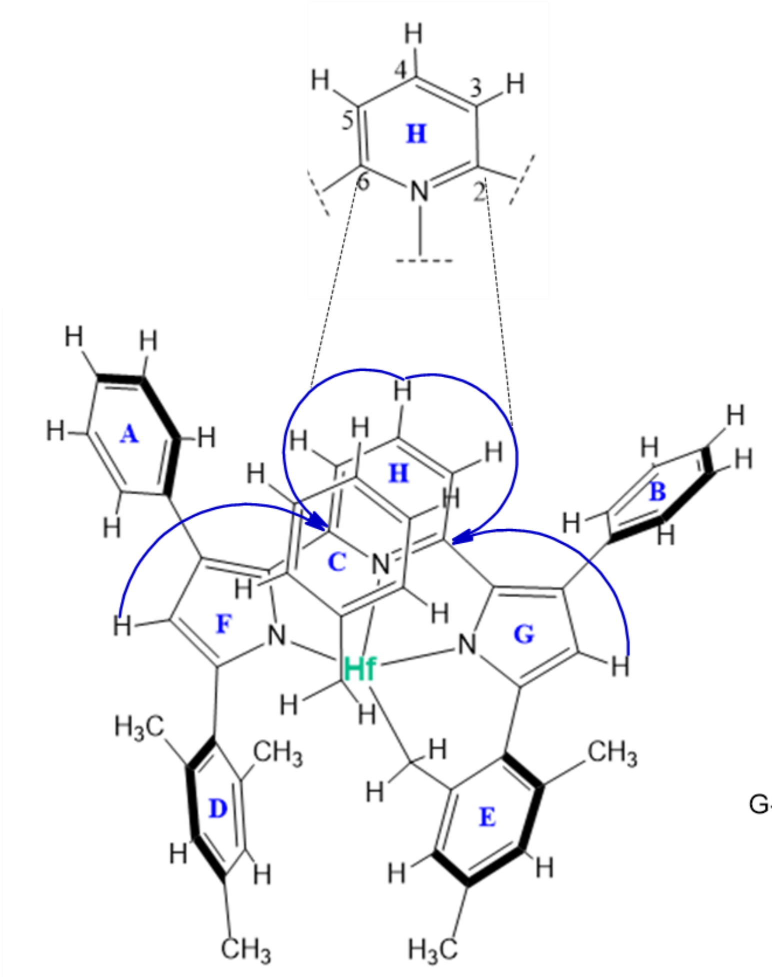

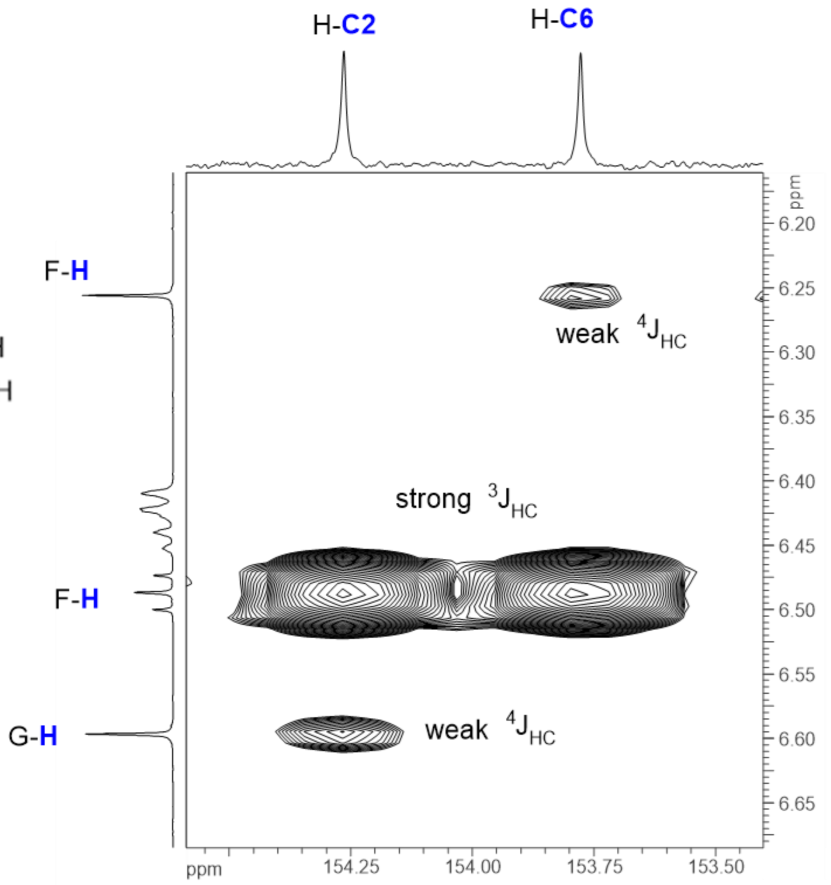

Figure S49. Expanded section of the gHMBCAD spectrum of (cyclo-^{Mes}PDP^{Ph})HfBn.

Differentation of ^{13}C NMR shifts for C2 and C6 carbons of Pyridine moiety based on $^{4}J_{HC}$ HMBC correlations

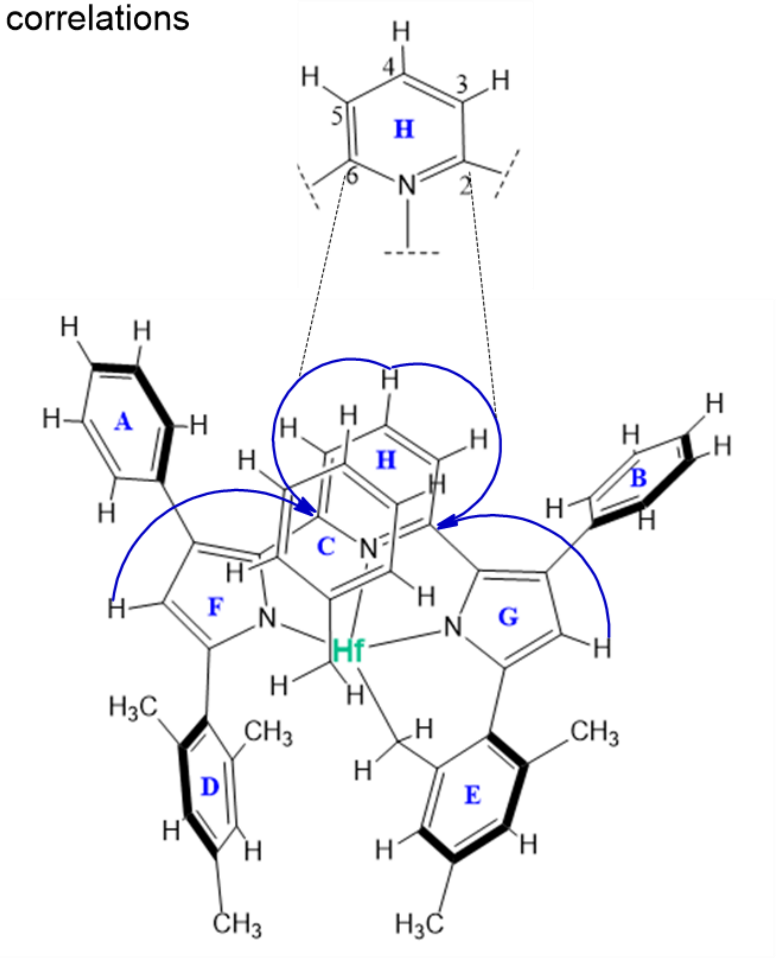

Expanded portions of the gHMBCAD spectrtum (a-c)

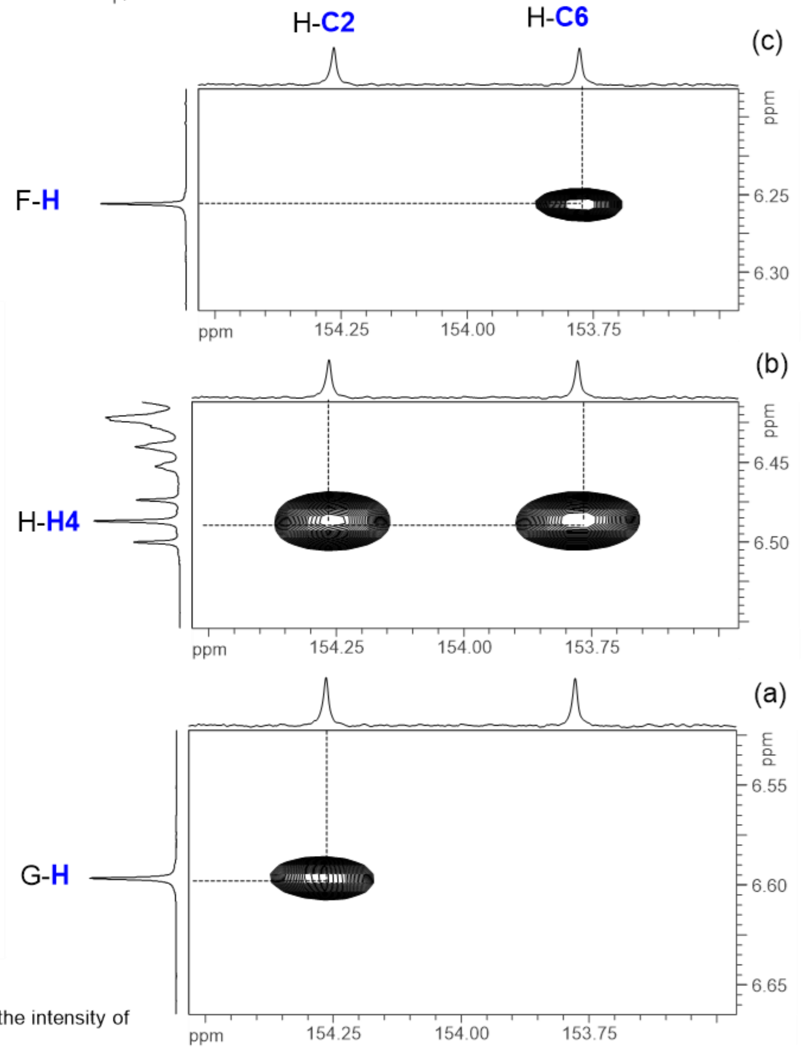

on the contour plot of the expanded portions of the HMBC spectra (a) and (c), the intensity of correlation peaks are magnified.

Figure S50. Expanded sections of the gHMBCAD spectrum of (cyclo-^{Mes}PDP^{Ph})HfBn.

3. Photophysical Measurements

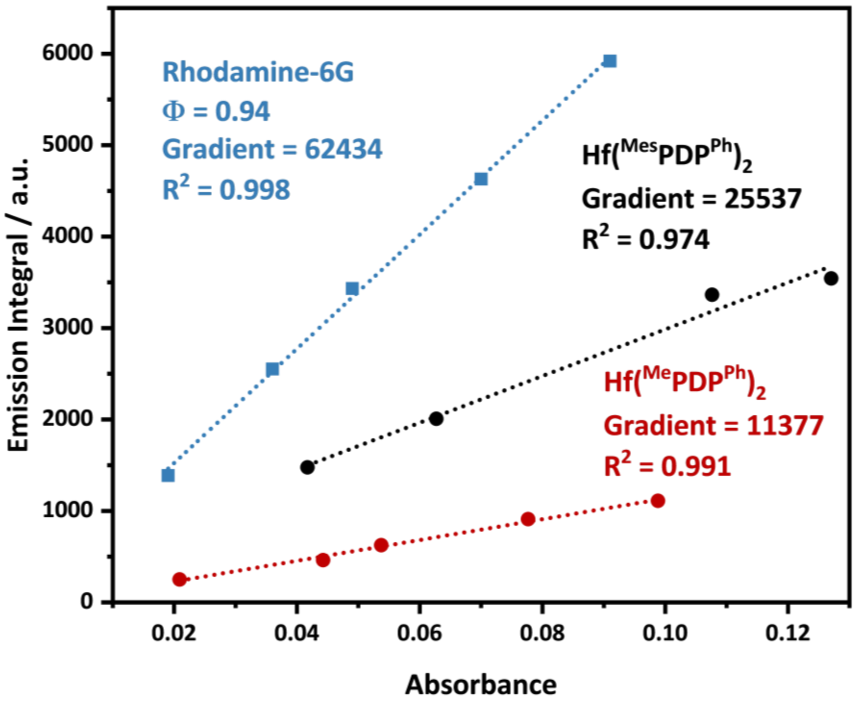

Figure S51. Plot of absorbance versus integration of the emission spectrum for Hf(^{Me}PDP^{Ph})$_2$ and Hf(^{Mes}PDP^{Ph})$_2$ in THF. The dotted lines represent linear fits of the data.

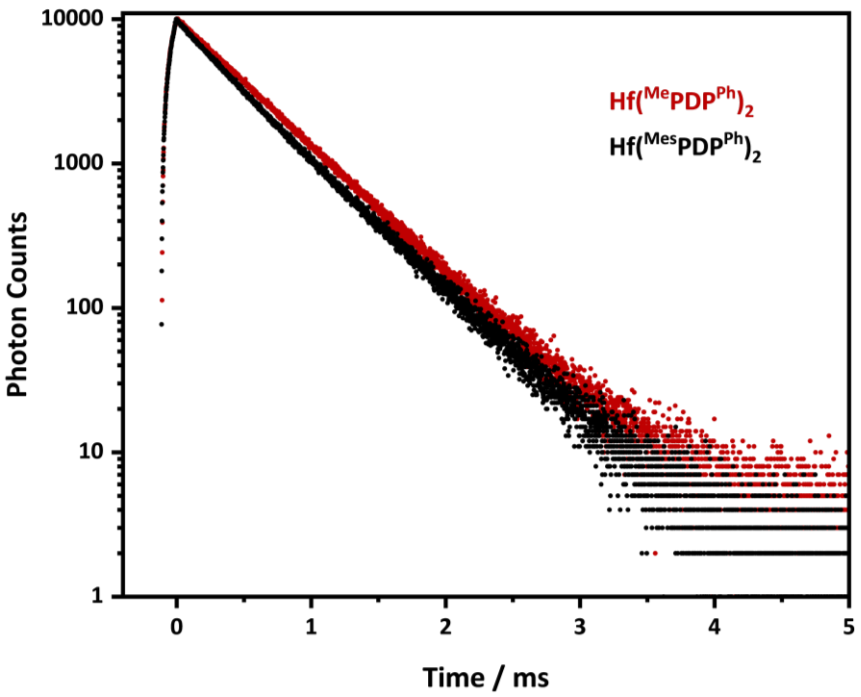

Figure S52. Time-resolved emission data for Hf(^{Me}PDP^{Ph})$_2$ and Hf(^{Mes}PDP^{Ph})$_2$ in THF solution at room temperature detected at $\lambda_{em\ max}$ upon excitation at 516 nm.
